# Factors associated with childhood undernutrition in poor Ethiopian households: Implications for public health interventions

**DOI:** 10.1371/journal.pone.0323332

**Published:** 2025-05-09

**Authors:** Biniyam Sahiledengle, Kingsley Emwinyore Agho, Yohannes Tekalegn, Degefa Gomora, Daniel Atlaw, Demisu Zenbaba, Fikreab Desta, Zinash Teferu, Girma Beressa, Telila Mesfin, Yordanos Sintayehu, Lillian Mwanri

**Affiliations:** 1 Department of Public Health, Madda Walabu University Goba Referral Hospital, Bale-Goba, Ethiopia; 2 Research Centre for Public Health Research, Equity and Human Flourishing, Torrens University Australia, Adelaide Campus, Adelaide, South Australia, Australia; 3 School of Health Sciences, Western Sydney University, Penrith, New South Wales, Australia; 4 Department of Midwifery, Madda Walabu University Goba Referral Hospital, Bale-Goba, Ethiopia; 5 Department of Human Anatomy, Madda Walabu University Goba Referral Hospital, Bale-Goba, Ethiopia; 6 Department of Medicine, Madda Walabu University Goba Referral Hospital, Bale-Goba, Ethiopia; Woldia University, ETHIOPIA

## Abstract

**Background:**

Childhood undernutrition is a significant public health concern linked to poverty. Despite the persistent high burden of childhood undernutrition in Ethiopia, there is a lack of robust evidence identifying factors associated with undernutrition in under-five children from poor households in the nation. This study aimed to identify the determinants of childhood undernutrition among children living in poor households in Ethiopia.

**Methods:**

The 2005, 2011 and 2016 Ethiopian Demographic and Health Surveys were combined, and analysis was restricted to children aged 0–59 months from poorer and poorest households, yielding a weighted sample of 12,466 analysed. The adverse nutritional status indicators of child nutritional status: height-for-age z-scores (HAZ), weight-for-age z-scores (WAZ), and weight-for-height z-scores (WHZ) were the outcomes of interest. The child’s HAZ, WHZ, and WAZ were below -2 standard deviations (SD) were categorized as binary and into stunted, wasted, and underweight, respectively. Multilevel mixed-effect logistic regression analyses were conducted to examine factors associated with childhood undernutrition in poor households.

**Results:**

The prevalence of stunting, wasting and underweight among children aged 0–59 months in poor households was 47.5% (95% CI: 46.5–48.4), 12.7% (95% CI: 12.1–13.3), and 32.8% (95% CI: 31.9–33.7), respectively. The most significant factors positively associated with stunting, wasting, and being underweight comprised of male gender, younger age, having diarrhea two week before each survey, children perceived as smaller by their mothers (stunted and wasted only), children of uneducated mothers (stunting and underweight only), maternal short stature (stunting and underweight only), and children from households having unimproved sanitation facility (stunting and underweight only). The odds of wasting were significantly higher among children who lived in urban areas, children from female-headed households and those children who had fever two weeks before each survey.

**Conclusion:**

Child undernutrition in poor Ethiopian households is significantly higher than the national average, highlighting a critical public health issue. Urgent intervention focusing on the identified risk factors, such as sanitation, maternal education, and childhood diarrhea is needed, to improve child nutrition and well-being in disadvantaged households.

## Background

Undernutrition in children is a significant public health scourge that manifests in three primary ways: wasting, stunting, and being underweight [1]. Wasting refers to low weight-for-height, typically resulting from acute food shortages or illnesses. On the other hand, stunting is characterised by low height-for-age and often occurs due to prolonged nutritional deprivation. Being underweight is defined as having a low weight for one’s age beyond [2]. These various forms of undernutrition not only jeopardise the physical health of affected children but also severely hamper their cognitive development throughout childhood and beyond [3,4].

Globally, in 2020, approximately 149 million children under five years of age experienced stunting, with an estimated 45 million suffering from wasting [1]. Sub-Saharan Africa (SSA) is home to nearly two-fifths of stunted children and more than a quarter of wasting children under the age of five years [2,5]. Notably, most undernourished children reside in East Africa, where Ethiopia is located [6,7]. Ethiopia and other SSA nations have committed to eradicating all forms of undernutrition by 2030; however, the rate of progress has been insufficient [8,9]. Additionally, the African Regional Nutrition Strategy (ARNS) adopted by member states of the African Union, has targets to reduce the number of stunted children younger than five years of age by 40%, by 2025 [10].

Despite concerted attempts, childhood undernourishment remains a critical issue in many SSA countries, including Ethiopia. One of the primary underlying factors is deeply entrenched poverty, which is closely linked to the persistent burden of undernutrition [11,12]. According to the World Bank’s most recent data, approximately 40% of the population resides below the extreme poverty threshold in SSA, representing two-thirds of the global extremely poor population [12]. Similarly, a recent report from the Oxford Poverty and Human Development Initiative (OPHI) revealed that about 68.7% of Ethiopia’s population is multidimensionally poor, underscoring the widespread deprivation affecting millions [13]. While the burden of childhood undernutrition is well-documented, the specific drivers of undernutrition in poverty-affected households in Ethiopia remain insufficiently investigated.

Over the past two decades, Ethiopia has made significant efforts to combat childhood undernutrition through various initiatives and strategies [14,15]. Notable among these are the “*Seqota Declaration”*, which aims to eliminate stunting in children under two by 2030 [16], and the National Food and Nutrition Policy (FNP), which seeks to enhance food security and improve nutrition nationwide [17]. Despite these efforts, significant challenges remain in reducing childhood undernutrition. Achieving the 2030 target requires an annual reduction of over 3%, which seems highly challenging given the current trends and persistent inequalities [18,19].

Previous studies have investigated different forms of childhood undernutrition in Ethiopia using nationally representative datasets [20–26]. Some have specifically focused on stunting [27], wasting [28], and underweight [29,30], while others have explored gender, wealth, and urban-rural disparity [22,31–34], or spatial variations in childhood undernutrition [21,35–38]. Prior studies have also identified multiple factors influencing childhood undernutrition, including child-related factors such as gender, age, birth weight, birth order, recent diarrheal episodes [20,23,27,39–42], type of birth [43], and preceding birth interval [44]. Other determinants include dietary factors like food diversity score [45], maternal characteristics such as education level [20,39], and maternal body mass index (BMI) [43,46], as well as community-level factors, including place of residency [24], and environmental conditions [20,46,47]. Further, studies have identified wealth-related inequalities in the burden of childhood undernutrition in Ethiopia, with children from lower wealth quantiles facing substantially higher risks [22,32,33,48]. However, no research has specifically examined the burden and underlying determinants of childhood undernutrition exclusively among disadvantaged, poverty-affected households in Ethiopia.

Given the persistently high burden of undernutrition in disadvantaged households, there is a call for further assessment of its key drivers within these vulnerable populations to inform more targeted and effective interventions [49]. While household wealth indices are valuable for identifying socioeconomic disparities, they do not fully capture the complexity of childhood undernutrition, especially in disadvantaged, poverty-affected households. These indices highlight existing inequalities but leave a gap in understanding the specific factors contributing to undernutrition in these populations. This study seeks to fill this gap by examining the key determinants of childhood undernutrition (stunting, wasting, and underweight) in poor households in Ethiopia. To our knowledge, no study has specifically quantified the burden and determinants of childhood undernutrition in these disadvantaged households using nationally representative data. Understanding these factors is crucial for informing policy and interventions to reduce undernutrition and help achieve SDG Goal 2.

## Methods

### Study setting

Ethiopia is one of the oldest nations in the world, situated in the Horn of Africa and sharing borders with Djibouti, Eritrea, Sudan, Kenya, and Somalia. Administratively, Ethiopia is divided into 12 geographical regions (i.e., Afar, Amhara, Tigray, Benishangul-Gumuz, Central Ethiopia, Gambela, Harari, Oromia, Sidama, Somali, South Ethiopia and Southwest Region) and two major administrative cities—Addis Ababa and Dire Dawa. The Ethiopia Demographic and Health Survey (EDHS) is a comprehensive national survey that collects key data on population health and demographic indicators. The EDHS is conducted by the Central Statistical Agency (CSA) with support from international organizations. The survey is conducted periodically, with the most recent one in 2016 [18,50,51].

### Data source and study design

This cross-sectional study examined data from Ethiopia Demographic and Health Surveys (EDHS) from 2005, 2011, and 2016. The EDHSs is a nationally representative population-based survey conducted every five years as a part of the Demographic and Health Surveys (DHS) Program. The EDHSs employ a two-stage multistage sampling to establish a representative sample of households at the national and regional levels. Details descriptions on sampling and sample design are presented in EDHS reports [18,50,51]. In this analysis, we used data from children aged 0–59 months and their mothers aged 15–49 years living in poor households. Our analysis included a total weighted sample of (n = 12,466) children under five years from poor households.

### Study variables and measurements

Stunting is defined as height-for-age z-scores (HAZ) below minus two standard deviations (-2SD) from the median of the reference population. Children whose weight-for-height (WHZ) is below minus two standard deviations (-2SD) from the median of the reference population are too thin for their height or wasted. While children whose weight-for-age (WAZ) measures below minus two standard deviations (-2SD) from the median of the reference population are underweight for their age. All anthropometric outcome variables were constructed based on the 2006 World Health Organization (WHO) child growth standards [52].

The household wealth index has frequently been used as an indicator to assess household expenditures and incomes. Furthermore, in large surveys such as the DHS, it is a powerful indicator of poverty level. The EDHS developed the wealth index by principal component analysis (PCA), and a detailed explanation of how the DHS created the wealth quantiles is in well-documented reports [18,50,51]. In brief, households are given scores based on the number and kinds of consumer goods they own, ranging from a television to a bicycle or car, in addition to housing characteristics such as the source of drinking water, toilet facilities, and flooring materials. These scores are generated using PCA, and the wealth index is categorized into five quintiles: poorest, poor, middle, richer, and richest. Following related papers, the poorer and poorest were classified as “poor households”, the literature suggests that children in these households have similar health outcomes [49,53,54]. Our analysis was restricted to children aged 0–59 months from poorer and poorest households. This study included children who had valid and complete anthropometric measurements. Accordingly, a total weighted sample of 12,466 children aged 0–59 months was included.

### Outcome variables

The three adverse nutritional status indicators of a child’s nutritional status (also referred to as stunting, wasting, and underweight) were the outcome variables with binary categories.

### Independent variables

Based on the review of the literature and available data contained in the EDHS datasets, potential characteristics related to the child, mothers, household and community levels factors were included in our analysis as follows:(i) child factors, sex of the child (male or female), age of the child (0–5 months, 6–11 months, 12–23, 24–35, and 36–59 months), birth order (firstborn,2–4, and 5+), the perceived size of the child at birth (large, average, and small), currently breastfeeding (yes, no), early initiation of breastfeeding (yes, no), having diarrhea and fever in the last 2 weeks (yes, no), full vaccination (yes, no), receiving vitamin A in the last 6 months (yes, no), and birth interval (short (i.e., less than 33 months) and non-short (≥ 33 months). (ii) maternal factors: age of the mother (15–17, 18–24, 25–34, or ≥ 35), mother’s education (no education, primary and above), mother’s currently working (yes, no), maternal stature (normal (≥ 155 cm), short (145 to 154.9 cm), very short (<145 cm)), mother’s body mass index (BMI) (kg/m^2^) (<18.5 kg/m^2^, 18.5 to 24.9 kg/m^2^, and 25 + kg/m^2^), maternal anaemia (yes, no), place of delivery (home, health facility), and listening to radio/ watching television (yes, not at all). (iii) household factors: the sex of the household head (male, female), number of household members (1–4, 5+), household sanitation facility (improved, unimproved, and open defecation), source of drinking water (improved, unimproved), child stool disposal (safe, unsafe), and time to get a water source (on-premises, ≤ 30 min round-trip fetching times, 31–60 min round-trip fetching times, and over 60 min round-trip fetching times), (iv) community-level factors: place of residence (rural, urban), contextual regions (agrarian, pastoralist, or metropolises/city administrations), and survey years.

### Data analysis

All analyses used STATA/MP version 14.1 (Stata Corp, College Station, TX, USA). The *‘Svy’* commands were employed to allow for adjustments for the cluster-sampling design and weight. We conducted frequency tabulations to describe the data used in the study and the distributions of stunting, wasting, and underweight by background characteristics. The EDHS data were hierarchical, i.e., children were nested within households, and households were nested within clusters (or enumeration areas (EAs)). This violated the assumption of independence of observations and equal variance across the clusters. Hence, we estimated a two-level model with the child data as level 1 and clusters as level 2 [55]. First, a bivariable multilevel logistic regression analysis that adjusts for sampling weights was conducted to identify factors and variables associated with childhood undernutrition (i.e., stunting, wasting, and underweight). Multilevel bivariable logistic regression analyses was conducted and variables with a p-value < 0.25 retained and were selected to enter multilevel multivariable logistic regression models to estimate their independent association with childhood undernutrition. Then, we performed a four-model as part of the multilevel multivariable logistic regression analysis to identify the association between individual and community-level factors and childhood undernutrition. The empty model without any explanatory variables was run to detect the presence of a possible contextual effect (*model I*); the second with individual-level variables (*model II*), the third with community-level variables (*model III*), and the fourth with both individual and community-level variables (*model IV*). Multicollinearity among the independent variables was checked before their inclusion in the final regression model. Multicollinearity among independent variables was assessed by using the variance inflation factor (VIF), the VIF < 5 was considered suitable [56].

#### Model building.

The likelihood ratio (LR) test, intra-class correlation coefficient (ICC), and median odds ratio (MOR) were used to assess variation between clusters. The ICC for stunting, wasting, and underweight in the null model were 4.52%, 4.96%, and 4.27%, respectively. Despite ICC values below 10%, the significant LR test indicated that a multilevel binary logistic regression model was a better fit than classical regression. Given the hierarchical structure of the EDHS data (children nested within households and households within clusters), a two-level multilevel model was estimated [57]. The MOR measures variation in undernutrition status between clusters on the odds ratio scale. It is defined as the median odds ratio between a cluster with a high likelihood of undernutrition and a cluster with a lower risk, determined by randomly selecting individuals from two different clusters (EAs). Model comparisons were performed using the deviance information criteria (DIC). The model with the lowest DIC was considered the best-fit model. The findings of the study were reported as adjusted odds ratios (AORs) along with 95% confidence intervals (CIs) at a significance level of p < 0.05.

### Ethics statement

We used data from datasets provided by the Demographic Health Surveys (DHS) program, freely publicly available online in an open-access repository (http://dhsprogram.com) requiring no ethics approval. The Measure DHS public-use datasets do not in any way allow respondents to be identified. Therefore, for this study, ethics approval and consent to participate are not applicable because secondary data analysis did not involve interaction with the participants, and datasets were publicly available. Ethical clearance for the original EDHS was approved by the Ethiopian Public Health Institute Review Board, Ethiopian Health and Nutrition Research Institute (EHNRI) Review Board, the National Research Ethics Review Committee (NRERC) at the Federal Democratic Republic of Ethiopia Ministry of Science and Technology, the ICF Macro Institutional Review Board, and the Centers for Disease Control and Prevention (CDC). The EDHS publications state that each respondent gave informed written consent permission to participate. All procedures were followed in accordance with the Helsinki declarations. Further information about the DHS’s ethical standards can be accessed online (http://goo.gl/ny8T6X).

## Results

### Characteristics of the study population

In this study, children aged 0–59 months from poorer and poorest households were analysed yielding a weighted sample of 12,466 (EDHS-2005, n = 2,150; EDHS-2011, n = 5,304; EDHS-2016, n = 5,012). Of these, 51.7% were male, 42.2% were in the age category of 36–59 months, and 72.4% were currently breastfeeding. The prevalence of having diarrhea and fever in the last two weeks before the survey among under-five children was 13.8% and 16.1%, respectively. Most of the mothers were uneducated (82.2%) and 75.3% were not working (Table 1).

**Table 1:** The characteristics of the study participants in the EDHS of 2005, 2011, and 2016, Ethiopia (n = 12,466).

Variables	EDHS-2005	EDHS-2011	EDHS-2016	Pooled (EDHS 2005–2016)
***Child factors***				
**Sex**				
Male	1,243 (51.6)	2,859 (51.4)	2,752 (52.2)	6,580 (51.7)
Female	1,165 (48.4)	2,703 (48.6)	2,518 (47.8)	6,143 (48.3)
**Age (months)**				
< 6	248 (10.3)	609 (10.9)	530 (10.1)	1,355 (10.7)
6-11	236 (9.8)	582 (10.5)	510 (9.7)	1,287 (10.1)
12-23	357 (18.9)	975 (17.5)	957 (18.3)	2,306 (18.2)
24-35	441 (18.3)	958 (17.2)	1,066 (20.4)	2,375 (18.7)
36-59	1,025 (42.6)	2,438 (43.8)	2,164 (41.4)	5,359 (42.2)
**Size of the child at birth**				
Larger	749 (31.2)	1,742 (31.5)	1,539 (29.5)	3,856 (30.5)
Average	909 (37.9)	2,021 (36.6)	2,146 (41.1)	4,885 (38.7)
Small	736 (30.8)	1,763 (31.9)	1,533 (29.4)	3,895 (30.8)
**Birth order**				
First born	363 (15.1)	973 (17.5)	850 (16.1)	2,063 (16.2)
2-4	1,010 (41.9)	2,333 (41.9)	2,243 (42.6)	5,378 (42.3)
5+	1,035 (43.0)	2,255 (40.5)	2,177 (41.3)	5,283 (41.5)
**Full vaccination**				
Yes	205 (10.3)	771 (16.4)	595 (21.3)	1,571 (16.6)
No	1,786 (89.7)	3,941 (83.6)	2,193 (78.7)	7,872 (83.4)
**Vitamin A last 6 months**				
Yes	1,019 (48.2)	2,176 (45.0)	1,788 (38.4)	4,961 (42.9)
No	1,095 (51.8)	2,654 (55.0)	2,868 (61.6)	6,592 (57.1)
**Currently breastfeeding**				
Yes	1,763 (73.2)	4,048 (72.8)	3,620 (68.7)	9,219 (72.4)
No	645 (26.8)	1,514 (27.2)	1,650 (31.3)	3,505 (27.5)
**Early initiation of breastfeeding**				
Yes	1,026 (69.0)	1,694 (49.8)	2,327 (72.6)	4,928 (62.3)
No	461 (31.0)	1,710 (50.2)	878 (27.4)	2,986 (37.7)
**Birth interval**				
Less than 33 months	1,583 (65.7)	3,835 (68.9)	3,700 (70.2)	8,770 (68.9)
≥ 33 months	825 (34.3)	1,727 (31.1)	1,570 (29.8)	3,954 (31.1)
**Diarrhoea**				
Yes	422 (19.6)	664 (13.7)	528 (11.2)	1,614 (13.8)
No	1,734 (80.4)	4,172 (86.3)	4,187 (88.8)	10,075 (86.2)
**Fever**				
Yes	404 (18.7)	850 (17.6)	623 (13.2)	1,877 (16.1)
No	1,753 (81.3)	3,981 (82.4)	4,101 (86.8)	9,816 (83.9)
**Cough**				
Yes	366 (16.9)	942 (19.5)	889 (18.8)	2,197 (18.8)
No	1,791 (83.1)	3,897 (80.5)	3,836 (81.2)	9,506 (81.2)
***Parental factors***				
**Mother’s age**				
15-17	19 (0.8)	39 (0.7)	64 (1.2)	113 (0.9)
18-24	598 (24.8)	1,321 (23.7)	1,237 (23.5)	2,998 (23.6)
25-34	1,113 (46.2)	2,749 (49.4)	2,664 (50.5)	6,315 (49.6)
35-49	678 (28.1)	1,452 (26.1)	1,304 (24.7)	3,297 (25.9)
**Mother’s education**				
No education	2,180 (90.5)	4,574 (82.2)	4,160 (78.9)	10,456 (82.2)
Primary and above	228 (9.5)	987 (17.8)	1,110 (21.1)	2,268 (17.8)
**Mother’s currently working**				
Yes	528 (21.9)	1,566 (28.2)	1,157 (21.9)	3,142 (24.7)
No	1,879 (78.1)	3,993 (71.8)	4,113 (78.1)	9,581 (75.3)
**Maternal BMI (kg/m**^**2**^)				
<18.5	532 (22.1)	1,267 (22.8)	1,148 (21.8)	2,860 (22.5)
18.5 to 24.9	1,797 (74.7)	4,164 (74.9)	3,913 (74.3)	9,457 (74.3)
25 +	78 (3.2)	131 (2.3)	205 (3.9)	402 (3.2)
**Maternal stature**				
Very short	76 (3.1)	153 (2.7)	141 (2.7)	365 (2.9)
Short	892 (37.0)	2,265 (40.7)	1,779 (33.7)	4,723 (37.1)
Normal	1,440 (59.8)	3,143 (56.5)	3,350 (63.5)	7,636 (60.0)
**Maternal anemia**				
Yes	745 (33.1)	1,213 (22.2)	1,919 (36.8)	3,694 (29.6)
No	1,506 (66.9)	4,249 (77.8)	3,297 (63.2)	8,765 (70.3)
**Place of delivery**				
Home	2,363 (98.5)	5,383 (97.1)	4,461 (84.7)	11,705 (92.1)
Health facility	35 (1.5)	163 (2.9)	803 (15.3)	1000 (7.9)
**Listening to radio**				
Yes	488 (20.3)	2,068 (37.2)	743 (14.1)	3,182 (25.0)
Not at all	1,919 (79.7)	3,491 (62.8)	4,527 (85.9)	9,539 (75.0)
**Watching television**				
Yes	47 (1.9)	1,339 (24.1)	354 (6.7)	1,724 (13.6)
Not at all	2,356 (98.1)	4,219 (75.9)	4,916 (93.3)	10,991 (86.4)
***Household factors***				
**Sex of the household head**				
Male	2,091 (86.8)	4,758 (85.5)	4,548 (86.3)	10,949 (86.1)
Female	316 (13.2)	804 (14.5)	722 (13.7)	1,775 (13.9)
**Household size**				
1-4	575 (23.9)	1,416 (25.5)	1,391 (26.4)	3,159 (24.8)
5+	1,832 (76.1)	4,145 (74.5)	3,879 (73.6)	9,564 (75.2)
**Sanitation facility**				
Improved	34 (1.4)	232 (4.3)	184 (3.5)	452 (3.6)
Unimproved	210 (8.9)	1,556 (28.7)	1,684 (32.3)	3,392 (27.1)
Open defecation	2,149 (89.8)	3,638 (67.0)	3,345 (64.2)	8,684 (69.3)
**Source of drinking water**				
Improved	387 (16.2)	1,175 (21.7)	1,408 (27.0)	4,164 (33.3)
Unimproved	2007 (83.8)	4,244 (78.3)	3,806 (73.0)	8,357 (66.7)
**Time to get a water source**				
On-premise	24 (1.0)	62 (1.1)	96 (1.8)	172 (1.3)
≤ 30 min	1,498 (62.2)	2,939 (52.8)	3,019 (57.3)	7,141 (56.1)
31-60 min	435 (18.1)	1,456 (26.2)	1,187 (22.5)	2,989 (23.5)
>60 min	450 (18.7)	1,104 (19.8)	967 (18.3)	2,422 (19.0)
**Child stool disposal**				
Safe	135 (5.6)	994 (18.0)	795 (15.1)	1,916 (15.1)
Unsafe	2,251 (94.4)	4,512 (82.0)	4,473 (84.9)	10,733 (84.9)
***Community-level characteristics***				
**Residence**				
Urban	11 (0.5)	48 (0.9)	88 (1.7)	145 (1.1)
Rural	2,397 (99.5)	5,513 (99.1)	5,183 (98.3)	12,579 (98.9)
**Region**				
Agrarian	1,310 (54.4)	3,270 (58.8)	2,858 (54.2)	7,158 (56.3)
Pastoralist	1,087 (45.1)	2,276 (40.9)	2,384 (45.2)	5,514 (43.3)
City administration	10 (0.4)	15 (0.3)	28 (0.5)	51 (0.4)

As shown in Table 1 (i.e., survey specific results): nearly one-fifth (19.6%) of the study participants had diarrhea in the past two weeks in 2005. However, only 11.2% of the study participants had diarrhea in the past two weeks months in 2016. Most of the study participants were from households practicing open defecation 89.8% in 2005, 67.0% in 2011, and 64.2% in 2016. Almost one-fifth of the mothers were underweighted throughout the survey years. The prevalence of anemia in mothers was 33.1%, 22.2%, and 36.8% in the years 2005, 2011, and 2016, respectively.

### Prevalence of stunting

**Fig 1:**
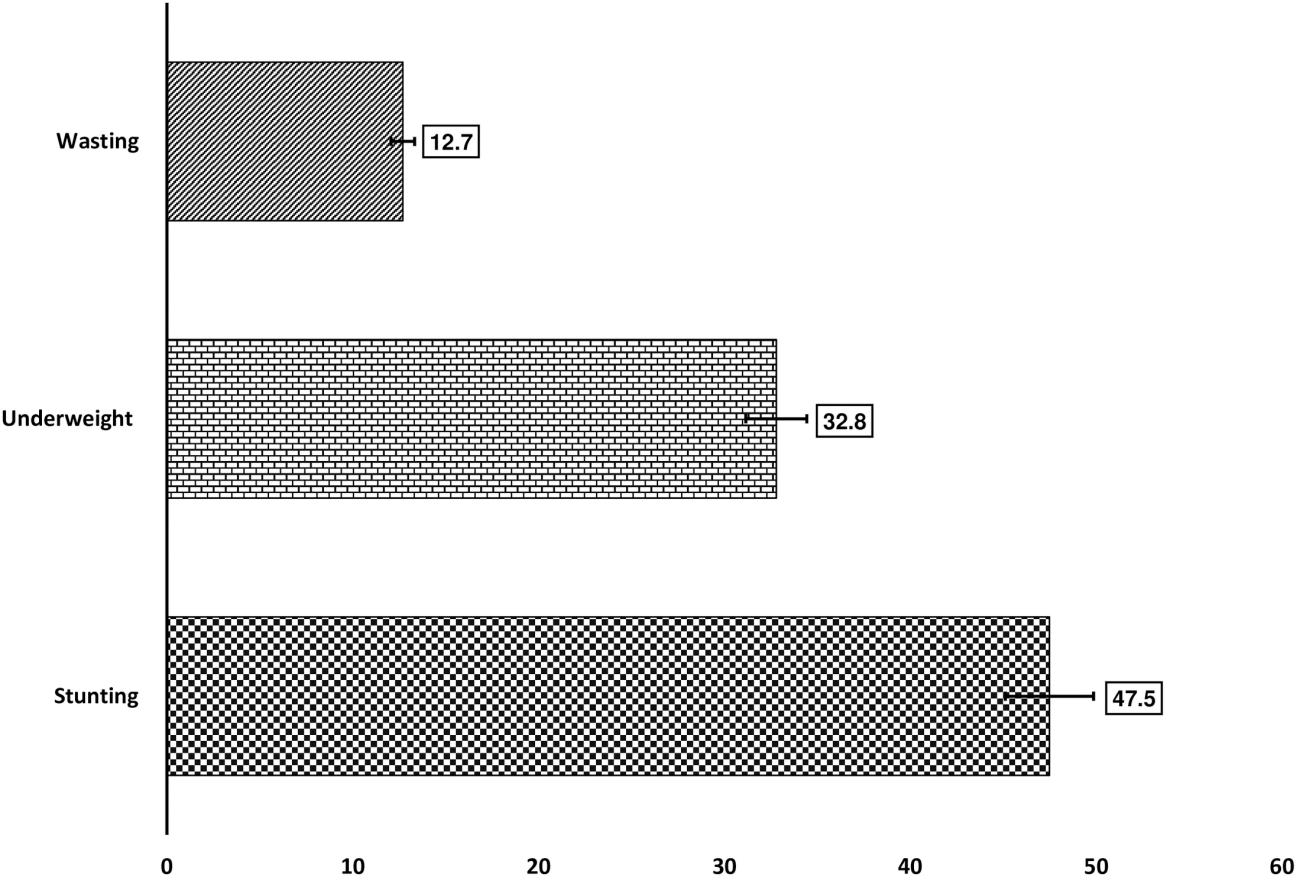
Childhood undernutrition among children aged 0-59 months in poor households in Ethiopia, EDHS (2005-2016).

Fig 1 represents the percentage of childhood undernutrition among poor households in Ethiopia between 2005 and 2016. It was found that about 47.5% (95% CI: 46.5–48.4) were stunted. The prevalence of stunting was drop from 53.9% (95% CI: 51.7–56.2) in the EDHS-2005 to 48.2% (95% CI: 46.7–49.7) in EDHS-2011 and 43.9% (95% CI: 42.5–45.4) in EDHS-2016.

Table 2 shows a comparison of the prevalence of stunting, wasting, and underweight in children with different characteristics. The prevalence of stunting significantly differed with the sex of the child, child’s age, perceived size of the child at birth, breastfeeding status, mother’s education, maternal BMI, sanitation facility, and place of residence (p-value<0.05).

**Table 2:** Prevalence of stunting, wasting and underweight among children 0-59 months in poor households with different characteristics for the survey year 2005, 2011 and 2016.

Variables	Undernutrition (EDHS 2005–2016)		
	Prevalence of stunting, 95%CI	Prevalence of wasting, 95%CI	Prevalence underweight, 95%CI
***Undernutrition status***	*47.5% (46.5-48.4)*	*12.7% (12.1-13.3).*	*32.8% (31.9-33.7)*
**Child factors**			
**Sex**			
Male	48.9 (47.6-50.2)	14.2 (13.3-15.2)	34.4 (33.1-35.6)
Female	46.0 (44.7-47.4)	11.1 (10.3-11.9)	31.2 (30.0-32.5)
**Age (months)**			
**< 6**	14.2 (12.2-16.4)	14.8 (12.8-17.1)	9.8 (8.2-11.8)
6-11	26.8 (24.3-29.4)	18.8 (16.7-21.2)	25.1 (22.6-27.7)
12-23	49.5 (47.3-51.7)	18.5 (16.8-20.2)	35.1 (33.1-37.2)
24-35	57.5 (55.4-59.7)	10.6 (9.3-12.0)	36.6 (34.5-38.7)
36-59	55.1 (53.7-56.6)	8.9 (8.1-9.8)	37.5 (36.1-38.9)
**Size of the child at birth**			
Larger	43.8 (42.1-45.5)	10.7 (9.7-11.8)	27.2 (25.6-28.7)
Average	47.7 (46.1-49.2)	11.2 (10.4-12.3)	31.9 (30.6-33.4)
Small	50.9 (49.1-52.6)	16.5 (15.3-17.8)	39.7 (38.1-41.4)
**Birth order**			
Firstborn	48.9 (46.5-51.3)	12.4 (10.9-14.1)	32.0 (29.8-34.3)
2-4	47.3 (45.8-48.7)	12.9 (11.9-13.9)	34.1 (32.8-35.5)
5+	47.1 (45.6-48.6)	12.6 (11.6-13.6)	31.8 (30.5-33.2)
**Full vaccination**			
Yes	51.2 (48.7-53.7)	10.7 (9.2-12.4)	32.4 (30.1-34.8)
No	45.5 (44.4-46.7)	14.1 (13.3-14.9)	32.7 (31.6-33.7)
**Vitamin A last 6 months**			
Yes	51.0 (49.5-52.4)	12.1 (11.2-13.1)	32.8 (31.5-34.2)
No	44.6 (43.3-45.8)	13.2 (12.4-14.1)	32.9 (31.7-34.1)
**Currently breastfeeding**			
Yes	46.2 (45.1-47.3)	13.5 (12.8-14.3)	32.5 (31.6-33.6)
No	51.2 (49.3-53.1)	10.3 (9.2-11.5)	33.7 (31.9-35.5)
**Early initiation of breastfeeding**			
Yes	44.4 (42.9-45.9)	14.8 (13.8-15.9)	30.5 (29.2-31.9)
No	46.8 (44.9-48.7)	14.4 (13.1-15.8)	33.3 (31.5-35.1)
**Birth interval**			
Less than 33 months	47.3 (46.1-48.4)	13.0 (12.3-13.8)	32.7 (31.6-33.7)
≥ 33 months	47.9 (46.2-49.6)	11.9 (10.9-13.1)	33.2 (31.6-34.8)
**Diarrhea**			
Yes	50.5 (47.9-53.0)	17.6 (15.7-19.6)	40.0 (37.6-42.5)
No	47.0 (46.0-48.0)	11.9 (11.2-12.5)	31.6 (30.7-32.6)
**Fever**			
Yes	46.9 (44.6-49.3)	18.8 (17.1-20.7)	38.2 (36.0-40.5)
No	47.6 (46.5-48.6)	11.5 (10.8-12.2)	31.8 (30.8-32.8)
**Cough**			
Yes	45.4 (43.2-47.5)	15.2 (13.8-16.8)	34.9 (32.9-36.9)
No	47.9 (46.9-49.0)	12.1 (11.4-12.8)	32.4 (31.3-33.3)
**Parental factors**			
**Mother’s age**			
15-17	43.2 (33.2-53.6)	14.3 (8.3-23.4)	29.7 (21.3-39.7)
18-24	43.2 (33.2-53.7)	14.1 (12.7-15.5)	30.8 (29.1-32.7)
25-34	47.9 (46.6-49.3)	12.3 (11.5-13.2)	34.4 (33.1-35.6)
35-49	47.3 (45.4-49.1)	12.1 (10.9-13.3)	31.7 (30.0-33.4)
**Mother’s education**			
No education	47.8 (46.8-48.9)	13.2 (12.5-13.9)	34.3 (33.3-35.3)
Primary and above	45.8 (43.6-48.0)	10.3 (9.0-11.7)	26.3 (24.4-28.2)
**Mother’s currently working**			
Yes	49.1 (47.2-51.0)	13.0 (11.8-14.4)	31.8 (30.1-33.6)
No	46.9 (45.8-48.0)	12.6 (11.9-13.3)	33.2 (32.2-34.2)
**Maternal BMI (kg/m**^**2**^)			
<18.5	49.1 (47.1-51.0)	16.9 (15.5-18.4)	39.8 (37.9-41.8)
18.5 to 24.9	47.1 (46.0-48.2)	11.5 (10.8-12.2)	31.1 (30.1-32.2)
25 +	45.1 (39.8-50.6)	10.5 (7.6-14.4)	22.2 (18.0-27.1)
**Maternal stature**			
Very short	59.6 (54.0-64.9)	14.8 (11.3-19.3)	45.4 (39.9-51.0)
Short	55.4 (53.8-56.9)	13.6 (12.5-14.7)	37.8 (36.3-39.3)
Normal	42.0 (40.8-43.2)	12.1 (11.3-12.9)	29.2 (28.1-30.3)
**Maternal anemia**			
Yes	45.5 (43.7-47.2)	12.9 (11.8-14.2)	32.9 (31.2-34.6)
No	48.2 (47.1-49.4)	12.6 (11.9-13.4)	32.8 (31.7-33.8)
**Place of delivery**			
Home	47.8 (46.9-48.8)	12.9 (12.3-13.6)	33.4 (32.5-34.4)
Health facility	42.9 (39.7-46.3)	9.9 (8.1-12.1)	26.1 (23.3-29.1)
**Listening to radio**			
Yes	46.9 (44.9-48.8)	11.4 (10.3-12.7)	32.1 (30.4-33.9)
Not at all	47.6 (46.5-48.7)	13.1 (12.4-13.8)	33.1 (32.0-34.1)
**Watching television**			
Yes	47.0 (44.5-49.5)	9.9 (8.5-11.6)	32.5 (30.1-34.9)
Not at all	47.5 (46.5-48.6)	13.1 (12.4-13.8)	32.9 (31.9-33.8)
**Household factors**			
**Sex of the household head**			
Male	47.5 (46.5-48.5)	12.7 (12.1-13.4)	32.7 (31.8-33.7)
Female	47.4 (44.8-49.9)	12.5 (10.9-14.2)	33.6 (31.3-36.0)
**Household size**			
1-4	48.2 (46.2-50.2)	13.2 (11.9-14.5)	33.4 (31.6-35.2)
5+	47.2 (46.2-48.3)	12.5 (11.8-13.3)	32.7 (31.7-33.7)
**Sanitation facility**			
Improved	41.6 (36.8-46.7)	11.2 (8.4-14.8)	28.7 (24.4-33.5)
Unimproved	46.5 (44.7-48.3)	11.1 (10.0-12.3)	29.7 (28.1-31.4)
Open defecation	48.0 (46.9-49.2)	13.4 (12.6-14.2)	34.3 (33.2-35.3)
**Source of drinking water**			
Improved	49.0 (47.4-50.7)	14.5 (13.4-15.8)	35.7 (34.1-37.3)
Unimproved	46.6 (45.4-47.7)	11.8 (11.1-12.6)	31.4 (30.4-32.5)
**Time to get a water source**			
On-premise	46.2 (37.8-54.8)	13.8 (8.9-20.9)	34.1 (26.6-42.5)
≤ 30 min	47.5 (46.3-48.8)	12.2 (11.4-13.1)	32.0 (30.8-33.2)
31-60 min	47.9 (45.9-49.8)	11.8 (10.6-13.2)	32.5 (30.7-34.3)
>60 min	46.8 (44.6-48.9)	15.1 (13.6-16.8)	35.7 (33.7-37.8)
**Child stool disposal**			
Safe	45.4 (43.1-47.8)	11.8 (10.3-13.4)	30.7 (28.5-32.9)
Unsafe	47.8 (46.8-48.8)	12.8 (12.1-13.5)	33.3 (32.3-34.2)
**Community-level characteristics**			
**Residence**			
Urban	51.7 (43.1-60.4)	13.3 (8.4-20.4)	21.3 (15.1-29.3)
Rural	47.4 (46.5-48.4)	12.7 (12.1-13.3)	32.9 (32.1-33.8)
**Region**			
Agrarian	49.8 (48.6-51.1)	12.5 (11.7-13.4)	34.7 (33.5-35.9)
Pastoralist	44.4 (43.0-45.8)	12.9 (12.0-13.9)	30.4 (29.1-31.8)
City administration	48.5 (34.1-63.1)	12.7 (5.6-26.2)	36.5 (23.8-51.5)

### Prevalence of wasting

The overall prevalence of wasting was 12.7% (95% CI: 12.1–13.3). The prevalence of wasting dropped from 15.3% (95% CI: 13.7–16.9) from the EDHS-2005 to 12.4% (95% CI: 11.5–13.4) in EDHS-2011 and 11.9% (95% CI: 10.9–12.8) in EDHS-2016. The prevalence of wasting significantly differed with the sex of the child, age, vaccination status, birth order, perceived size of the child at birth, mother’s education, sanitation facility, and residence (p-value<0.05) (Table 2).

### Prevalence of underweight

It was found that about 32.8% (95% CI: 31.9–33.7) were underweight. The prevalence of underweight fell from 37.7% (95% CI: 35.5–39.9) from the EDHS-2005 to 34.4% (95% CI: 33.1–35.8) in EDHS-2011 and 29.2% (95% CI: 27.9–30.5) in EDHS-2016. The prevalence of underweight significantly differed with the sex of the child, age, perceived size of the child at birth, mother’s education, maternal BMI (kg/m^2^), maternal stature, maternal anemia, sanitation facility, and residence (p-value<0.05) (Table 2).

S1–S3 Files shows a comparison of the prevalence of stunting, wasting, and underweight in children with different characteristics for the survey year 2005, 2011, and 2016, respectively.

#### Factors associated with stunting among children 0–59 months in poor households.

Table 3 shows the results from a multilevel bivariable analysis of factors associated with stunting, wasting, and underweight status of children.

**Table 3:** Multilevel bivariable binary logistic regression analysis of factors associated with stunting, wasting and underweight in children aged 0-59 months in Ethiopia, EDHS (2005-2016).

	Stunting		Wasting		Underweight	
	Crude OR (95%CI)	p-values	Crude OR (95%CI)	p-values	Crude OR (95%CI)	p-values
**Variables**						
***Child factors***						
**Sex**						
Male	1.13 (1.04-1.22)	0.001	1.32 (1.19-1.47)	p < 0.001	1.18 (1.09-1.28)	p < 0.001
Female	Ref.		Ref.		Ref.	
**Age (months)**						
**< 6**	0.12 (0.10-0.15)	p < 0.001	1.77 (1.47-2.13)	p < 0.001	0.20 (0.17-0.25)	p < 0.001
6-11	0.25 (0.22-0.29)	p < 0.001	2.29 (1.93-2.72)	p < 0.001	0.53 (0.45-0.61)	p < 0.001
12-23	0.84 (0.76-0.94)	0.002	2.16 (1.87-2.49)	p < 0.001	0.94 (0.84-1.05)	0.312
24-35	1.31 (1.18-1.46)	p < 0.001	1.08 (0.92-1.27)	0.340	1.10 (0.99-1.23)	0.061
36-59	Ref.		Ref.		Ref.	
**Size of the child at birth**						
Larger	Ref.		Ref.		Ref.	
Average	1.19 (1.08-1.32)	p < 0.001	1.09 (0.95-1.27)	0.192	1.26 (1.14-1.40)	p < 0.001
Small	1.27 (1.14-1.40)	p < 0.001	1.72 (1.49-1.98)	p < 0.001	1.68 (1.51-1.87)	p < 0.001
**Birth order**						
Firstborn	Ref.		Ref.		Ref.	
2-4	1.01 (0.90-1.14)	0.795	1.04 (0.88-1.22)	0.628	1.15 (1.02-1.30)	0.018
5+	1.06 (0.95-1.19)	0.302	1.17 (1.01-1.38)	0.048	1.20 (1.06-1.35)	0.003
**Full vaccination**						
Yes	Ref.		Ref.		Ref.	
No	0.68 (0.61-0.77)	p < 0.001	1.40 (1.18-1.66)	p < 0.001	0.91 (0.80-1.02)	0.119
**Vitamin A last 6 months**						
Yes	Ref.		Ref.		Ref.	
No	0.75 (0.69-0.81)	p < 0.001	1.19 (1.06-1.33)	0.002	0.96 (0.88-1.04)	0.353
**Currently breastfeeding**						
Yes	Ref.		Ref.		Ref.	
No	1.35 (1.24-1.47)	p < 0.001	0.78 (0.69-0.88)	p < 0.001	1.17 (1.07-1.28)	p < 0.001
**Early initiation of breastfeeding**						
Yes	Ref.		Ref.		Ref.	
No	1.03 (0.93-1.14)	0.565	0.97 (0.85-1.11)	0.691	1.08 (0.97-1.19)	0.156
**Birth interval**						
Less than 33 months	0.91 (0.83-0.98)	0.024	1.07 (0.96-1.21)	0.220	0.93 (0.85-1.01)	0.105
≥ 33 months	Ref.		Ref.		Ref.	
**Diarrhea**						
Yes	1.19 (1.06-1.33)	0.002	1.56 (1.35-1.79)	p < 0.001	1.43 (1.27-1.59)	p < 0.001
No	Ref.		Ref.		Ref.	
**Fever**						
Yes	1.07 (0.97-1.19)	0.176	1.54 (1.35-1.75)	p < 0.001	1.29 (1.17-1.44)	p < 0.001
No	Ref.		Ref.		Ref.	
**Cough**						
Yes	1.04 (0.94-1.15)	0.407	1.19 (1.04-1.36)	0.011	1.13 (1.02-1.25)	0.021
No	Ref.		Ref.		Ref.	
***Parental factors***						
**Mother’s age**						
15-17	0.45 (0.28-0.72)	0.001	1.41 (0.82-2.41)	0.212	0.65 (0.41-1.05)	0.078
18-24	0.85 (0.76-0.95)	0.006	1.11 (0.95-1.29)	0.166	0.86 (0.77-0.97)	0.018
25-34	0.94 (0.86-1.03)	0.244	1.01 (0.89-1.16)	0.810	1.03 (0.94-1.14)	0.487
35-49	Ref.		Ref.		Ref.	
**Mother’s education**						
No education	1.27 (1.15-1.41)	p < 0.001	1.23 (1.06-1.43)	0.005	1.54 (1.38-1.72)	p < 0.001
Primary and above	Ref.		Ref.		Ref.	
**Mother’s currently working**						
Yes	1.13 (1.02-1.24)	0.012	0.87 (0.76-0.99)	0.046	1.01 (0.90-1.10)	0.961
No	Ref.		Ref.		Ref.	
**Maternal BMI (kg/m**^**2**^)						
<18.5	Ref.		Ref.		Ref.	
18.5 to 24.9	0.93 (0.85-1.01)	0.107	0.56 (0.50-0.63)	p < 0.001	0.66 (0.60-0.72)	p < 0.001
25 +	0.72 (0.57-0.92)	0.009	0.48 (0.33-0.68)	p < 0.001	0.39 (0.30-0.52)	p < 0.001
**Maternal stature**						
Very short	2.08 (1.58-2.73)	p < 0.001	0.93 (0.64-1.36)	0.719	1.87 (1.43-2.43)	p < 0.001
Short	1.73 (1.59-1.88)	p < 0.001	0.97 (0.86-1.09)	0.678	1.48 (1.36-1.62)	p < 0.001
Normal	Ref.		Ref.		Ref.	
**Maternal anemia**						
Yes	0.92 (0.84-1.01)	0.055	1.08 (0.97-1.22)	0.162	1.08 (0.98-1.17)	0.087
No	Ref.		Ref.		Ref.	
**Place of delivery**						
Home	1.34 (1.17-1.52)	p < 0.001	1.16 (0.96-1.40)	0.117	1.42 (1.23-1.63)	p < 0.001
Health facility	Ref.		Ref.		Ref.	
**Listening to radio**						
Yes	Ref.		Ref.		Ref.	
Not at all	0.94 (0.85-1.03)	0.202	1.27 (1.11-1.47)	0.001	1.03 (0.93-1.15)	0.487
**Watching television**						
Yes	Ref.		Ref.		Ref.	
Not at all	0.96 (0.84-1.10)	0.577	1.61 (1.30-2.00)	p < 0.001	1.04 (0.90-1.19)	0.609
***Household factors***						
**Sex of the household head**						
Male	Ref.		Ref.		Ref.	
Female	0.95 (0.86-1.05)	0.367	1.19 (1.05-1.36)	0.007	1.04 (0.93-1.15)	0.483
**Household size**						
1-4	0.95 (0.86-1.04)	0.282	0.88 (0.78-1.01)	0.073	0.91 (0.83-1.01)	0.059
5+	Ref.		Ref.		Ref.	
**Sanitation facility**						
Improved	Ref.		Ref.		Ref.	
Unimproved	1.39 (1.10-1.75)	0.005	0.89 (0.65-1.22)	0.483	1.23 (0.96-1.57)	0.091
Open defecation	1.38 (1.11-1.71)	0.004	1.17 (0.87-1.57)	0.292	1.37 (1.08-1.72)	0.008
**Source of drinking water**						
Improved	Ref.		Ref.		Ref.	
Unimproved	0.85 (0.78-0.94)	0.001	0.84 (0.74-0.94)	0.004	0.84 (0.76-0.92)	p < 0.001
**Time to get a water source**						
On-premise	Ref.		Ref.		Ref.	
≤ 30 min	1.11 (0.82-1.49)	0.491	0.82 (0.55-1.21)	0.318	0.81 (0.59-1.09)	0.165
31-60 min	1.18 (0.86-1.59)	0.291	0.89 (0.60-1.34)	0.605	0.85 (0.63-1.16)	0.329
>60 min	1.10 (0.81-1.49)	0.517	1.12 (0.75-1.67)	0.555	0.95 (0.70-1.29)	0.747
**Child stool disposal**						
Safe	Ref.		Ref.		Ref.	
Unsafe	1.16 (1.03-1.30)	0.013	1.17 (0.98-1.38)	0.074	1.13 (1.01-1.28)	0.049
***Community-level characteristics***						
**Residence**						
Urban	0.65 (0.48-0.88)	0.005	1.23 (0.86-1.77)	0.257	0.68 (0.49-0.93)	0.017
Rural	Ref.		Ref.		Ref.	
**Region**						
Agrarian	0.87 (0.72-1.05)	0.155	1.05 (0.81-1.36)	0.682	0.87 (0.72-1.06)	0.182
Pastoralist	0.92 (0.76-1.13)	0.475	1.23 (0.94-1.62)	0.120	0.98 (0.81-1.21)	0.910
City administration	Ref.		Ref.		Ref.	
**Survey years**						
EDHS-2005	1.55 (1.37-1.75)	p < 0.001	1.13 (0.96-1.32)	0.135	1.32 (1.17-1.50)	p < 0.001
EDHS-2011	1.28 (1.17-1.41)	p < 0.001	0.94 (0.83-1.06)	0.318	1.24 (1.13-1.36)	p < 0.001
EDHS-2016	Ref.		Ref.		Ref.	

Table 4 presents the multilevel multivariable logistic regression analysis results of factors associated with stunting among children aged 0–59 months. The odds of children suffering from stunting were higher for male children (AOR: 1.17, 95%CI: 1.06–1.29) than for females. Children in the age group of 0–5 months (AOR: 0.11, 95%CI: 0.09–0.14), 6–11 months (AOR: 0.21, 95%CI: 0.17–0.25), and 12–23 months (AOR: 0.74, 95%CI: 0.64–0.85) had lower odds of stunting compared with those 36–59 months of age, respectively. However, children in the age group of 24–35 months (AOR: 1.27, 95%CI: 1.11–1.46) had higher odds of stunting compared with those 36–59 months of age. Children who were perceived by their mothers to be smaller (AOR: 1.51, 95%CI: 1.33–1.71) and average size (AOR: 1.30, 95%CI: 1.15–1.47) than normal at birth reported higher odds of stunting. The odds of stunting among children with diarrhea were higher compared with those of children without diarrhea (AOR: 1.21, 95%CI: 1.05–1.40). The odds of stunting among children born to mothers with no education (AOR: 1.17, 95%CI: 1.02–1.33) were higher compared with those reporting primary and above education. Compared to the children of tall mothers (height ≥155 cm), the odds of stunting significantly increased about 2.88 times among the children of mothers with very short height (height<145 cm) (AOR: 2.88, 95%CI: 2.04–4.06). The odds of stunting were 1.96 times higher for the children of the shortest mothers (145–155 cm) (AOR: 1.96, 95%CI: 1.76–2.18) compared to the children of the tall mothers. The odds of children suffering from stunting were higher for children from households with unimproved toilet facilities (AOR: 1.43; 95% CI: 1.06–1.91) and open defecation (AOR: 1.37, 95%CI: 1.03–1.82) compared with children from households with improved sanitation facility.

**Table 4:** Multilevel multivariable binary logistic regression analysis of factors associated with stunting in children aged 0-59 months in Ethiopia, EDHS (2005-2016).

	Stunting %, (95%CI)	Model 1	Model 2	Model 3	Model 4
			AOR (95%CI)	AOR (95%CI)	AOR (95%CI)
**Variables**					
***Child factors***					
**Sex**					
Male	48.9 (47.6-50.2)		1.17 (1.06-1.28) ^*^		1.17 (1.06-1.29) ^*^
Female	46.0 (44.7-47.4)		Ref.		Ref.
**Age (months)**					
**< 6**	14.2 (12.2-16.4)		0.10 (0.08-0.13) ^**^		0.11 (0.09-0.14) ^**^
6-11	26.8 (24.3-29.4)		0.19 (0.16-0.23) ^**^		0.21 (0.17-0.25) ^**^
12-23	49.5 (47.3-51.7)		0.68 (0.60-0.78) ^**^		0.74 (0.64-0.85) ^**^
24-35	57.5 (55.4-59.7)		1.17 (1.03-1.33) ^*^		1.27 (1.11-1.46) ^*^
36-59	55.1 (53.7-56.6)		Ref.		Ref.
**Size of the child at birth**					
Larger	43.8 (42.1-45.5)		Ref.		Ref.
Average	47.7 (46.1-49.2)		1.29 (1.15-1.46) ^**^		1.30 (1.15-1.47) ^**^
Small	50.9 (49.1-52.6)		1.50 (1.32-1.71) ^**^		1.51 (1.33-1.71) ^**^
**Full vaccination**					
Yes	51.2 (48.7-53.7)		Ref.		Ref.
No	45.5 (44.4-46.7)		0.96 (0.84-1.10)		0.95 (0.83-1.09)
**Vitamin A last 6 months**					
Yes	51.0 (49.5-52.4)		Ref.		Ref.
No	44.6 (43.3-45.8)		0.89 (0.79-0.97) ^*^		0.88 (0.79-0.98) ^*^
**Currently breastfeeding**					
Yes	46.2 (45.1-47.3)		Ref.		Ref.
No	51.2 (49.3-53.1)		0.93 (0.83-1.04)		0.94 (0.84-1.06)
**Birth interval**					
Less than 33 months	47.3 (46.1-48.4)		1.02 (0.81-1.28)		1.03 (0.82-1.30)
≥ 33 months	47.9 (46.2-49.6)		Ref.		Ref.
**Diarrhea**					
Yes	50.5 (47.9-53.0)		1.24 (1.08-1.43) ^*^		1.21 (1.05-1.40) ^*^
No	47.0 (46.0-48.0)		Ref.		Ref.
**Fever**					
Yes	46.9 (44.6-49.3)		1.04 (0.91-1.19)		1.04 (0.91-1.19)
No	47.6 (46.5-48.6)		Ref.		Ref.
***Parental factors***					
**Mother’s age**					
15-17	43.2 (33.2-53.6)		0.73 (0.39-1.36)		0.72 (0.38-1.35)
18-24	43.2 (33.2-53.7)		0.97 (0.74-1.28)		0.96 (0.73-1.25)
25-34	47.9 (46.6-49.3)		1.01 (0.80-1.28)		1.01 (0.79-1.27)
35-49	47.3 (45.4-49.1)		Ref.		Ref.
**Mother’s education**					
No education	47.8 (46.8-48.9)		1.19 (1.04-1.36) ^*^		1.17 (1.02-1.33) ^*^
Primary and above	45.8 (43.6-48.0)		Ref.		Ref.
**Mother’s currently working**					
Yes	49.1 (47.2-51.0)		0.99 (0.89-1.12)		1.01 (0.89-1.12)
No	46.9 (45.8-48.0)		Ref.		Ref.
**Maternal BMI (kg/m**^**2**^)					
<18.5	49.1 (47.1-51.0)		Ref.		Ref.
18.5 to 24.9	47.1 (46.0-48.2)		0.90 (0.81-1.01)		0.89 (0.80-1.01)
25 +	45.1 (39.8-50.6)		0.72 (0.53-0.98) ^*^		0.74 (0.54-1.01)
**Maternal stature**					
Very short	59.6 (54.0-64.9)		2.88 (2.04-4.06) ^**^		2.88 (2.04-4.06) ^*^
Short	55.4 (53.8-56.9)		1.97 (1.77-2.18) ^**^		1.96 (1.76-2.18) ^*^
Normal	42.0 (40.8-43.2)		Ref.		Ref.
**Maternal anemia**					
Yes	45.5 (43.7-47.2)		0.94 (0.85-1.04)		0.95 (0.85-1.06)
No	48.2 (47.1-49.4)		Ref.		Ref.
**Place of delivery**					
Home	47.8 (46.9-48.8)		1.08 (0.91-1.28)		0.99 (0.83-1.19)
Health facility	42.9 (39.7-46.3)		Ref.		Ref.
**Listening to radio**					
Yes	46.9 (44.9-48.8)		Ref.		Ref.
Not at all	47.6 (46.5-48.7)		0.95 (0.84-1.07)		0.96 (0.85-1.09)
**Sanitation facility**					
Improved	41.6 (36.8-46.7)		Ref.		Ref.
Unimproved	46.5 (44.7-48.3)		1.45 (1.08-1.94) ^*^		1.43 (1.06-1.91) ^*^
Open defecation	48.0 (46.9-49.2)		1.44 (1.09-1.91) ^*^		1.37 (1.03-1.82) ^*^
**Source of drinking water**					
Improved	49.0 (47.4-50.7)		Ref.		Ref.
Unimproved	46.6 (45.4-47.7)		0.83 (0.74-0.93) ^*^		0.91 (0.80-1.04)
**Child stool disposal**					
Safe	45.4 (43.1-47.8)		Ref.		Ref.
Unsafe	47.8 (46.8-48.8)		1.15 (0.98-1.35)		1.14 (0.97-1.33)
***Community-level characteristics***					
**Residence**					
Urban	51.7 (43.1-60.4)			0.72 (0.52-0.95) ^*^	0.78 (0.53-1.15)
Rural	47.4 (46.5-48.4)			Ref.	Ref.
**Region**					
Agrarian	49.8 (48.6-51.1)			0.86 (0.71-1.04)	0.85 (0.67-1.07)
Pastoralist	44.4 (43.0-45.8)			0.90 (0.74-1.09)	0.86 (0.67-1.10)
City administration	48.5 (34.1-63.1)			Ref.	Ref.
**Survey years**					
EDHS-2005				1.54 (1.36-1.73) ^**^	1.43 (1.19-1.72) ^**^
EDHS-2011				1.28 (1.17-1.40) ^**^	1.19 (1.04-1.36) ^*^
EDHS-2016				Ref.	Ref.
**Random effects**					
**Variance (SD)**		0.1557 (0.0009)	0.1519 (0.0016)	0.1500 (0.0009)	0.1445 (0.0016)
**ICC (%)**		4.52	4.41	4.36	4.21
**MOR**		1.45	1.44	1.44	1.43
**PCV (%)**		Ref.	2.44	3.66	7.19
**Model fit statistics**					
**AIC**		15176.02	10243.75	15118.42	10235.38
**BIC**		15190.65	10461.87	15169.61	10488.68
**Log- likelihood (LL)**		-7586.01	-5090.87	-7552.21	-5081.69
**Deviance**		15,172.02	10,181.74	15,104.41	10,163.37

*p-value < 0.05,

**p-value< 0.001; SE: Standard error; ICC: Intra-class Correction Coefficient; MOR: Median Odds Ratio; PCV: Proportional Change in Variance; AIC: Akaike’s Information Criterion; BIC: Bayesian Information Criteria; LL: Log-likelihood.

#### Factors associated with wasting among children 0–59 months in poor households.

Table 5 presents the multilevel multivariable logistic regression analysis results of factors associated with wasting. The odds of children suffering from wasting were higher for male children (AOR: 1.36, 95%CI: 1.20–1.54) than for females. Children in the age group of 0–5 months had 1.81 (AOR: 1.81, 95%CI: 1.43–2.31), 6–11 months had 2.38 (AOR: 2.38, 95%CI: 1.90–2.98), and 12–23 months had 2.19 times (AOR: 2.19, 95%CI: 1.80–2.66) higher odds of wasting compared with those 36–59 months of age, respectively. The odds of wasting were higher for children who were perceived by their mothers to be smaller (AOR: 1.56, 95%CI: 1.33–1.84) than normal at birth. The odds of wasting among children having diarrhea (AOR: 1.20, 95%CI: 1.01–1.42) and fever (AOR: 1.38, 95%CI: 1.15–1.66) were higher compared with their counterparts. Compared with children with underweight mothers those with normal BMI (AOR: 0.61, 95%CI: 0.53–0.69) and overweight or obese mothers (AOR: 0.52, 95%CI: 0.34–0.79) were at lower odds of wasting. Children in female-headed households (AOR: 1.18, 95%CI: 1.01–1.37), those not exposed to television (AOR: 1.46, 95%CI: 1.12–1.89), and those from urban households (AOR: 1.67, 95%CI: 1.09–2.54) had higher odds of wasting. On the other hand, children from households with 1–4 family sizes (AOR: 0.81, 95%CI: 0.68–0.97) and those from households with unimproved sources of drinking water (AOR: 0.82, 95%CI: 0.69–0.96) had lower odds of wasting compared to their counterparts (Table 5).

**Table 5:** Multilevel multivariable binary logistic regression analysis of factors associated with wasting in children aged 0-59 months in Ethiopia, EDHS (2005-2016).

	Wasting % (95%CI)	Model 1	Model 2	Model 3	Model 4
			AOR (95%CI)	AOR (95%CI)	AOR (95%CI)
**Variables**					
***Child factors***					
**Sex**					
Male	14.2 (13.3-15.2)		1.37 (1.21-1.55) ^**^		1.36 (1.20-1.54) ^**^
Female	11.1 (10.3-11.9)		Ref.		Ref.
**Age (months)**					
**< 6**	14.8 (12.8-17.1)		1.84 (1.45-2.31) ^**^		1.81 (1.43-2.31) ^**^
6-11	18.8 (16.7-21.2)		2.39 (1.92-2.97) ^**^		2.38 (1.90-2.98) ^**^
12-23	18.5 (16.8-20.2)		2.21 (1.84-2.67) ^**^		2.19 (1.80-2.66) ^**^
24-35	10.6 (9.3-12.0)		1.09 (0.90-1.33)		1.08 (0.88-1.33)
36-59	8.9 (8.1-9.8)		Ref.		
**Size of the child at birth**					
Larger	10.7 (9.7-11.8)		Ref.		Ref.
Average	11.2 (10.4-12.3)		1.05 (0.89-1.23)		1.05 (0.89-1.24)
Small	16.5 (15.3-17.8)		1.57 (1.33-1.84) ^**^		1.56 (1.33-1.84) ^**^
**Birth order**					
Firstborn	12.4 (10.9-14.1)		Ref.		Ref.
2-4	12.9 (11.9-13.9)		1.01 (0.82-1.24)		1.01 (0.81-1.24)
5+	12.6 (11.6-13.6)		1.22 (0.93-1.59)		1.21 (0.93-1.58)
**Full vaccination**					
Yes	10.7 (9.2-12.4)		Ref.		Ref.
No	14.1 (13.3-14.9)		1.16 (0.96-1.40)		1.12 (0.92-1.36)
**Vitamin A last 6 months**					
Yes	12.1 (11.2-13.1)		Ref.		Ref.
No	13.2 (12.4-14.1)		1.09 (0.96-1.25)		1.09 (0.95-1.24)
**Currently breastfeeding**					
Yes	13.5 (12.8-14.3)		Ref.		Ref.
No	10.3 (9.2-11.5)		1.11 (0.94-1.31)		1.10 (0.93-1.29)
**Birth interval**					
Less than 33 months	13.0 (12.3-13.8)		1.28 (0.94-1.75)		1.26 (0.92-1.72)
≥ 33 months	11.9 (10.9-13.1)		Ref.		Ref.
**Diarrhea**					
Yes	17.6 (15.7-19.6)		1.19 (1.01-1.41) ^*^		1.20 (1.01-1.42) ^*^
No	11.9 (11.2-12.5)		Ref.		Ref.
**Fever**					
Yes	18.8 (17.1-20.7)		1.38 (1.15-1.66) ^*^		1.38 (1.15-1.66) ^*^
No	11.5 (10.8-12.2)		Ref.		Ref.
**Cough**					
Yes	15.2 (13.8-16.8)		0.87 (0.72-1.04)		0.87 (0.72-1.04)
No	12.1 (11.4-12.8)		Ref.		Re.
***Parental factors***					
**Mother’s age**					
15-17	14.3 (8.3-23.4)		1.05 (0.52-2.12)		1.06 (0.52-2.15)
18-24	14.1 (12.7-15.5)		1.03 (0.71-1.49)		1.03 (0.71-1.49)
25-34	12.3 (11.5-13.2)		0.90 (0.66-1.23)		0.91 (0.66-1.25)
35-49	12.1 (10.9-13.3)		Ref.		Ref.
**Mother’s education**					
No education	13.2 (12.5-13.9)		1.17 (0.98-1.39)		1.17 (0.98-1.39)
Primary and above	10.3 (9.0-11.7)		Ref.		Ref.
**Mother’s currently working**					
Yes	13.0 (11.8-14.4)		0.92 (0.79-1.08)		0.93 (0.79-1.08)
No	12.6 (11.9-13.3)		Ref.		Ref.
**Maternal BMI (kg/m**^**2**^)					
<18.5	16.9 (15.5-18.4)		Ref.		Ref.
18.5 to 24.9	11.5 (10.8-12.2)		0.60 (0.53-0.69) ^**^		0.61 (0.53-0.69) ^**^
25 +	10.5 (7.6-14.4)		0.53 (0.35-0.81) ^*^		0.52 (0.34-0.79) ^*^
**Maternal anemia**					
Yes	12.9 (11.8-14.2)		1.01 (0.88-1.15)		1.01 (0.88-1.15)
No	12.6 (11.9-13.4)		Ref.		Ref.
**Place of delivery**					
Home	12.9 (12.3-13.6)		1.16 (0.93-1.44)		1.17 (0.93-1.47)
Health facility	9.9 (8.1-12.1)		Ref.		Ref.
**Listening to radio**					
Yes	11.4 (10.3-12.7)		Ref.		Ref.
Not at all	13.1 (12.4-13.8)		1.19 (1.01-1.41) ^*^		1.22 (1.03-1.44) ^*^
**Watching television**					
Yes	9.9 (8.5-11.6)		Ref.		Ref.
Not at all	13.1 (12.4-13.8)		1.46 (1.13-1.89) ^*^		1.46 (1.12-1.89) ^*^
***Household factors***					
**Sex of the household head**					
Male	12.7 (12.1-13.4)		Ref.		Ref.
Female	12.5 (10.9-14.2)		1.20 (1.03-1.40) ^*^		1.18 (1.01-1.37) ^*^
**Household size**					
1-4	13.2 (11.9-14.5)		0.81 (0.68-0.96) ^*^		0.81 (0.68-0.97) ^*^
5+	12.5 (11.8-13.3)		Ref.		Ref.
***Environmental factors***					
**Source of drinking water**					
Improved	14.5 (13.4-15.8)		Ref.		Ref.
Unimproved	11.8 (11.1-12.6)		0.80 (0.69-0.92)		0.82 (0.69-0.96) ^*^
**Child stool disposal**					
Safe	11.8 (10.3-13.4)		Ref.		Ref.
Unsafe	12.8 (12.1-13.5)		1.04 (0.86-1.25)		1.04 (0.86-1.26)
***Community-level characteristics***					
**Residence**					
Urban	13.3 (8.4-20.4)			1.30 (0.90-1.88)	1.67 (1.09-2.54) ^*^
Rural	12.7 (12.1-13.3)			Ref.	Ref.
**Region**					
Agrarian	12.5 (11.7-13.4)			1.06 (0.81-1.37)	1.10 (0.82-1.49)
Pastoralist	12.9 (12.0-13.9)			1.24 (0.95-1.63)	1.27 (0.93-1.74)
City administration	12.7 (5.6-26.2)			Ref.	Ref.
**Survey years**					
EDHS-2005	1.13 (0.96-1.32)			1.13 (0.96-1.32)	1.01 (0.80-1.25)
EDHS-2011	0.94 (0.83-1.06)			0.94 (0.83-1.06)	0.98 (0.83-1.16)
EDHS-2016	Ref.			Ref.	Ref.
**Random effects**					
**Variance (SD)**		0.1719 (0.0019)	0.1093 (0.0035)	0.1671 (0.0019)	0.1077 (0.0035)
**ICC (%)**		4.96	3.21	4.83	3.17
**MOR**		1.48	1.36	1.47	1.36
**PCV (%)**		Ref.	36.4	2.79	37.3
**Model fit statistics**					
**AIC**		9351.60	6934.13	9348.49	6934.79
**BIC**		9366.23	7166.39	9399.73	7202.25
**Log- likelihood (LL)**		-4673.7998	-3434.06	-4667.24	-3429.39
**Deviance**		9,347.59	6,868.13	9,334.49	6,858.79

*p-value < 0.05,

**p-value< 0.001; SE: Standard error; ICC: Intra-class Correction Coefficient; MOR: Median Odds Ratio; PCV: Proportional Change in Variance; AIC: Akaike’s Information Criterion; BIC: Bayesian Information Criteria; LL: Log-likelihood.

#### Factors associated with underweight among children 0–59 months in poor households.

Table 6 presents the multilevel multivariable analysis results of factors associated with underweight in children. From the pooled data regression, the odds of being underweight were 1.37 times higher among male children than females (AOR: 1.37, 95%CI: 1.22–1.54). Children in the age group of 0–5 months (AOR: 0.12, 95%CI: 0.09–0.16), 6–11 months (AOR: 0.31, 95%CI: 0.23–0.39), and 12–23 months (AOR: 0.60, 95%CI: 0.48–0.75) had lower odds of being underweight compared with those 36–59 months of age, respectively. Children who were not currently breastfed had 23% reduced odds of being underweight compared to their breastfed peers (AOR: 0.77, 95%CI: 0.65–0.90). Compared to their counterparts, children with diarrhea (AOR: 1.22, 95%CI: 1.04–1.43), those born to mothers without any formal education (AOR: 1.39, 95%CI: 1.18–1.64), those of very short height mothers (AOR: 2.40, 95%CI: 1.64–3.51), and those of short height mothers (AOR: 1.63, 95%CI: 1.44–1.86) had significantly higher odds of being underweight. The odds of childhood underweight were reduced among children with normal BMI mothers (AOR: 0.63, 95% CI: 0.55–0.71) and overweight or obese mothers (AOR: 0.39, 95% CI: 0.25–0.59) compared to those born of children with underweight mothers. Children from households that practice open defecation had two times higher odds of being underweight than children from households with improved sanitation facilities (AOR: 2.03, 95%CI: 1.24–3.33) (Table 6).

**Table 6:** Multilevel multivariable binary logistic regression analysis of factors associated with underweight in children aged 0-59 months in Ethiopia, EDHS (2005-2016).

	Underweight %, (95%CI)	Model 1	Model 2	Model 3	Model 4
			AOR (95%CI)	AOR (95%CI)	AOR (95%CI)
**Variables**					
***Child factors***					
**Sex**					
Male	34.4 (33.1-35.6)		1.37 (1.22-1.54)^**^		1.37 (1.22-1.54)^**^
Female	31.2 (30.0-32.5)		Ref.		Ref.
**Age (months)**					
**< 6**	9.8 (8.2-11.8)		0.12 (0.08-0.15)^**^		0.12 (0.09-0.16)^**^
6-11	25.1 (22.6-27.7)		0.29 (0.23-0.38)^**^		0.31 (0.23-0.39)^**^
12-23	35.1 (33.1-37.2)		0.58 (0.47-0.71)^**^		0.60 (0.48-0.75)^**^
24-35	36.6 (34.5-38.7)		0.95 (0.78-1.15)		0.99 (0.81-1.21)
36-59	37.5 (36.1-38.9)		Ref.		Ref.
**Size of the child at birth**					
Larger	27.2 (25.6-28.7)		Ref.		Ref.
Average	31.9 (30.6-33.4)		1.31 (1.12-1.53)^**^		1.32 (1.13-1.53)^**^
Small	39.7 (38.1-41.4)		2.06 (1.76-2.41)^**^		2.05 (1.75-2.40)^**^
**Birth order**					
Firstborn	32.0 (29.8-34.3)		Ref.		Ref.
2-4	34.1 (32.8-35.5)		1.01 (0.82-1.23)		1.01 (0.82-1.23)
5+	31.8 (30.5-33.2)		0.99 (0.77-1.29)		1.04 (0.77-1.29)
**Full vaccination**					
Yes	32.4 (30.1-34.8)		Ref.		Ref.
No	32.7 (31.6-33.7)		1.05 (0.89-1.23)		1.04 (0.89-1.22)
**Vitamin A last 6 months**					
Yes	32.8 (31.5-34.2)				
No	32.9 (31.7-34.1)				
**Currently breastfeeding**					
Yes	32.5 (31.6-33.6)		Ref.		Ref.
No	33.7 (31.9-35.5)		0.77 (0.65-0.89)^*^		0.77 (0.65-0.90)^*^
**Early initiation of breastfeeding**					
Yes	30.5 (29.2-31.9)		Ref.		Ref.
No	33.3 (31.5-35.1)		1.04 (0.92-1.18)		1.04 (0.91-1.17)
**Birth interval**					
Less than 33 months	32.7 (31.6-33.7)		1.01 (0.76-1.34)		1.02 (0.77-1.35)
≥ 33 months	33.2 (31.6-34.8)		Ref.		Ref.
**Diarrhea**					
Yes	40.0 (37.6-42.5)		1.23 (1.05-1.44)^*^		1.22 (1.04-1.43)^*^
No	31.6 (30.7-32.6)		Ref.		Ref.
**Fever**					
Yes	38.2 (36.0-40.5)		1.17 (0.98-1.39)		1.16 (0.97-1.38)
No	31.8 (30.8-32.8)		Ref.		Ref.
**Cough**					
Yes	34.9 (32.9-36.9)		0.98 (0.82-1.16)		0.98 (0.82-1.16)
No	32.4 (31.3-33.3)		Ref.		Ref.
***Parental factors***					
**Mother’s age**					
15-17	29.7 (21.3-39.7)		1.22 (0.62-2.38)		1.21 (0.62-2.37)
18-24	30.8 (29.1-32.7)		1.06 (0.76-1.50)		1.06 (0.75-1.49)
25-34	34.4 (33.1-35.6)		1.11 (0.84-1.48)		1.11 (0.84-1.47)
35-49	31.7 (30.0-33.4)		Ref.		Ref.
**Mother’s education**					
No education	34.3 (33.3-35.3)		1.39 (1.18-1.64)^**^		1.39 (1.18-1.64)^**^
Primary and above	26.3 (24.4-28.2)		Ref.		Ref.
**Maternal BMI (kg/m**^**2**^)					
<18.5	39.8 (37.9-41.8)		Ref.		Ref.
18.5 to 24.9	31.1 (30.1-32.2)		0.62 (0.55-0.71)^**^		0.63 (0.55-0.71)^**^
25 +	22.2 (18.0-27.1)		0.38 (0.25-0.58)^**^		0.39 (0.25-0.59)^**^
**Maternal stature**					
Very short	45.4 (39.9-51.0)		2.43 (1.66-3.54)^**^		2.40 (1.64-3.51)^**^
Short	37.8 (36.3-39.3)		1.64 (1.45-1.86)^**^		1.63 (1.44-1.86)^**^
Normal	29.2 (28.1-30.3)		Ref.		Ref.
**Maternal anemia**					
Yes	32.9 (31.2-34.6)		1.12 (0.98-1.27)		1.13 (0.99-1.28)
No	32.8 (31.7-33.8)		Ref.		Ref.
**Place of delivery**					
Home	33.4 (32.5-34.4)		1.12 (0.92-1.35)		1.07 (0.87-1.31)
Health facility	26.1 (23.3-29.1)		Ref.		Ref.
***Household factors***					
**Household size**					
1-4	33.4 (31.6-35.2)		0.89 (0.76-1.04)		0.89 (0.76-1.05)
5+	32.7 (31.7-33.7)		Ref.		Ref.
**Sanitation facility**					
Improved	28.7 (24.4-33.5)		Ref.		Ref.
Unimproved	29.7 (28.1-31.4)		1.35 (0.94-1.93)		1.35 (0.94-1.93)
Open defecation	34.3 (33.2-35.3)		1.44 (1.02-2.03)^*^		1.43 (1.01-2.02)^*^
**Source of drinking water**					
Improved	35.7 (34.1-37.3)		Ref.		Ref.
Unimproved	31.4 (30.4-32.5)		0.87 (0.76-0.99)^*^		0.87 (0.74-1.01)
**Time to get a water source**					
On-premise	34.1 (26.6-42.5)		Ref.		Ref.
≤ 30 min	32.0 (30.8-33.2)		0.47 (0.30-0.72)^*^		0.47 (0.30-0.74)^*^
31-60 min	32.5 (30.7-34.3)		0.47 (0.31-0.74)^*^		0.48 (0.31-0.75)^*^
>60 min	35.7 (33.7-37.8)		0.46 (0.29-0.73)^*^		0.47 (0.30-0.75)^*^
**Child stool disposal**					
Safe	30.7 (28.5-32.9)		Ref.		Ref.
Unsafe	33.3 (32.3-34.2)		1.06 (0.88-1.28)		1.07 (0.88-1.29)
***Community-level characteristics***					
**Residence**					
Urban	21.3 (15.1-29.3)			0.73 (0.53-1.01)	1.07 (0.68-1.68)
Rural	32.9 (32.1-33.8)			Ref.	Ref.
**Region**					
Agrarian	34.7 (33.5-35.9)			0.87 (0.71-1.05)	0.95 (0.71-1.27)
Pastoralist	30.4 (29.1-31.8)			0.96 (0.78-1.18)	0.96 (0.71-1.30)
City administration	36.5 (23.8-51.5)			Ref.	Ref.
**Survey years**					
EDHS-2005	1.32 (1.17-1.50)			1.31 (1.15-1.48)^**^	1.07 (0.86-1.32)
EDHS-2011	1.24 (1.13-1.36)			1.24 (1.13-1.36)^**^	1.15 (0.99-1.33)
EDHS-2016	Ref.			Ref.	Ref.
**Random effects**					
**Variance (SD)**		0.1469 (0.0009)	0.1369 (0.0024)	0.1488 (0.0009)	0.1333 (0.0025)
**ICC (%)**		4.27	3.99	4.33	3.89
**MOR**		1.43	1.42	1.44	1.41
**PCV (%)**		Ref.	6.81	1.29	9.26
**Model fit statistics**					
**AIC**		14355.94	7157.61	14327.31	7163.63
**BIC**		14370.59	7399.87	14378.57	7439.53
**Log- likelihood (LL)**		-7175.97	-3542.80	-7156.65	-3540.81
**Deviance**		14,351.94	7,085.61	14,313.31	7,081.63

*p-value < 0.05,

**p-value< 0.001; SE: Standard error; ICC: Intra-class Correction Coefficient; MOR: Median Odds Ratio; PCV: Proportional Change in Variance; AIC: Akaike’s Information Criterion; BIC: Bayesian Information Criteria; LL: Log-likelihood.

### Survey-specific results of factors associated with childhood undernutrition

#### Factors associated with stunting.

S4–S6 Files shows survey specific results of factors associated with childhood undernutrition among children aged 0–59 months in poor Ethiopian households.

In all survey years (i.e., EDHS-2005 (S4 File), EDHS-2011 (S5 File) and EDHS-2016 (S6 File)) the most significant predictors of stunting were: the male sex (EDHS-2005 and EDHS-2011), children aged 24–35 months, children perceived as smaller by their mothers, children of the mother with very short and short height, children having diarrhea (EDHS-2011), and children from households having unimproved toilet facility and those practiced open defecation (EDHS-2016).

#### Factors associated with wasting.

In all survey years (i.e., EDHS-2005, EDHS-2011, and EDHS-2016) the most significant factors positively associated with wasting include: male sex, child age 0–5, 6–11, and 12–25 months (EDHS-2005 and EDHS-2011), children perceived as smaller by their mothers, children having diarrhea (EDHS-2011), fever (EDHS-2011), not exposed to radio or television (EDHS-2011), children from pastoralist communities (EDHS-2011), those born in the home (EDHS-2016), and urban residency (EDHS-2016) (S4–S6 Files).

#### Factors associated with underweight.

In all EDHS-2005, EDHS-2011, and EDHS-2016 the consistent factors positively associated with being underweight among children aged 0–59 months were: male sex, children perceived as smaller by their mothers, children born of a mother with no education, children born to mothers of very short and short height, children having diarrhea and fever (EDHS-2011), a child born to mothers of age 25–34 years (EDHS-2011), maternal anemia (EDHS-2016), and urban residency (EDHS-2016) (S4–S6 Files).

### Multilevel analysis (random-effects analysis)

The empty model indicated that 4.52%, 4.96%, and 4.27% of the total variance in stunting, wasting, and underweight, respectively, was due to variations in characteristics between clusters. The variability between clusters decreased when subsequent models were added. Additionally, the median odds ratio (MOR) confirmed that community-level factors influenced childhood undernutrition (stunting, wasting, and underweight). For example, the MOR for stunting in the empty model was 1.45, suggesting significant variation between communities (clustering), as the MOR was higher than the reference value (MOR = 1). The unexplained community-level variation in stunting, wasting, and underweight decreased when all factors were included in the model. This suggests that although individual- and community-level factors were considered, the clustering effect remained statistically significant in the full model. The models were compared using deviance, and Model 4 was selected as the best fit, as it had the lowest deviance value (Tables 4–6).

## Discussion

Childhood undernutrition is a significant global public health plague, closely linked to poverty. There is a lack of robust evidence identifying factors associated with undernutrition in under-five children from poor households in Ethiopia, despite the persistent high burden of childhood undernutrition in the country. This study aimed to identify factors associated with childhood undernutrition in poor Ethiopian households. The finding revealed that in poor households 47.5% of children under the age of five were stunted, 12.7% wasted, and 32.8% underweight. A notable decline in the prevalence of all forms of child undernutrition was documented between 2005 and 2016 in poor households in Ethiopia. The most significant factors positively associated with stunting, wasting, and being underweight were male gender, younger age, having diarrhea, children perceived as smaller at birth by their mothers, children of mothers with limited education, maternal short stature, and children from households with unimproved sanitation facilities. On the other hand, it was observed that higher odds of wasting were associated with the presence of fever in the 2 weeks prior to the survey, children from female-headed households, households without television exposure, and residents of urban households.

### The burden of childhood undernutrition in poor Ethiopian households: Implications for public health interventions

The results of the current study revealed that stunting remains a significant public health issue in Ethiopia, with an overall prevalence of 47.5%. This staggering figure underscores the fact that stunting continues to pose a major public health challenge in poor households, as the prevalence of stunting significantly surpasses the national average of 37% – an already concerning statistic [19]. The observed prevalence of stunting in Ethiopia should be categorized as very high as per international standards (≥ 40%) [58], needing concerted effort to address it. Despite slight improvements observed over the years, the burden of stunting has persisted at alarmingly high rates in survey-specific findings: 53.9% in 2005, 48.2% in 2011, and 43.9% in 2016. These figures indicate that inadequate progress has been made in addressing the issue of child undernutrition especially within impoverished households in Ethiopia. The persistent nature of stunting within these vulnerable populations is a pressing matter, demanding immediate and targeted interventions to mitigate its detrimental consequences of childhood undernutrition. Moreover, reinforcing existing strategies such as the People in Need (PIN) project [59], which has used the Positive Deviance approach to promote positive infant and young child feeding practices and improve nutrition, or developing new approaches aimed at reducing undernutrition in all its forms, is essential for fostering a healthier future for disadvantaged Ethiopian households and breaking the vicious cycle of undernutrition.

Similarly limited progress was seen in the reduction of childhood wasting and underweight in poor households in Ethiopia. The overall prevalence of wasting and underweight was 12.7% and 32.8%, respectively. Both the prevalence of wasting and underweight among under-five children in poor households in Ethiopia did not drop between 2005 and 2016. The observed prevalence of wasting and underweight was also higher than the recent national food and nutrition strategy baseline survey, which reported that the national prevalence of wasting and underweight in children under 5 years was 11% and 22%, respectively [60].

### The role of gender in childhood undernourishment in poor households

The present study revealed that male children had a higher likelihood of experiencing stunting, wasting, and being underweight compared to female children. Similarly, in each survey-specific analysis, male children consistently showed greater odds of being undernourished. This result aligns with findings from several observational studies conducted in similar low-income regions, such as Kenya [61], Zambia [62], Senegal [63], Ghana [64], Nigeria [65], Indonesia [66,67], and Ethiopia [31]. Male children are more likely to be undernourished compared to their female counterparts due to biological and environmental determinants; and a combination of both. The gender-based health disparity, which may be attributed to males being more susceptible than females to different infections as indicated in earlier works of literature, may also be another reason and viable explanation for the current findings [68–70]. On the other hand, male children are often encouraged to engage in outdoor activities and explore their surroundings at an early age. Therefore, it is essential for policymakers and public health officials to acknowledge these gender-based differences when designing targeted interventions that address the specific challenges faced by male children in disadvantaged households.

### Age-specific patterns of undernourishment in children

In this study, children in the age groups of 0–5 months, 6–11 months, and 12–23 months had lower odds of being underweight and stunted compared to those aged 36–59 months. This can be explained by the nature of stunting and underweight as indicators of long-term undernutrition, which may have started during fetal development [71]. These conditions are often the result of chronic nutritional deficiencies, which typically become more visible in older children as they grow. Additionally, children in the younger age groups are more likely to be in the rapid growth phase, which may temporarily mask the long-term effects of undernutrition. Previous studies in Eswatini [72], Nepal [73], Ghana [74], and Ethiopia [75] highlighted that the prevalence of stunting increased with increasing age, with the lowest prevalence observed in younger children [76,77]. On the other hand, young children in the age group of (0–5, 6–11, and 12–23 months) had higher odds of wasting compared with those 36–59 months of age, respectively. This is somewhat expected since wasting which is an acute undernourishment (i.e., characterized by low weight-for-height or low weight-for-length) is more common among younger children [78], due to their immature immune systems [24,79]. Younger children, particularly those under 2 years old, are in a phase of rapid growth and development, both physically and metabolically. During this period, they are more vulnerable to acute nutritional deficiencies, which can lead to wasting. In contrast, older children are less susceptible to wasting primarily because their growth rate slows down as they age, allowing their bodies to better cope with temporary periods of nutritional deficiency [80,81]. Additionally, older children have larger nutritional reserves and are better able to cope with short-term deficiencies or illness without showing immediate signs of wasting. Their bodies can efficiently utilize stored nutrients, making them less vulnerable to rapid weight loss compared to younger children, who have fewer reserves and are more susceptible to malnutrition. Further research is required to examine the underlying factors contributing to and to develop effective interventions that address the nutritional needs of children in different age groups in poor households.

### The link between diarrheal illness and undernourishment in poor households: A modifiable risk factor

Consistent with other similar studies, childhood diarrhea was positively associated with childhood undernourishment [39,82–84]. In our analysis, compared to their counterparts, children with diarrhea had significantly higher odds of being stunted, wasted, and underweight. Studies from other similar low and middle income settings, such as Bangladesh [85] and Tanzania [86] reported similar findings. Diarrhea is a common condition that leads to undernourishment in children due to the interaction of multiple factors, including nutrient loss, decreased appetite, and dehydration. The bidirectional relationship between diarrheal and undernutrition should also be another possible explanation for the observed association [87,88].

### The role of size of the child at birth in childhood undernourishment

We found that children who were perceived by their mothers to be smaller sizes than normal at birth were more likely to be stunted and wasted, and similar findings have been found in previous literature [27,89]. The impact of low birth weight on childhood undernutrition was significant. Children born with low birth weight (LBW) are inherently at a higher risk of experiencing both stunting and wasting from birth, conditions that reflect the immediate consequences of inadequate prenatal growth [90]. These early growth deficits not only demonstrate the intergenerational effects of stunting but also establish a pathway for continued poor nutritional outcomes throughout the individual’s life. LBW children are at greater risk of malnutrition due to several factors. These include having limited nutrient reserves at birth, which can hinder their growth and development, experiencing feeding difficulties, which can lead to inadequate nutrition during critical periods of growth, and being more susceptible to infections, which can impair nutrient absorption and increase nutritional needs. Finally, if these issues are not addressed early, they can lead to persistent undernutrition and long-term developmental challenges [91–94]. Further, the observed association of birth size with childhood undernutrition could be attributed to a combination of biological, environmental, nutritional, and maternal nutrition factors [95,96].

### The role of maternal education in influencing childhood undernourishment

The odds of stunting and being underweight among children born to mothers with no education were higher compared to those reporting having primary or higher education. This result was predicted, as mothers without formal education often have undernourished children due to lack of knowledge about proper nutrition and childcare, as well as restricted access to resources and healthcare. This is often compounded by socioeconomic challenges and cultural beliefs that may not align with modern nutritional guidelines. These factors increase the risk of poor nutrition and health outcomes for their children [97,98].

### Maternal stature and childhood undernourishment

In the present study, short maternal stature was associated with stunting, which is in conformity with several studies consistently showing a clear relationship between maternal height and anthropometric failure [99–101]. Maternal height is often used as a marker to assess the intergenerational health linkages between a mother and her offspring, emphasizing the intergenerational cycle of stunting that affects multiple generations. Research has repeatedly shown that maternal height is linked to stunting in low- and middle-income countries [101–104]. Additionally, the effect of maternal nutritional status on childhood undernutrition can be further explained by low maternal nutrient stores, which lead to nutrient competition between the mother and child both during pregnancy and breastfeeding. This competition affects growth and development in children, especially when living in the same environment and consuming similarly low-nutrient foods.

### Maternal BMI and childhood undernourishment

The growing body of evidence connects maternal BMI to children’s nutritional status [101,104,105]. In our analysis, compared with children with underweight mothers those with normal BMI and/or overweight or obese mothers were at lower odds of wasting and childhood underweight. Previous results showed maternal underweight has been closely related to child undernutrition and adverse birth outcomes [105–107]. The possible explanation for the observed association between maternal underweight and child undernutrition might be due to inadequate nutrient transfer, increased susceptibility to infections, inter-generational cycles of malnutrition, and socioeconomic factors [101].

### Household WASH and childhood undernourishment

Our analysis showed that the odds of children suffering from stunting and underweight were higher for children from households with unimproved toilet facilities and open defecation compared to children from households with improved sanitation facilities. Sanitation facilities play a crucial role in promoting good child health and preventing childhood undernourishment. One of the most important ways in which sanitation facilities influence childhood undernourishment is through open defecation or poor household waste disposal, which can contaminate nearby water sources and lead to the spread of diarrheal illness. Diarrhea can cause severe dehydration, nutrient loss, and reduced appetite, ultimately contributing to undernutrition in children [47,108–110]. In addition to direct health impacts, poor sanitation facilities can indirectly affect childhood undernourishment through their influence on hygiene practices [111]. Similar to the previous study in Ethiopia [47], we additionally identified a reverse correlation between using unimproved drinking water sources and a lower likelihood of wasting. The inverse relationship between using unimproved drinking water sources and a lower risk of wasting may be explained by the absence of pertinent data on the bacteriological quality and other properties of drinking water in the EDHS datasets.

### Media exposure and wasting

We also found that media exposure had a significant effect on child wasting, and similar findings have been found in previous studies [112,113]. It is postulated that this is a result of the increased exposure to health information since media plays a crucial role in shaping public perceptions, attitudes, and behaviors, including those related to nutrition and health. These significantly influence mothers’ or caregivers’ knowledge and practices regarding child feeding practices and overall child nutritional status.

### Place of residence and wasting

Place of residence has been identified as a potential risk factor for childhood undernutrition. Our findings indicated that children from urban households were more likely to be wasted. One of the primary reasons for the observed association might be due to the higher prevalence of poverty in urban areas [114]. In the Ethiopian context, many urban environments also pose challenges related to sanitation and hygiene, which increase the risk of infections that can contribute to wasting. Furthermore, urban settings often experience higher levels of environmental pollution, which can have detrimental effects on children’s nutritional status.

Place of residence has been identified as a potential risk factor for childhood undernutrition. Our findings suggest that children from urban households are at increased risk of undernutrition. In many urban environments, challenges related to sanitation and hygiene are prevalent, which heighten the risk of infections that can contribute to conditions like wasting. Additionally, urban areas often face higher levels of environmental pollution, including water contamination, which can negatively impact children’s health and nutritional status. These factors, combined with limited access to quality healthcare and nutrition, exacerbate the vulnerability of children in urban settings to undernutrition in Ethiopia.

### Association between the number of household members and childhood wasting

The association between the number of household members and childhood undernutrition is a complex issue that is not universal and can vary depending on the context and specific circumstances [115,116]. In this study, households with fewer household members have lower odds of wasting. This connection could be attributed to several factors, including better food consumption, being less prone to food insecurity, and less competition. With fewer members, children find it easier to eat enough varied and nutritious food. Furthermore, mothers could not find it difficult to provide for their children’s nutritional needs, especially in poor, deviant households. Previous studies had found that larger households were positively associated with childhood undernutrition and household food insecurity [117–119].

### Strengths and limitations

One of the strengths of this study is the use of nationally representative data with a large sample size and statistical power that is adequate to analyze the relationships between different levels of variables and child undernutrition in children under five years old in poor households. Additionally, the multilevel modeling approach used in the analysis and the use of sampling weight in our analyses could also reduce potential bias. The study has certain limitations. First, since this study was based on cross-sectional data, it could not provide evidence of a causal relationship between outcomes and independent variables. Second, the selection of variables analyzed based on their availability in the dataset, and data on potential confounders, including household food security, the behavior of the parents, and underlying disease conditions, were not included in the analysis. Third, some data were based on the mothers’ recall, which might have been subjected to recall bias. Fourth, the pooling of the data may be affected by heterogeneity across survey years. Finally, as the outcome is common (e.g., prevalence above 10%), ORs may exaggerate the association, making them less reliable as a risk measure. Therefore, the interpretation of the study findings should be approached with caution. We recommend that future studies examine the specific factors contributing to undernutrition in wealthier households, such as dietary habits, access to healthcare, and other household determinants, to identify potential areas for intervention.

## Conclusions

The prevalence of undernutrition (stunting, wasting, and underweight) among children under the age of five in poor households was high. Limited progress has been made in reducing childhood undernutrition, with the overall prevalence remaining high. These prevalence figures of undernutrition also surpass the national average and persist at alarmingly high rates in survey-specific findings as well, highlighting the need for urgent and targeted interventions to mitigate the detrimental consequences in disadvantaged households. The most significant factors positively associated with childhood undernutrition consist of child-related factors (male gender, younger age, having diarrhea, children perceived as smaller by their mothers), maternal factors (uneducated mothers, maternal short stature, and being underweight), household factors (unimproved sanitation facilities), and at the community level (urban residence). To address childhood undernutrition, interventions should target modifiable factors at multiple levels. Maternal education programs can improve nutrition and caregiving practices while improving maternal health through better nutrition and prenatal care can reduce the risk of undernutrition in children. Enhancing sanitation facilities and promoting hygiene can help prevent childhood infections like diarrhea, which contribute to wasting. Additionally, community-based nutritional programs focused on vulnerable groups, such as young children and those in urban areas with higher environmental risks, can further reduce undernutrition rates. Finally, poverty reduction initiatives are essential to address the root causes of undernutrition, as improving economic conditions and access to resources can positively impact a child’s overall health and well-being.

## Supporting information

S1 File Prevalence of stunting among children 0–59 months in poor households with different characteristics for the survey year 2005, 2011 and 2016. (DOCX)

S2 File Prevalence of wasting among children 0–59 months in poor households with different characteristics for the survey year 2005, 2011 and 2016. (DOCX)

S3 File Prevalence of underweight among children 0–59 months in poor households with different characteristics for the survey year 2005, 2011 and 2016. (DOCX)

S4 File Multilevel bivariable binary logistic regression analysis of factors associated with stunting, wasting and underweight in children aged 0–59 months in Ethiopia, EDHS-2005. (DOCX)

S5 File Multilevel bivariable binary logistic regression analysis of factors associated with stunting, wasting and underweight in children aged 0–59 months in Ethiopia, EDHS-2011.
(DOCX)

S6 File Multilevel bivariable binary logistic regression analysis of factors associated with stunting, wasting and underweight in children aged 0–59 months in Ethiopia, EDHS-2016. (DOCX)
