# Supplementary material for: Factors associated with childhood undernutrition in poor Ethiopian households: Implications for public health interventions
Source: PLoS One. 2025 May 9;20(5):e0323332. doi: 10.1371/journal.pone.0323332 (PMC12063910; doi:10.1371/journal.pone.0323332)
Supplement: S1 File — (DOCX) [file pone.0323332.s001.docx]

**Supplementary File 1: Prevalence of stunting among children 0-59 months in poor households with different characteristics for the survey year 2005, 2011 and 2016.**

|  | **Stunting prevalence, 95%CI** | | | |
| --- | --- | --- | --- | --- |
| **Variables** | **Pooled (EDHS 2005-2016)** | **EDHS-2005** | **EDHS-2011** | **EDHS-2016** |
| **Child factors** |  |  |  |  |
| **Sex** |  |  |  |  |
| Male | 48.9 (47.6-50.2) | 55.5 (52.3-58.6) | 49.3 (47.2-51.3) | 45.7 (43.6-47.7) |
| Female | 46.0 (44.7-47.4) | 52.4 (49.1-55.6) | 47.1 (45.1-49.2) | 42.1 (40.1-44.2) |
| **Age (months)** |  |  |  |  |
| **< 6** | 14.2 (12.2-16.4) | 14.0 (9.4-20.4) | 10.4 (7.8-13.6) | 17.8 (14.5-21.5) |
| 6-11 | 26.8 (24.3-29.4) | 38.3 (31.7-45.3) | 26.3 (22.6-30.3) | 22.3 (18.7-26.4) |
| 12-23 | 49.5 (47.3-51.7) | 54.5 (49.4-59.5) | 49.9 (46.5-53.3) | 46.9 (43.5-50.3) |
| 24-35 | 57.5 (55.4-59.7) | 64.4 (59.3-69.2) | 60.0 (56.5-63.3) | 52.5 (49.2-55.8) |
| 36-59 | 55.1 (53.7-56.6) | 60.6 (57.2-63.9) | 57.1 (54.8-59.3) | 50.5 (48.1-52.8) |
| **Size of the child at birth** |  |  |  |  |
| Larger | 43.8 (42.1-45.5) | 48.9 (44.9-53.1) | 45.2 (42.6-47.8) | 40.0 (37.3-42.7) |
| Average | 47.7 (46.1-49.2) | 55.3 (51.6-58.9) | 47.7 (45.3-50.1) | 44.6 (42.3-46.9) |
| Small | 50.9 (49.1-52.6) | 57.5 (53.5-61.5) | 51.4 (48.8-53.9) | 47.2 (44.5-49.9) |
| **Birth order** |  |  |  |  |
| First born | 48.9 (46.5-51.3) | 54.5 (48.5-60.4) | 51.3 (47.6-54.9) | 44.1 (40.4-47.9) |
| 2-4 | 47.3 (45.8-48.7) | 54.4 (50.9-57.9) | 48.4 (46.1-50.6) | 43.2 (41.0-45.4) |
| 5+ | 47.1 (45.6-48.6) | 53.4 (49.9-56.7) | 46.8 (44.5-49.1) | 44.6 (42.4-46.9) |
| **Full vaccination** |  |  |  |  |
| Yes | 51.2 (48.7-53.7) | 52.7 (45.6-59.7) | 54.7 (51.1-58.3) | 46.4 (42.4-50.4) |
| No | 45.5 (44.4-46.7) | 54.5 (51.9-56.9) | 46.4 (44.8-48.1) | 37.2 (35.1-39.3) |
| **Vitamin A last 6 months** |  |  |  |  |
| Yes | 51.0 (49.5-52.4) | 54.4 (51.1-57.6) | 54.3 (40.6-44.6) | 45.0 (42.7-47.4) |
| No | 44.6 (43.3-45.8) | 53.2 (49.9-56.3) | 42.6 (40.7-44.6) | 43.2 (41.3-45.1) |
| **Currently breastfeeding** |  |  |  |  |
| Yes | 46.2 (45.1-47.3) | 53.2 (50.6-55.8) | 45.8 (44.1-47.4) | 43.6 (41.9-45.3) |
| No | 51.2 (49.3-53.1) | 56.4 (51.8-60.9) | 56.2 (53.2-59.2) | 44.8 (42.1-47.7) |
| **Early initiation of breastfeeding** |  |  |  |  |
| Yes | 44.4 (42.9-45.9) | 52.9 (49.6-56.4) | 44.8 (42.3-47.4) | 40.8 (38.7-42.9) |
| No | 46.8 (44.9-48.7) | 49.3 (44.3-54.3) | 47.6 (45.1-50.2) | 44.0 (40.5-47.6) |
| **Birth interval** |  |  |  |  |
| 7- 33 months / short/ | 47.3 (46.1-48.4) | 54.0 (51.2-56.8) | 47.9 (46.2-49.7) | 43.9 (42.2-45.7) |
| ≥ 33 months /non-short/ | 47.9 (46.2-49.6) | 53.9 (50.1-57.7) | 48.7 (46.0-51.3) | 44.0 (41.3-46.7) |
| **Diarrhoea** |  |  |  |  |
| Yes | 50.5 (47.9-53.0) | 54.8 (49.7-59.8) | 49.3 (45.3-53.2) | 48.7 (44.4-53.1) |
| No | 47.0 (46.0-48.0) | 53.9 (51.4-56.4) | 47.9 (46.4-49.6) | 43.4 (41.8-44.9) |
| **Fever** |  |  |  |  |
| Yes | 46.9 (44.6-49.3) | 51.9 (46.8-57.0) | 45.1 (41.7-48.6) | 46.4 (42.4-50.4) |
| No | 47.6 (46.5-48.6) | 54.6 (52.1-57.1) | 48.8 (47.2-50.4) | 43.6 (42.0-45.2) |
| **Cough** |  |  |  |  |
| Yes | 45.4 (43.2-47.5) | 51.2 (45.8-56.5) | 44.2 (40.9-47.5) | 44.2 (40.8-47.6) |
| No | 47.9 (46.9-49.0) | 54.7 (52.2-57.2) | 49.2 (47.5-50.8) | 43.9 (42.2-45.5) |
| **Parental factors** |  |  |  |  |
| **Mother's age** |  |  |  |  |
| 15-17 | 43.2 (33.2-53.6) | 56.7 (31.9-78.6) | 33.9 (18.5-53.7) | 44.0 (30.7-58.2) |
| 18-24 | 43.2 (33.2-53.7) | 51.3 (46.7-55.9) | 48.5 (45.4-51.5) | 43.1 (40.1-46.2) |
| 25-34 | 47.9 (46.6-49.3) | 55.4 (52.2-58.7) | 48.9 (46.8-50.9) | 44.1 (42.0-46.1) |
| 35-49 | 47.3 (45.4-49.1) | 53.7 (49.4-57.9) | 46.9 (44.0-49.8) | 44.5 (41.5-47.5) |
| **Mother's education** |  |  |  |  |
| No education | 47.8 (46.8-48.9) | 53.7 (51.3-56.0) | 48.0 (46.4-49.6) | 44.7 (43.1-46.4) |
| Primary and above | 45.8 (43.6-48.0) | 57.0 (49.6-64.1) | 49.1 (45.6-52.6) | 41.1 (38.0-44.2) |
| **Mother's currently working** |  |  |  |  |
| Yes | 49.1 (47.2-51.0) | 58.6 (53.8-63.2) | 47.2 (44.5-49.9) | 47.5 (44.4-50.7) |
| No | 46.9 (45.8-48.0) | 52.6 (50.1-55.2) | 48.6 (46.8-50.3) | 42.9 (41.3-44.6) |
| **Maternal BMI (kg/m^2^)** |  |  |  |  |
| <18.5 | 49.1 (47.1-51.0) | 56.6 (51.8-61.2) | 49.1 (46.0-52.1) | 45.7 (42.6-48.9) |
| 18.5 to 24.9 | 47.1 (46.0-48.2) | 53.1 (50.5-55.7) | 48.1 (46.4-49.8) | 43.4 (41.7-45.1) |
| 25 + | 45.1 (39.8-50.6) | 55.1 (42.7-66.9) | 40.7 (31.2-50.9) | 43.8 (36.5-51.5) |
| **Maternal stature** |  |  |  |  |
| Very short | 59.6 (54.0-64.9) | 63.9 (51.2-74.9) | 61.6 (52.8-69.6) | 55.3 (46.4-64.0) |
| Short | 55.4 (53.8-56.9) | 61.3 (57.6-64.9) | 55.8 (53.5-58.1) | 52.0 (49.5-54.5) |
| Normal | 42.0 (40.8-43.2) | 48.9 (46.0-51.8) | 42.1 (40.2-44.1) | 39.1 (37.3-40.9) |
| **Maternal anemia** |  |  |  |  |
| Yes | 45.5 (43.7-47.2) | 49.1 (45.0-53.3) | 47.1 (43.8-50.3) | 43.2 (40.8-45.7) |
| No | 48.2 (47.1-49.4) | 56.4 (53.6-59.2) | 48.6 (46.9-50.2) | 44.3 (42.5-46.2) |
| **Place of delivery** |  |  |  |  |
| Home | 47.8 (46.9-48.8) | 53.9 (51.7-56.2) | 48.2 (46.7-49.7) | 44.4 (42.8-46.1) |
| Health facility | 42.9 (39.7-46.3) | 63.8 (44.9-79.2) | 47.4 (38.9-56.1) | 41.3 (37.8-50.0) |
| **Listening to radio** |  |  |  |  |
| Yes | 46.9 (44.9-48.8) | 49.8 (44.8-54.9) | 47.1 (44.7-49.5) | 44.4 (40.4-48.4) |
| Not at all | 47.6 (46.5-48.7) | 54.9 (52.5-57.5) | 48.8 (46.9-50.7) | 43.9 (42.3-45.5) |
| **Watching television** |  |  |  |  |
| Yes | 47.0 (44.5-49.5) | 52.9 (34.1-71.0) | 46.1 (43.2-48.9) | 50.1 (44.4-55.7) |
| Not at all | 47.5 (46.5-48.6) | 54.0 (51.7-56.2) | 48.9 (47.2-50.6) | 43.5 (41.9-45.0) |
| **Household factors** |  |  |  |  |
| **Sex of the household head** |  |  |  |  |
| Male | 47.5 (46.5-48.5) | 54.3 (51.9-56.7) | 48.2 (46.6-49.8) | 43.8 (42.2-45.4) |
| Female | 47.4 (44.8-49.9) | 51.7 (45.5-57.9) | 48.2 (44.3-52.1) | 44.7 (40.7-48.7) |
| **Household size** |  |  |  |  |
| 1-4 | 48.2 (46.2-50.2) | 56.1 (51.2-60.8) | 48.6 (45.5-51.7) | 44.8 (41.9-47.8) |
| 5+ | 47.2 (46.2-48.3) | 53.4 (50.8-55.9) | 48.1 (46.4-49.7) | 43.6 (41.9-45.3) |
| **Environmental factors** |  |  |  |  |
| **Sanitation facility** |  |  |  |  |
| Improved | 41.6 (36.8-46.7) | 57.1 (39.5-73.0) | 40.1 (33.3-47.2) | 40.5 (33.2-48.2) |
| Unimproved | 46.5 (44.7-48.3) | 59.5 (51.6-66.8) | 48.6 (45.9-51.3) | 43.3 (40.8-45.8) |
| Open defecation | 48.0 (46.9-49.2) | 53.5 (51.1-55.8) | 48.2 (46.3-50.0) | 44.6 (42.7-46.4) |
| **Source of drinking water** |  |  |  |  |
| Improved | 49.0 (47.4-50.7) | 54.8 (52.3-57.2) | 44.6 (41.2-48.0) | 43.6 (40.6-46.6) |
| Unimproved | 46.6 (45.4-47.7) | 49.7 (44.0-55.5) | 48.7 (47.1-50.3) | 44.1 (42.4-45.8) |
| **Time to get a water source** |  |  |  |  |
| On-premise | 46.2 (37.8-54.8) | 48.7 (23.3-74.8) | 47.0 (33.0-61.5) | 45.3 (34.3-56.8) |
| ≤ 30 min | 47.5 (46.3-48.8) | 55.1 (52.2-57.9) | 47.7 (45.7-49.7) | 43.8 (41.9-45.8) |
| 31-60 min | 47.9 (45.9-49.8) | 53.3 (47.8-58.6) | 47.8 (45.0-50.7) | 46.3 (43.2-49.3) |
| >60 min | 46.8 (44.6-48.9) | 51.1 (45.9-56.3) | 50.2 (46.8-53.5) | 41.1 (37.7-44.5) |
| **Child stool disposal** |  |  |  |  |
| Safe | 45.4 (43.1-47.8) | 58.5 (49.6-66.9) | 44.9 (41.6-48.2) | 43.7 (40.2-47.4) |
| Unsafe | 47.8 (46.8-48.8) | 53.9 (51.5-56.2) | 48.9 (47.2-50.5) | 43.9 (42.4-45.6) |
| **Community-level characteristics** |  |  |  |  |
| **Residence** |  |  |  |  |
| Urban | 51.7 (43.1-60.4) | 68.1 (35.7-89.1) | 26.7 (15.1-42.7) | 61.9 (50.8-72.0) |
| Rural | 47.4 (46.5-48.4) | 53.9 (51.6-56.2) | 48.4 (46.9-49.8) | 43.6 (42.1-45.1) |
| **Region** |  |  |  |  |
| Agrarian | 49.8 (48.6-51.1) | 57.9 (54.8-60.9) | 50.9 (49.0-52.8) | 45.3 (43.3-47.3) |
| Pastoralist | 44.4 (43.0-45.8) | 49.5 (46.2-52.8) | 44.3 (42.1-46.6) | 42.3 (40.1-44.4) |
| City administration | 48.5 (34.1-63.1) | 49.9 (21.0-78.9) | 45.2 (21.5-71.3) | 49.8 (30.0-69.7) |
