# Supplementary material for: Factors associated with childhood undernutrition in poor Ethiopian households: Implications for public health interventions
Source: PLoS One. 2025 May 9;20(5):e0323332. doi: 10.1371/journal.pone.0323332 (PMC12063910; doi:10.1371/journal.pone.0323332)
Supplement: S2 File — (DOCX) [file pone.0323332.s002.docx]

**Supplementary File 2: Prevalence of wasting among children 0-59 months in poor households with different characteristics for the survey year 2005, 2011 and 2016.**

|  | **Wasting prevalence, 95%CI** | | | |
| --- | --- | --- | --- | --- |
| **Variables** | **Pooled (EDHS 2005-2016)** | **EDHS-2005** | **EDHS-2011** | **EDHS-2016** |
| **Child factors** |  |  |  |  |
| **Sex** |  |  |  |  |
| Male | 14.2 (13.3-15.2) | 17.5 (15.2-19.9) | 14.9 (13.4-16.4) | 12.2 (10.9-13.6) |
| Female | 11.1 (10.3-11.9) | 12.9 (10.9-15.2) | 9.9 (8.8-11.3) | 11.5 (10.2-12.9) |
| **Age (months)** |  |  |  |  |
| **< 6** | 14.8 (12.8-17.1) | 17.1 (12.0-23.9) | 13.6 (10.7-17.1) | 15.3 (12.2-18.9) |
| 6-11 | 18.8 (16.7-21.2) | 21.2 (16.0-27.5) | 22.1 (18.7-26.0) | 14.1 (11.1-17.7) |
| 12-23 | 18.5 (16.8-20.2) | 21.5 (17.6-26.0) | 17.3 (14.9-20.1) | 18.3 (15.8-21.0) |
| 24-35 | 10.6 (9.3-12.0) | 12.5 (9.5-16.4) | 10.9 (8.9-13.3) | 9.5 (7.8-11.6) |
| 36-59 | 8.9 (8.1-9.8) | 11.9 (9.8-14.3) | 8.2 (7.0-9.5) | 8.5 (7.3-9.9) |
| **Size of the child at birth** |  |  |  |  |
| Larger | 10.7 (9.7-11.8) | 12.7 (10.2-15.6) | 10.3 (8.8-12.0) | 10.4 (8.8-12.1) |
| Average | 11.2 (10.4-12.3) | 12.9 (10.7-15.6) | 10.4 (9.0-11.9) | 11.4 (10.0-12.9) |
| Small | 16.5 (15.3-17.8) | 20.6 (17.6-24.1) | 16.9 (15.0-18.9) | 14.1 (12.3-16.1) |
| **Birth order** |  |  |  |  |
| First born | 12.4 (10.9-14.1) | 16.4 (12.4-21.3) | 12.2 (10.1-14.8) | 11.0 (8.8-13.6) |
| 2-4 | 12.9 (11.9-13.9) | 15.8 (13.5-18.6) | 12.5 (11.1-14.0) | 12.1 (10.7-13.7) |
| 5+ | 12.6 (11.6-13.6) | 14.3 (12.1-16.9) | 12.5 (11.0-14.1) | 11.9 (10.5-13.5) |
| **Full vaccination** |  |  |  |  |
| Yes | 10.7 (9.2-12.4) | 11.6 (7.8-17.0) | 8.7 (6.8-10.9) | 12.9 (10.4-15.9) |
| No | 14.1 (13.3-14.9) | 15.4 (13.7-17.3) | 13.3 (12.2-14.5) | 14.4 (12.9-16.0) |
| **Vitamin A last 6 months** |  |  |  |  |
| Yes | 12.1 (11.2-13.1) | 15.1 (12.8-17.5) | 11.4 (10.1-12.8) | 11.4 (10.0-13.0) |
| No | 13.2 (12.4-14.1) | 15.7 (13.5-18.2) | 13.5 (12.2-14.9) | 12.1 (10.9-13.4) |
| **Currently breastfeeding** |  |  |  |  |
| Yes | 13.5 (12.8-14.3) | 15.7 (13.9-17.7) | 13.9 (12.8-15.1) | 12.1 (10.9-13.2) |
| No | 10.3 (9.2-11.5) | 13.9 (11.0-17.4) | 7.4 (5.9-9.2) | 11.3 (9.7-13.3) |
| **Early initiation of breastfeeding** |  |  |  |  |
| Yes | 14.8 (13.8-15.9) | 18.3 (15.8-21.0) | 14.2 (12.5-16.1) | 13.9 (12.5-15.5) |
| No | 14.4 (13.1-15.8) | 17.3 (13.8-21.4) | 14.2 (12.5-16.1) | 13.4 (11.2-16.0) |
| **Birth interval** |  |  |  |  |
| 7- 33 months / short/ | 13.0 (12.3-13.8) | 14.9 (13.0-16.9) | 12.8 (11.6-14.0) | 12.5 (11.4-13.7) |
| ≥ 33 months /non-short/ | 11.9 (10.9-13.1) | 15.9 (13.4-18.9) | 11.6 (10.1-13.4) | 10.3 (8.7-12.0) |
| **Diarrhoea** |  |  |  |  |
| Yes | 17.6 (15.7-19.6) | 18.9 (15.3-23.2) | 20.1 (17.2-23.5) | 13.5 (10.7-16.7) |
| No | 11.9 (11.2-12.5) | 14.4 (12.7-16.2) | 11.1 (10.2-12.2) | 11.6 (10.7-12.7) |
| **Fever** |  |  |  |  |
| Yes | 18.8 (17.1-20.7) | 19.8 (16.1-24.3) | 20.5 (17.8-23.5) | 16.0 (13.3-19.1) |
| No | 11.5 (10.8-12.2) | 14.2 (12.5-16.0) | 10.7 (9.7-11.7) | 11.2 (10.2-12.2) |
| **Cough** |  |  |  |  |
| Yes | 15.2 (13.8-16.8) | 16.6 (13.0-21.0) | 15.6 (13.3-18.1) | 14.3 (12.1-16.9) |
| No | 12.1 (11.4-12.8) | 14.9 (13.3-16.8) | 11.6 (10.6-12.7) | 11.3 (10.3-12.4) |
| **Parental factors** |  |  |  |  |
| **Mother's age** |  |  |  |  |
| 15-17 | 14.3 (8.3-23.4) | 18.4 (5.6-4.6) | 5.1 (9.4-23.7) | 18.2 (9.4-32.3) |
| 18-24 | 14.1 (12.7-15.5) | 17.0 (13.8-20.8) | 14.5 (12.4-16.7) | 12.4 (10.5-14.5) |
| 25-34 | 12.3 (11.5-13.2) | 14.1 (11.9-16.5) | 11.7 (10.4-13.1) | 12.3 (11.0-13.7) |
| 35-49 | 12.1 (10.9-13.3) | 15.7 (12.8-19.0) | 12.1 (10.4-14.1) | 10.3 (8.6-12.2) |
| **Mother's education** |  |  |  |  |
| No education | 13.2 (12.5-13.9) | 15.8 (14.1-17.6) | 13.2 (12.1-14.3) | 12.0 (10.9-13.1) |
| Primary and above | 10.3 (9.0-11.7) | 10.2 (6.5-15.6) | 8.9 (7.1-11.1) | 11.4 (9.5-13.5) |
| **Mother's currently working** |  |  |  |  |
| Yes | 13.0 (11.8-14.4) | 18.5 (15.1-22.5) | 11.2 (9.6-13.1) | 13.1 (11.1-15.3) |
| No | 12.6 (11.9-13.3) | 14.3 (12.6-16.2) | 12.9 (11.8-14.1) | 11.5 (10.5-12.6) |
| **Maternal BMI (kg/m^2^)** |  |  |  |  |
| <18.5 | 16.9 (15.5-18.4) | 21.1 (17.5-25.2) | 17.5 (15.3-20.0) | 14.4 (12.3-16.7) |
| 18.5 to 24.9 | 11.5 (10.8-12.2) | 13.7 (11.9-15.6) | 10.9 (9.9-12.1) | 11.1 (10.1-12.3) |
| 25 + | 10.5 (7.6-14.4) | 10.9 (5.2-21.4) | 8.9 (4.6-16.6) | 11.3 (7.3-17.1) |
| **Maternal stature** |  |  |  |  |
| Very short | 14.8 (11.3-19.3) | 14.5 (7.7-25.7) | 10.9 (6.5-17.7) | 19.1 (13.0-27.3) |
| Short | 13.6 (12.5-14.7) | 14.8 (12.4-17.7) | 13.9 (12.4-15.6) | 12.5 (10.9-14.3) |
| Normal | 12.1 (11.3-12.9) | 15.5 (13.5-17.8) | 11.4 (10.3-12.7) | 11.2 (10.1-12.4) |
| **Maternal anemia** |  |  |  |  |
| Yes | 12.9 (11.8-14.2) | 11.2 (8.8-14.1) | 14.2 (12.1-16.6) | 12.8 (11.3-14.6) |
| No | 12.6 (11.9-13.4) | 16.8 (14.8-19.1) | 12.1 (11.1-13.3) | 11.4 (10.2-12.6) |
| **Place of delivery** |  |  |  |  |
| Home | 12.9 (12.3-13.6) | 15.4 (13.8-17.1) | 12.5 (11.6-13.6) | 12.2 (11.2-13.3) |
| Health facility | 9.9 (8.1-12.1) | 7.9 (2.1-25.3) | 9.2 (5.3-15.6) | 10.1 (8.1-12.5) |
| **Listening to radio** |  |  |  |  |
| Yes | 11.4 (10.3-12.7) | 16.1 (12.8-20.2) | 10.6 (9.2-12.1) | 10.8 (8.5-13.6) |
| Not at all | 13.1 (12.4-13.8) | 15.0 (13.3-16.9) | 13.6 (12.3-14.9) | 12.0 (11.0-13.1) |
| **Watching television** |  |  |  |  |
| Yes | 9.9 (8.5-11.6) | 14.2 (5.1-33.4) | 9.9 (8.3-11.7) | 9.8 (6.9-13.7) |
| Not at all | 13.1 (12.4-13.8) | 15.3 (13.7-16.9) | 13.3 (12.2-14.5) | 12.0 (11.1-13.0) |
| **Household factors** |  |  |  |  |
| **Sex of the household head** |  |  |  |  |
| Male | 12.7 (12.1-13.4) | 14.8 (13.2-16.7) | 12.8 (11.8-13.9) | 11.7 (10.7-12.8) |
| Female | 12.5 (10.9-14.2) | 17.8 (13.5-23.1) | 10.2 (8.1-12.8) | 12.7 (10.3-15.6) |
| **Household size** |  |  |  |  |
| 1-4 | 13.2 (11.9-14.5) | 16.1 (12.8-20.1) | 14.1 (12.1-16.4) | 11.2 (9.4-13.3) |
| 5+ | 12.5 (11.8-13.3) | 15.0 (13.3-16.9) | 11.9 (10.9-13.1) | 12.1 (11.0-13.2) |
| **Sanitation facility** |  |  |  |  |
| Improved | 11.2 (8.4-14.8) | 14.8 (6.0-31.8) | 11.1 (7.3-16.4) | 10.8 (6.9-16.5) |
| Unimproved | 11.1 (10.0-12.3) | 17.6 (12.4-24.3) | 11.2 (9.6-13.0) | 10.4 (8.9-12.0) |
| Open defecation | 13.4 (12.6-14.2) | 15.0 (13.4-16.8) | 13.1 (11.9-14.4) | 12.7 (11.5-14.0) |
| **Source of drinking water** |  |  |  |  |
| Improved | 14.5 (13.4-15.8) | 15.5 (13.7-17.3) | 15.9 (13.6-18.6) | 12.1 (10.2-14.2) |
| Unimproved | 11.8 (11.1-12.6) | 13.7 (10.2-18.2) | 11.6 (10.6-12.8) | 11.7 (10.7-12.9) |
| **Time to get a water source** |  |  |  |  |
| On-premise | 13.8 (8.9-20.9) | 7.5 (0.9-41.4) | 22.3 (12.4-36.7) | 9.6 (4.6-18.9) |
| ≤ 30 min | 12.2 (11.4-13.1) | 15.1 (13.2-17.3) | 11.2 (12.4-36.7) | 11.7 (10.6-13.1) |
| 31-60 min | 11.8 (10.6-13.2) | 12.4 (9.3-16.5) | 12.1 (10.4-14.1) | 11.3 (9.5-13.4) |
| >60 min | 15.1 (13.6-16.8) | 18.4 (14.7-22.8) | 15.7 (13.4-18.3) | 13.0 (10.9-15.6) |
| **Child stool disposal** |  |  |  |  |
| Safe | 11.8 (10.3-13.4) | 12.5 (7.8-19.6) | 12.5 (10.5-14.9) | 10.7 (8.6-13.2) |
| Unsafe | 12.8 (12.1-13.5) | 15.3 (13.7-17.1) | 12.3 (11.2-13.4) | 12.1 (11.1-13.2) |
| **Community-level characteristics** |  |  |  |  |
| **Residence** |  |  |  |  |
| Urban | 13.3 (8.4-20.4) | 2.9 (0.7-5.5) | 14.7 (6.5-29.7) | 13.8 (7.8-23.4) |
| Rural | 12.7 (12.1-13.3) | 15.3 (13.7-17.0) | 12.4 (11.5-13.4) | 11.8 (10.9-12.8) |
| **Region** |  |  |  |  |
| Agrarian | 12.5 (11.7-13.4) | 17.7 (15.4-20.2) | 11.7 (10.5-13.0) | 11.2 (10.0-12.5) |
| Pastoralist | 12.9 (12.0-13.9) | 12.5 (10.5-14.8) | 13.4 (11.9-15.1) | 12.7 (11.3-14.2) |
| City administration | 12.7 (5.6-26.2) | 14.3 (2.4-52.5) | 10.8 (2.0-41.5) | 13.2 (4.2-34.1) |
