# Supplementary material for: Factors associated with childhood undernutrition in poor Ethiopian households: Implications for public health interventions
Source: PLoS One. 2025 May 9;20(5):e0323332. doi: 10.1371/journal.pone.0323332 (PMC12063910; doi:10.1371/journal.pone.0323332)
Supplement: S3 File — (DOCX) [file pone.0323332.s003.docx]

**Supplementary File 3: Prevalence of underweight among children 0-59 months in poor households with different characteristics for the survey year 2005, 2011 and 2016.**

|  | **Underweight prevalence, 95%CI** | | | |
| --- | --- | --- | --- | --- |
| **Variables** | **Pooled (EDHS 2005-2016)** | **EDHS-2005** | **EDHS-2011** | **EDHS-2016** |
| **Child factors** |  |  |  |  |
| **Sex** |  |  |  |  |
| Male | 34.4 (33.1-35.6) | 39.6 (36.6-42.7) | 36.9 (34.9-38.9) | 29.7 (27.9-31.6) |
| Female | 31.2 (30.0-32.5) | 35.7 (32.6-38.8) | 31.9 (30.1-33.9) | 28.6 (26.7-30.5) |
| **Age (months)** |  |  |  |  |
| **< 6** | 9.8 (8.2-11.8) | 11.2 (7.2-17.2) | 8.4 (6.2-11.4) | 10.7 (8.3-13.7) |
| 6-11 | 25.1 (22.6-27.7) | 30.7 (24.6-37.5) | 27.3 (23.6-31.4) | 20.1 (16.7-24.1) |
| 12-23 | 35.1 (33.1-37.2) | 39.9 (35.0-45.0) | 37.1 (33.9-40.5) | 31.1 (28.1-34.3) |
| 24-35 | 36.6 (34.5-38.7) | 38.4 (33.5-43.6) | 40.1 (36.7-43.5) | 32.8 (29.8-35.9) |
| 36-59 | 37.5 (36.1-38.9) | 43.2 (39.8-46.6) | 38.8 (36.7-41.1) | 33.5 (31.3-35.7) |
| **Size of the child at birth** |  |  |  |  |
| Larger | 27.2 (25.6-28.7) | 33.3 (29.6-37.3) | 28.2 (25.8-30.6) | 23.3 (21.1-25.7) |
| Average | 31.9 (30.6-33.4) | 35.8 (32.4-39.4) | 33.6 (31.4-35.9) | 29.0 (27.0-31.1) |
| Small | 39.7 (38.1-41.4) | 44.5 (40.5-48.5) | 41.6 (39.0-44.1) | 35.6 (33.0-38.2) |
| **Birth order** |  |  |  |  |
| First born | 32.0 (29.8-34.3) | 35.2 (29.7-41.2) | 35.0 (31.6-38.5) | 27.6 (24.4-31.0) |
| 2-4 | 34.1 (32.8-35.5) | 39.9 (36.6-43.4) | 35.8 (33.7-38.0) | 30.1 (28.1-32.1) |
| 5+ | 31.8 (30.5-33.2) | 36.4 (33.1-39.7) | 32.8 (30.7-35.0) | 28.9 (26.8-30.9) |
| **Full vaccination** |  |  |  |  |
| Yes | 32.4 (30.1-34.8) | 33.5 (27.1-40.5) | 36.1 (32.6-39.6) | 27.6 (24.1-31.3) |
| No | 32.7 (31.6-33.7) | 37.9 (35.6-40.4) | 34.3 (32.7-35.8) | 25.9 (24.1-27.9) |
| **Vitamin A last 6 months** |  |  |  |  |
| Yes | 32.8 (31.5-34.2) | 35.9 (32.8-39.1) | 35.1 (33.0-37.1) | 28.5 (26.4-30.7) |
| No | 32.9 (31.7-34.1) | 39.8 (36.7-42.9) | 33.8 (31.9-35.7) | 29.8 (28.1-31.5) |
| **Currently breastfeeding** |  |  |  |  |
| Yes | 32.5 (31.6-33.6) | 38.2 (35.7-40.8) | 33.6 (32.0-35.2) | 29.0 (27.5-30.6) |
| No | 33.7 (31.9-35.5) | 36.1 (31.8-40.6) | 37.4 (34.5-40.4) | 29.6 (27.1-32.2) |
| **Early initiation of breastfeeding** |  |  |  |  |
| Yes | 30.5 (29.2-31.9) | 35.7 (32.6-39.1) | 32.7 (30.3-35.1) | 26.9 (25.1-28.9) |
| No | 33.3 (31.5-35.1) | 37.3 (32.6-42.3) | 35.4 (33.0-37.9) | 27.2 (24.2-30.4) |
| **Birth interval** |  |  |  |  |
| 7- 33 months / short/ | 32.7 (31.6-33.7) | 38.0 (35.4-40.8) | 34.2 (32.5-35.8) | 29.1 (27.6-30.7) |
| ≥ 33 months /non-short/ | 33.2 (31.6-34.8) | 37.1 (33.4-40.8) | 35.1 (32.6-37.7) | 29.3 (26.9-31.8) |
| **Diarrhoea** |  |  |  |  |
| Yes | 40.0 (37.6-42.5) | 40.5 (35.6-45.5) | 41.7 (37.9-45.6) | 37.6 (33.6-41.9) |
| No | 31.6 (30.7-32.6) | 37.1 (34.7-39.6) | 33.2 (31.7-34.7) | 28.1 (26.7-29.5) |
| **Fever** |  |  |  |  |
| Yes | 38.2 (36.0-40.5) | 39.8 (34.9-44.9) | 41.6 (38.2-45.0) | 32.9 (29.3-36.8) |
| No | 31.8 (30.8-32.8) | 37.3 (34.8-39.7) | 32.9 (31.4-34.5) | 28.5 (27.2-30.0) |
| **Cough** |  |  |  |  |
| Yes | 34.9 (32.9-36.9) | 37.4 (32.4-42.7) | 37.8 (34.7-41.0) | 30.9 (27.9-34.0) |
| No | 32.4 (31.3-33.3) | 37.8 (35.5-40.3) | 33.6 (32.1-35.2) | 28.8 (27.3-30.3) |
| **Parental factors** |  |  |  |  |
| **Mother's age** |  |  |  |  |
| 15-17 | 29.7 (21.3-39.7) | 43.9 (21.9-68.6) | 20.2 (8.8-39.9) | 30.3 (19.4-43.9) |
| 18-24 | 30.8 (29.1-32.7) | 33.3 (29.1-37.8) | 32.3 (29.5-35.2) | 28.4 (25.7-31.1) |
| 25-34 | 34.4 (33.1-35.6) | 40.8 (37.6-44.0) | 36.4 (34.4-38.4) | 29.9 (28.1-31.9) |
| 35-49 | 31.7 (30.0-33.4) | 36.0 (32.1-40.2) | 32.9 (30.3-35.7) | 28.2 (25.6-30.9) |
| **Mother's education** |  |  |  |  |
| No education | 34.3 (33.3-35.3) | 38.8 (36.5-41.1) | 35.2 (33.7-36.8) | 31.1 (29.6-32.7) |
| Primary and above | 26.3 (24.4-28.2) | 26.8 (20.8-33.9) | 31.0 (27.9-34.3) | 22.2 (19.8-24.9) |
| **Mother's currently working** |  |  |  |  |
| Yes | 31.8 (30.1-33.6) | 34.5 (30.1-39.2) | 32.8 (30.3-35.4) | 29.3 (26.6-32.2) |
| No | 33.2 (32.2-34.2) | 38.6 (36.2-41.1) | 35.1 (33.5-36.8) | 29.1 (27.6-30.7) |
| **Maternal BMI (kg/m^2^)** |  |  |  |  |
| <18.5 | 39.8 (37.9-41.8) | 46.6 (41.9-51.3) | 42.2 (39.2-45.2) | 34.6 (31.7-37.6) |
| 18.5 to 24.9 | 31.1 (30.1-32.2) | 35.5 (33.0-38.1) | 32.4 (30.9-34.1) | 28.0 (26.5-29.6) |
| 25 + | 22.2 (18.0-27.1) | 26.5 (17.0-38.7) | 20.8 (13.7-30.2) | 21.4 (15.9-28.2) |
| **Maternal stature** |  |  |  |  |
| Very short | 45.4 (39.9-51.0) | 50.2 (37.8-62.4) | 41.9 (33.6-50.7) | 46.7 (37.9-55.6) |
| Short | 37.8 (36.3-39.3) | 42.6 (38.9-46.3) | 37.9 (35.7-40.2) | 35.4 (33.1-37.8) |
| Normal | 29.2 (28.1-30.3) | 34.0 (31.4-36.8) | 31.6 (29.8-33.5) | 25.1 (23.5-26.7) |
| **Maternal anemia** |  |  |  |  |
| Yes | 32.9 (31.2-34.6) | 33.8 (30.0-37.8) | 38.6 (35.5-41.8) | 29.3 (27.2-31.6) |
| No | 32.8 (31.7-33.8) | 38.6 (35.9-41.4) | 33.6 (32.1-35.2) | 29.2 (27.6-30.9) |
| **Place of delivery** |  |  |  |  |
| Home | 33.4 (32.5-34.4) | 37.7 (35.6-39.9) | 34.7 (33.3-36.1) | 29.8 (28.4-31.3) |
| Health facility | 26.1 (23.3-29.1) | 39.3 (23.3-57.9) | 25.2 (18.4-33.4) | 25.7 (22.7-29.0) |
| **Listening to radio** |  |  |  |  |
| Yes | 32.1 (30.4-33.9) | 28.9 (24.5-33.7) | 33.0 (30.1-35.3) | 31.7 (28.0-35.5) |
| Not at all | 33.1 (32.0-34.1) | 39.9 (37.4-42.4) | 35.3 (33.6-37.2) | 28.8 (27.4-30.2) |
| **Watching television** |  |  |  |  |
| Yes | 32.5 (30.1-34.9) | 37.7 (35.6-39.9) | 31.3 (28.7-34.0) | 36.6 (31.3-42.1) |
| Not at all | 32.9 (31.9-33.8) | 37.2 (20.9-56.9) | 35.5 (33.9-37.2) | 28.6 (27.3-30.0) |
| **Household factors** |  |  |  |  |
| **Sex of the household head** |  |  |  |  |
| Male | 32.7 (31.8-33.7) | 37.2 (34.9-39.6) | 34.7 (33.2-36.2) | 28.9 (27.4-30.3) |
| Female | 33.6 (31.3-36.0) | 40.9 (34.9-47.1) | 33.0 (29.5-36.8) | 31.3 (27.7-35.0) |
| **Household size** |  |  |  |  |
| 1-4 | 33.4 (31.6-35.2) | 35.3 (30.8-40.1) | 36.8 (33.9-39.8) | 29.4 (26.7-32.2) |
| 5+ | 32.7 (31.7-33.7) | 38.3 (35.9-40.8) | 33.7 (32.2-35.3) | 29.1 (27.6-30.6) |
| **Environmental factors** |  |  |  |  |
| **Sanitation facility** |  |  |  |  |
| Improved | 28.7 (24.4-33.5) | 45.2 (28.9-62.6) | 27.1 (21.2-33.9) | 27.5 (21.2-34.8) |
| Unimproved | 29.7 (28.1-31.4) | 37.6 (30.4-45.4) | 31.5 (29.0-34.1) | 27.3 (25.1-29.6) |
| Open defecation | 34.3 (33.2-35.3) | 37.7 (35.4-40.0) | 35.9 (34.2-37.7) | 30.5 (28.8-32.2) |
| **Source of drinking water** |  |  |  |  |
| Improved | 35.7 (34.1-37.3) | 38.5 (36.2-40.9) | 36.1 (32.9-39.5) | 31.0 (28.3-33.8) |
| Unimproved | 31.4 (30.4-32.5) | 33.7 (28.5-39.3) | 33.7 (32.2-35.3) | 28.7 (27.3-30.3) |
| **Time to get a water source** |  |  |  |  |
| On-premise | 34.1 (26.6-42.5) | 36.2 (14.8-65.1) | 38.4 (25.5-53.3) | 31.3 (22.1-42.4) |
| ≤ 30 min | 32.0 (30.8-33.2) | 36.9 (34.2-39.7) | 33.6 (31.8-35.5) | 28.2 (26.5-30.0) |
| 31-60 min | 32.5 (30.7-34.3) | 35.8 (30.8-41.1) | 33.3 (30.8-36.1) | 30.4 (27.7-33.2) |
| >60 min | 35.7 (33.7-37.8) | 42.2 (37.2-47.4) | 37.9 (34.7-41.2) | 30.5 (27.4-33.8) |
| **Child stool disposal** |  |  |  |  |
| Safe | 30.7 (28.5-32.9) | 44.6 (36.1-53.5) | 31.6 (28.6-34.8) | 27.2 (24.2-30.6) |
| Unsafe | 33.3 (32.3-34.2) | 37.3 (35.1-39.6) | 35.2 (33.7-36.8) | 29.5 (28.1-31.0) |
| **Community-level characteristics** |  |  |  |  |
| **Residence** |  |  |  |  |
| Urban | 21.3 (15.1-29.3) | 35.1 (12.7-66.7) | 20.4 (10.4-36.1) | 20.0 (12.6-30.3) |
| Rural | 32.9 (32.1-33.8) | 37.7 (35.5-39.9) | 34.6 (33.2-36.0) | 29.4 (28.0-30.7) |
| **Region** |  |  |  |  |
| Agrarian | 34.7 (33.5-35.9) | 41.5 (38.5-44.6) | 36.5 (34.7-38.4) | 29.9 (28.1-31.8) |
| Pastoralist | 30.4 (29.1-31.8) | 33.4 (30.3-36.6) | 31.5 (29.4-33.7) | 28.2 (26.3-30.2) |
| City administration | 36.5 (23.8-51.5) | 35.3 (12.0-68.5) | 35.8 (15.1-63.7) | 37.4 (20.3-58.3) |
