# Supplementary material for: Factors associated with childhood undernutrition in poor Ethiopian households: Implications for public health interventions
Source: PLoS One. 2025 May 9;20(5):e0323332. doi: 10.1371/journal.pone.0323332 (PMC12063910; doi:10.1371/journal.pone.0323332)
Supplement: S4 File — (DOCX) [file pone.0323332.s004.docx]

**Supplementary File 4 (a): Multilevel bivariable binary logistic regression analysis of factors associated with stunting, wasting and underweight in children aged 0-59 months in Ethiopia, EDHS-2005.**

| **Variables** | **Stunting** | | **Wasting** | | **Underweight** | |
| --- | --- | --- | --- | --- | --- | --- |
|  | **Crude OR, 95%CI** | **p-values** | **Crude OR, 95%CI** | **p-values** | **Crude OR, 95%CI** | **p-values** |
| ***Child factors*** |  |  |  |  |  |  |
| **Sex** |  |  |  |  |  |  |
| Male | 1.21 (0.99-1.48) | 0.057 | 1.29 (0.99-1.67) | 0.057 | 1.18 (0.95-1.45) | 0.121 |
| Female | Ref. |  | Ref. |  | Ref. |  |
| **Age (months)** |  |  |  |  |  |  |
| **< 6** | 0.12 (0.07-0.21) | p<0.001 | 2.31 (1.44-3.67) | p<0.001 | 0.27 (0.16-0.44) | p<0.001 |
| 6-11 | 0.45 (0.31-0.64) | p<0.001 | 2.10 (1.37-3.19) | 0.001 | 0.67 (0.46-0.97) | 0.033 |
| 12-23 | 0.92 (0.70-1.21) | 0.549 | 1.77 (1.25-2.51) | 0.001 | 0.92 (0.69-1.21) | 0.546 |
| 24-35 | 1.61 (1.20-2.17) | 0.001 | 1.08 (0.72-1.60) | 0.711 | 1.03 (0.78-1.38) | 0.812 |
| 36-59 | Ref. |  | Ref. |  | Ref. |  |
| **Size of the child at birth** |  |  |  |  |  |  |
| Larger | Ref. |  | Ref. |  | Ref. |  |
| Average | 1.23 (0.96-1.59) | 0.098 | 1.10 (0.79-1.54) | 0.563 | 1.13 (0.87-1.48) | 0.339 |
| Small | 1.41 (1.08-1.83) | 0.011 | 1.46 (1.04-2.04) | 0.028 | 1.51 (1.15-1.99) | 0.003 |
| **Birth order** |  |  |  |  |  |  |
| First born | Ref. |  | Ref. |  | Ref. |  |
| 2-4 | 1.13 (0.84-1.52) | 0.418 | 1.14 (0.76-1.69) | 0.513 | 1.44 (1.05-1.97) | 0.023 |
| 5+ | 1.03 (0.76-1.39) | 0.826 | 1.13 (0.75-1.68) | 0.542 | 1.28 (0.93-1.77) | 0.127 |
| **Full vaccination** |  |  |  |  |  |  |
| Yes | Ref. |  | Ref. |  | Ref. |  |
| No | 0.84 (0.59-1.20) | 0.348 | 1.64 (0.98-2.75) | 0.061 | 0.99 (0.68-1.44) | 0.958 |
| **Vitamin A last 6 months** |  |  |  |  |  |  |
| Yes | Ref. |  | Ref. |  | Ref. |  |
| No | 0.75 (0.60-0.93) | 0.010 | 1.12 (0.85-1.47) | 0.407 | 0.97 (0.78-1.22) | 0.849 |
| **Currently breastfeeding** |  |  |  |  |  |  |
| Yes | Ref. |  | Ref. |  | Ref. |  |
| No | 1.20 (0.95-1.52) | 0.118 | 0.78 (0.57-1.06) | 0.114 | 1.10 (0.86-1.39) | 0.431 |
| **Early initiation of breastfeeding** |  |  |  |  |  |  |
| Yes | Ref. |  | Ref. |  | Ref. |  |
| No | 0.90 (0.67-1.22) | 0.513 | 0.83 (0.58-1.18) | 0.314 | 0.93 (0.69-1.26) | 0.660 |
| **Birth interval** |  |  |  |  |  |  |
| 7- 33 months / short/ | 0.96 (0.77-1.19) | 0.723 | 0.89 (0.67-1.17) | 0.419 | 0.94 (0.75-1.18) | 0.631 |
| ≥ 33 months /non-short/ | Ref. |  | Ref. |  | Ref. |  |
| **Diarrhoea** |  |  |  |  |  |  |
| Yes | 1.09 (0.83-1.41) | 0.520 | 1.31 (0.95-1.81) | 0.096 | 1.30 (0.99-1.70) | 0.055 |
| No | Ref. |  | Ref. |  | Ref. |  |
| **Fever** |  |  |  |  |  |  |
| Yes | 0.96 (0.74-1.25) | 0.799 | 1.25 (0.90-1.74) | 0.180 | 1.05 (0.80-1.38) | 0.702 |
| No | Ref. |  | Ref. |  | Ref. |  |
| **Cough** |  |  |  |  |  |  |
| Yes | 0.94 (0.71-1.23) | 0.654 | 1.15 (0.81-1.62) | 0.440 | 1.03 (0.77-1.37) | 0.821 |
| No | Ref. |  | Ref. |  | Ref. |  |
| ***Parental factors*** |  |  |  |  |  |  |
| **Mother's age** |  |  |  |  |  |  |
| 15-17 | 1.35 (0.48-3.81) | 0.564 | 1.02 (0.28-3.69) | 0.973 | 0.88 (0.30-2.56) | 0.816 |
| 18-24 | 0.93 (0.69-1.24) | 0.631 | 0.98 (0.68-1.41) | 0.928 | 0.91 (0.67-1.23) | 0.533 |
| 25-34 | 1.02 (0.79-1.30) | 0.867 | 0.83 (0.61-1.14) | 0.254 | 1.04 (0.81-1.34) | 0.751 |
| 35-49 | Ref. |  | Ref. |  | Ref. |  |
| **Mother's education** |  |  |  |  |  |  |
| No education | 1.09 (0.77-1.56) | 0.604 | 1.64 (0.98-2.75) | 0.056 | 1.67 (1.13-2.46) | 0.009 |
| Primary and above | Ref. |  | Ref. |  | Ref. |  |
| **Mother's currently working** |  |  |  |  |  |  |
| Yes | 1.27 (0.97-1.65) | 0.074 | 1.13 (0.82-1.55) | 0.445 | 0.89 (0.68-1.18) | 0.434 |
| No | Ref. |  | Ref. |  | Ref. |  |
| **Maternal BMI (kg/m^2^)** |  |  |  |  |  |  |
| <18.5 | Ref. |  | Ref. |  | Ref. |  |
| 18.5 to 24.9 | 1.03 (0.81-1.29) | 0.811 | 0.63 (0.47-0.84) | 0.001 | 0.79 (0.63-1.01) | 0.058 |
| 25 + | 0.93 (0.48-1.80) | 0.825 | 0.48 (0.18-1.25) | 0.134 | 0.50 (0.24-1.04) | 0.066 |
| **Maternal stature** |  |  |  |  |  |  |
| Very short | 1.88 (0.97-3.65) | 0.061 | 0.80 (0.33-1.94) | 0.628 | 2.13 (1.11-4.07) | 0.022 |
| Short | 1.52 (1.22-1.89) | p<0.001 | 1.01 (0.77-1.34) | 0.914 | 1.45 (1.16-1.82) | 0.001 |
| Normal | Ref. |  | Ref. |  | Ref. |  |
| **Maternal anemia** |  |  |  |  |  |  |
| Yes | 0.83 (0.66-1.03) | 0.099 | 0.75 (0.56-1.01) | 0.064 | 0.91 (0.72-1.15) | 0.456 |
| No | Ref. |  | Ref. |  | Ref. |  |
| **Place of delivery** |  |  |  |  |  |  |
| Home | 0.94 (0.52-1.68) | 0.829 | 1.43 (0.63-3.24) | 0.386 | 0.91 (0.50-1.66) | 0.763 |
| Health facility | Ref. |  | Ref. |  | Ref. |  |
| **Listening to radio** |  |  |  |  |  |  |
| Yes | Ref. |  | Ref. |  | Ref. |  |
| Not at all | 1.05 (0.81-1.38) | 0.694 | 1.15 (0.81-1.63) | 0.434 | 1.39 (1.05-1.85) | 0.022 |
| **Watching television** |  |  |  |  |  |  |
| Yes | Ref. |  | Ref. |  | Ref. |  |
| Not at all | 0.99 (0.36-2.67) | 0.994 | 0.66 (0.21-2.08) | 0.481 | 0.66 (0.21-2.08) | 0.481 |
| ***Household factors*** |  |  |  |  |  |  |
| **Sex of the household head** |  |  |  |  |  |  |
| Male | Ref. |  | Ref. |  | Ref. |  |
| Female | 0.90 (0.68-1.20) | 0.494 | 1.02 (0.71-1.48) | 0.922 | 1.18 (0.88-1.58) | 0.263 |
| **Household size** |  |  |  |  |  |  |
| 1-4 | 1.02 (0.79-1.29) | 0.883 | 0.98 (0.72-1.35) | 0.942 | 0.87 (0.68-1.12) | 0.284 |
| 5+ | Ref. |  | Ref. |  | Ref. |  |
| ***Environmental factors*** |  |  |  |  |  |  |
| **Sanitation facility** |  |  |  |  |  |  |
| Improved | Ref. |  | Ref. |  | Ref. |  |
| Unimproved | 0.77 (0.26-2.27) | 0.633 | 1.96 (0.41-9.25) | 0.395 | 1.03 (0.34-3.08) | 0.961 |
| Open defecation | 0.55 (0.20-1.54) | 0.262 | 1.64 (0.37-7.28) | 0.511 | 1.11 (0.39-3.10) | 0.846 |
| **Source of drinking water** |  |  |  |  |  |  |
| Improved | Ref. |  | Ref. |  | Ref. |  |
| Unimproved | 0.88 (0.67-1.15) | 0.351 | 0.87 (0.62-1.23) | 0.447 | 0.85 (0.64-1.13) | 0.275 |
| **Time to get a water source** |  |  |  |  |  |  |
| On-premise | Ref. |  | Ref. |  | Ref. |  |
| ≤ 30 min | 1.48 (0.43-5.01) | 0.530 | 0.97 (0.21-4.56) | 0.971 | 1.84 (0.46-7.31) | 0.383 |
| 31-60 min | 1.85 (0.54-6.38) | 0.326 | 0.73 (0.15-3.52) | 0.699 | 1.88 (0.47-7.55) | 0.373 |
| >60 min | 1.27 (0.37-4.35) | 0.698 | 1.29 (0.27-6.11) | 0.744 | 2.19 (0.54-8.73) | 0.266 |
| **Child stool disposal** |  |  |  |  |  |  |
| Safe | Ref. |  | Ref. |  | Ref. |  |
| Unsafe | 0.84 (0.56-1.27) | 0.426 | 1.14 (0.66-1.97) | 0.618 | 0.77 (0.51-1.17) | 0.225 |
| ***Community-level characteristics*** |  |  |  |  |  |  |
| **Residence** |  |  |  |  |  |  |
| Urban | 0.69 (0.19-2.56) | 0.585 | 1.14 (0.23-5.42) | 0.871 | 0.57 (0.13-2.42) | 0.446 |
| Rural | Ref. |  | Ref. |  | Ref. |  |
| **Region** |  |  |  |  |  |  |
| Agrarian | 1.15 (0.68-1.94) | 0.597 | 1.12 (0.63-1.99) | 0.693 | 1.14 (0.66-1.96) | 0.616 |
| Pastoralist | 0.91 (0.53-1.57) | 0.753 | 0.86 (0.46-1.57) | 0.620 | 0.90 (0.51-1.58) | 0.725 |
| City administration | Ref. |  | Ref. |  | Ref. |  |

# Supplementary File 4 (b): Multilevel multivariable binary logistic regression analysis of factors associated with stunting in children aged 0-59 months in Ethiopia, EDHS-2005

|  | **Stunting** | **Model 1** | **Model 2** | **Model 3** | **Model 4** |
| --- | --- | --- | --- | --- | --- |
|  | **Crude OR, 95%CI** |  | **AOR, 95%CI** | **AOR, 95%CI** | **AOR, 95%CI** |
| **Variables** |  |  |  |  |  |
| ***Child factors*** |  |  |  |  |  |
| **Sex** |  |  |  |  |  |
| Male | 1.21 (0.99-1.48) |  | 1.29 (1.03-1.62)* |  | 1.29 (1.04-1.62)* |
| Female | Ref. |  | Ref. |  | Ref. |
| **Age (months)** |  |  |  |  |  |
| **< 6** | 0.12 (0.07-0.21) |  | 0.10 (0.06-0.18)** |  | 0.10 (0.06-0.18)** |
| 6-11 | 0.45 (0.31-0.64) |  | 0.36 (0.24-0.55)** |  | 0.36 (0.24-0.55)** |
| 12-23 | 0.92 (0.70-1.21) |  | 0.76 (0.56-1.03) |  | 0.76 (0.24-0.55)* |
| 24-35 | 1.61 (1.20-2.17) |  | 1.55 (1.13-2.12)* |  | 1.56 (1.14-2.14)* |
| 36-59 | Ref. |  | Ref. |  | Ref. |
| **Size of the child at birth** |  |  |  |  |  |
| Larger | Ref. |  | Ref. |  | Ref. |
| Average | 1.23 (0.96-1.59) |  | 1.35 (1.03-1.79)* |  | 1.35 (1.03-1.79)* |
| Small | 1.41 (1.08-1.83) |  | 1.67 (1.24-2.24)* |  | 1.67 (1.24-2.24)* |
| **Birth order** |  |  |  |  |  |
| First born | Ref. |  |  |  |  |
| 2-4 | 1.13 (0.84-1.52) |  |  |  |  |
| 5+ | 1.03 (0.76-1.39) |  |  |  |  |
| **Full vaccination** |  |  |  |  |  |
| Yes | Ref. |  |  |  |  |
| No | 0.84 (0.59-1.20) |  |  |  |  |
| **Vitamin A last 6 months** |  |  |  |  |  |
| Yes | Ref. |  | Ref. |  | Ref. |
| No | 0.75 (0.60-0.93) |  | 0.84 (0.66-1.07) |  | 0.84 (0.66-1.07) |
| **Currently breastfeeding** |  |  |  |  |  |
| Yes | Ref. |  | Ref. |  | Ref. |
| No | 1.20 (0.95-1.52) |  | 0.83 (0.63-1.09) |  | 0.83 (0.63-1.09) |
| **Early initiation of breastfeeding** |  |  |  |  |  |
| Yes | Ref. |  |  |  |  |
| No | 0.90 (0.67-1.22) |  |  |  |  |
| **Birth interval** |  |  |  |  |  |
| 7- 33 months / short/ | 0.96 (0.77-1.19) |  |  |  |  |
| ≥ 33 months /non-short/ | Ref. |  |  |  |  |
| **Diarrhoea** |  |  |  |  |  |
| Yes | 1.09 (0.83-1.41) |  |  |  |  |
| No | Ref. |  |  |  |  |
| **Fever** |  |  |  |  |  |
| Yes | 0.96 (0.74-1.25) |  |  |  |  |
| No | Ref. |  |  |  |  |
| **Cough** |  |  |  |  |  |
| Yes | 0.94 (0.71-1.23) |  |  |  |  |
| No | Ref. |  |  |  |  |
| ***Parental factors*** |  |  |  |  |  |
| **Mother's age** |  |  |  |  |  |
| 15-17 | 1.35 (0.48-3.81) |  |  |  |  |
| 18-24 | 0.93 (0.69-1.24) |  |  |  |  |
| 25-34 | 1.02 (0.79-1.30) |  |  |  |  |
| 35-49 | Ref. |  |  |  |  |
| **Mother's education** |  |  |  |  |  |
| No education | 1.09 (0.77-1.56) |  |  |  |  |
| Primary and above | Ref. |  |  |  |  |
| **Mother's currently working** |  |  |  |  |  |
| Yes | 1.27 (0.97-1.65) |  | 1.16 (0.87-1.55) |  | 1.15 (0.86-1.53) |
| No | Ref. |  | Ref. |  | Ref. |
| **Maternal BMI (kg/m^2^)** |  |  |  |  |  |
| <18.5 | Ref. |  |  |  |  |
| 18.5 to 24.9 | 1.03 (0.81-1.29) |  |  |  |  |
| 25 + | 0.93 (0.48-1.80) |  |  |  |  |
| **Maternal stature** |  |  |  |  |  |
| Very short | 1.88 (0.97-3.65) |  | 2.30 (1.11-4.78)* |  | 2.29 (1.10-4.76)* |
| Short | 1.52 (1.22-1.89) |  | 1.56 (1.22-1.99)** |  | 1.57 (1.23-2.01)** |
| Normal | Ref. |  | Ref. |  | Ref. |
| **Maternal anemia** |  |  |  |  |  |
| Yes | 0.83 (0.66-1.03) |  | 0.91 (0.71-1.15) |  | 0.90 (0.71-1.15) |
| No | Ref. |  | Ref. |  | Ref. |
| **Place of delivery** |  |  |  |  |  |
| Home | 0.94 (0.52-1.68) |  |  |  |  |
| Health facility | Ref. |  |  |  |  |
| **Listening to radio** |  |  |  |  |  |
| Yes | Ref. |  |  |  |  |
| Not at all | 1.05 (0.81-1.38) |  |  |  |  |
| **Watching television** |  |  |  |  |  |
| Yes | Ref. |  |  |  |  |
| Not at all | 0.99 (0.36-2.67) |  |  |  |  |
| ***Household factors*** |  |  |  |  |  |
| **Sex of the household head** |  |  |  |  |  |
| Male | Ref. |  |  |  |  |
| Female | 0.90 (0.68-1.20) |  |  |  |  |
| **Household size** |  |  |  |  |  |
| 1-4 | 1.02 (0.79-1.29) |  |  |  |  |
| 5+ | Ref. |  |  |  |  |
| ***Environmental factors*** |  |  |  |  |  |
| **Sanitation facility** |  |  |  |  |  |
| Improved | Ref. |  |  |  |  |
| Unimproved | 0.77 (0.26-2.27) |  |  |  |  |
| Open defecation | 0.55 (0.20-1.54) |  |  |  |  |
| **Source of drinking water** |  |  |  |  |  |
| Improved | Ref. |  |  |  |  |
| Unimproved | 0.88 (0.67-1.15) |  |  |  |  |
| **Time to get a water source** |  |  |  |  |  |
| On-premise | Ref. |  |  |  |  |
| ≤ 30 min | 1.48 (0.43-5.01) |  |  |  |  |
| 31-60 min | 1.85 (0.54-6.38) |  |  |  |  |
| >60 min | 1.27 (0.37-4.35) |  |  |  |  |
| **Child stool disposal** |  |  |  |  |  |
| Safe | Ref. |  |  |  |  |
| Unsafe | 0.84 (0.56-1.27) |  |  |  |  |
| ***Community-level characteristics*** |  |  |  |  |  |
| **Residence** |  |  |  |  |  |
| Urban | 0.69 (0.19-2.56) |  |  | 0.69 (0.19-2.57) | 2.79 (0.43-18.02) |
| Rural | Ref. |  |  | Ref. | Ref. |
| **Region** |  |  |  |  |  |
| Agrarian | 1.15 (0.68-1.94) |  |  | 1.15 (0.68-1.94) | 1.08 (0.60-1.95) |
| Pastoralist | 0.91 (0.53-1.57) |  |  | 0.92 (0.53-1.58) | 0.88 (0.47-1.61) |
| City administration | Ref. |  |  | Ref. |  |
| **Random effects** |  |  |  |  |  |
| **Variance (SD)** |  | 0.2689 (0.0096) | 0.2758 (0.0123) | 0.2691 (0.0094) | 0.2696 (0.0121) |
| **ICC (%)** |  | 7.56 | 7.73 | 7.56 | 7.57 |
| **AIC** |  | 2341.34 | 1987.66 | 2343.93 | 1990.10 |
| **BIC** |  | 2352.21 | 2067.72 | 2371.11 | 2086.18 |
| **LL** |  | -1168.67 | -978.83 | -1166.96 | -977.05 |
| **Deviance** |  | 2,337.34 | 1,957.66 | 2,333.93 | 1,954.10 |

# Supplementary File 4 (c): Multilevel multivariable binary logistic regression analysis of factors associated with wasting in children aged 0-59 months in Ethiopia, EDHS-2005

|  | **Wasting** | **Model 1** | **Model 2** | **Model 3** | **Model 4** |
| --- | --- | --- | --- | --- | --- |
|  | **Crude OR, 95%CI** |  | **AOR, 95%CI** | **AOR, 95%CI** | **AOR, 95%CI** |
| **Variables** |  |  |  |  |  |
| ***Child factors*** |  |  |  |  |  |
| **Sex** |  |  |  |  |  |
| Male | 1.29 (0.99-1.67) |  | 1.42 (1.05-1.91)* |  | 1.43 (1.06-1.93)* |
| Female | Ref. |  | Ref. |  | Ref. |
| **Age (months)** |  |  |  |  |  |
| **< 6** | 2.31 (1.44-3.67) |  | 3.02 (1.77-5.16)** |  | 3.07 (1.80-5.24)** |
| 6-11 | 2.10 (1.37-3.19) |  | 2.62 (1.59-4.30)** |  | 2.64 (1.61-4.33)** |
| 12-23 | 1.77 (1.25-2.51) |  | 2.27 (1.51-3.43)** |  | 2.28 (1.51-3.45)** |
| 24-35 | 1.08 (0.72-1.60) |  | 1.12 (0.71-1.77) |  | 1.14 (0.72-1.79) |
| 36-59 | Ref. |  | Ref. |  | Ref. |
| **Size of the child at birth** |  |  |  |  |  |
| Larger | Ref. |  | Ref. |  | Ref. |
| Average | 1.10 (0.79-1.54) |  | 1.06 (0.73-1.55) |  | 1.07 (0.73-1.56) |
| Small | 1.46 (1.04-2.04) |  | 1.52 (1.04-2.22)* |  | 1.53 (1.05-2.23)* |
| **Birth order** |  |  |  |  |  |
| First born | Ref. |  |  |  |  |
| 2-4 | 1.14 (0.76-1.69) |  |  |  |  |
| 5+ | 1.13 (0.75-1.68) |  |  |  |  |
| **Full vaccination** |  |  |  |  |  |
| Yes | Ref. |  | Ref. |  | Ref. |
| No | 1.64 (0.98-2.75) |  | 1.57 (0.90-2.74) |  | 1.65 (0.94-2.88) |
| **Vitamin A last 6 months** |  |  |  |  |  |
| Yes | Ref. |  |  |  |  |
| No | 1.12 (0.85-1.47) |  |  |  |  |
| **Currently breastfeeding** |  |  |  |  |  |
| Yes | Ref. |  | Ref. |  | Ref. |
| No | 0.78 (0.57-1.06) |  | 1.28 (0.87-1.89) |  | 1.28 (0.87-1.89) |
| **Early initiation of breastfeeding** |  |  |  |  |  |
| Yes | Ref. |  |  |  |  |
| No | 0.83 (0.58-1.18) |  |  |  |  |
| **Birth interval** |  |  |  |  |  |
| 7- 33 months / short/ | 0.89 (0.67-1.17) |  |  |  |  |
| ≥ 33 months /non-short/ | Ref. |  |  |  |  |
| **Diarrhoea** |  |  |  |  |  |
| Yes | 1.31 (0.95-1.81) |  | 1.20 (0.83-1.73) |  | 1.17 (0.82-1.69) |
| No | Ref. |  | Ref. |  | Ref. |
| **Fever** |  |  |  |  |  |
| Yes | 1.25 (0.90-1.74) |  |  |  |  |
| No | Ref. |  |  |  |  |
| **Cough** |  |  |  |  |  |
| Yes | 1.15 (0.81-1.62) |  |  |  |  |
| No | Ref. |  |  |  |  |
| ***Parental factors*** |  |  |  |  |  |
| **Mother's age** |  |  |  |  |  |
| 15-17 | 1.02 (0.28-3.69) |  |  |  |  |
| 18-24 | 0.98 (0.68-1.41) |  |  |  |  |
| 25-34 | 0.83 (0.61-1.14) |  |  |  |  |
| 35-49 | Ref. |  |  |  |  |
| **Mother's education** |  |  |  |  |  |
| No education | 1.64 (0.98-2.75) |  | 1.53 (0.89-2.64) |  | 1.56 (0.91-2.69) |
| Primary and above | Ref. |  | Ref. |  | Ref. |
| **Mother's currently working** |  |  |  |  |  |
| Yes | 1.13 (0.82-1.55) |  |  |  |  |
| No | Ref. |  |  |  |  |
| **Maternal BMI (kg/m^2^)** |  |  |  |  |  |
| <18.5 | Ref. |  | Ref. |  | Ref. |
| 18.5 to 24.9 | 0.63 (0.47-0.84) |  | 0.65 (0.47-0.91)* |  | 0.68 (0.49-0.94)* |
| 25 + | 0.48 (0.18-1.25) |  | 0.56 (0.21-1.55) |  | 0.61 (0.22-1.67) |
| **Maternal stature** |  |  |  |  |  |
| Very short | 0.80 (0.33-1.94) |  |  |  |  |
| Short | 1.01 (0.77-1.34) |  |  |  |  |
| Normal | Ref. |  |  |  |  |
| **Maternal anemia** |  |  |  |  |  |
| Yes | 0.75 (0.56-1.01) |  | 0.75 (0.54-1.03) |  | 0.74 (0.54-1.02) |
| No | Ref. |  | Ref. |  | Ref. |
| **Place of delivery** |  |  |  |  |  |
| Home | 1.43 (0.63-3.24) |  |  |  |  |
| Health facility | Ref. |  |  |  |  |
| **Listening to radio** |  |  |  |  |  |
| Yes | Ref. |  |  |  |  |
| Not at all | 1.15 (0.81-1.63) |  |  |  |  |
| **Watching television** |  |  |  |  |  |
| Yes | Ref. |  |  |  |  |
| Not at all | 0.66 (0.21-2.08) |  |  |  |  |
| ***Household factors*** |  |  |  |  |  |
| **Sex of the household head** |  |  |  |  |  |
| Male | Ref. |  |  |  |  |
| Female | 1.02 (0.71-1.48) |  |  |  |  |
| **Household size** |  |  |  |  |  |
| 1-4 | 0.98 (0.72-1.35) |  |  |  |  |
| 5+ | Ref. |  |  |  |  |
| ***Environmental factors*** |  |  |  |  |  |
| **Sanitation facility** |  |  |  |  |  |
| Improved | Ref. |  |  |  |  |
| Unimproved | 1.96 (0.41-9.25) |  |  |  |  |
| Open defecation | 1.64 (0.37-7.28) |  |  |  |  |
| **Source of drinking water** |  |  |  |  |  |
| Improved | Ref. |  |  |  |  |
| Unimproved | 0.87 (0.62-1.23) |  |  |  |  |
| **Time to get a water source** |  |  |  |  |  |
| On-premise | Ref. |  |  |  |  |
| ≤ 30 min | 0.97 (0.21-4.56) |  |  |  |  |
| 31-60 min | 0.73 (0.15-3.52) |  |  |  |  |
| >60 min | 1.29 (0.27-6.11) |  |  |  |  |
| **Child stool disposal** |  |  |  |  |  |
| Safe | Ref. |  |  |  |  |
| Unsafe | 1.14 (0.66-1.97) |  |  |  |  |
| ***Community-level characteristics*** |  |  |  |  |  |
| **Residence** |  |  |  |  |  |
| Urban | 1.14 (0.23-5.42) |  |  | 1.14 (0.24-5.42) | 1.92 (0.30-12.19) |
| Rural | Ref. |  |  | Ref. | Ref. |
| **Region** |  |  |  |  |  |
| Agrarian | 1.12 (0.63-1.99) |  |  | 1.12 (0.63-1.99) | 0.92 (0.47-1.82) |
| Pastoralist | 0.86 (0.46-1.57) |  |  | 0.85 (0.46-1.57) | 0.68 (0.34-1.40) |
| City administration | Ref. |  |  | Ref. | Ref. |
| **Random effects** |  |  |  |  |  |
| **Variance (SD)** |  | 0.0667 (0.0514) | 0.1720 (0.0390) | 0.0521 (0.0619) | 0.1426 (0.0449) |
| **ICC (%)** |  | 11.98 | 4.97 |  | 4.15 |
| **AIC** |  | 1517.18 | 1252.55 | 1519.92 | 1255.11 |
| **BIC** |  | 1528.05 | 1336.88 | 1547.10 | 1355.26 |
| **LL** |  | -756.59 | -610.27 | -754.96 | -608.55 |
| **Deviance** |  | 1,513.18 | 1,220.55 | 1,509.92 | 1,217.11 |

# Supplementary File 4 (d): Multilevel multivariable binary logistic regression analysis of factors associated with underweight in children aged 0-59 months in Ethiopia, EDHS-2005

|  | **Underweight** | **Model 1** | **Model 2** | **Model 3** | **Model 4** |
| --- | --- | --- | --- | --- | --- |
|  | **Crude OR, 95%CI** |  | **AOR, 95%CI** | **AOR, 95%CI** | **AOR, 95%CI** |
| **Variables** |  |  |  |  |  |
| ***Child factors*** |  |  |  |  |  |
| **Sex** |  |  |  |  |  |
| Male | 1.18 (0.95-1.45) |  | 1.23 (0.99-1.53) |  | 1.24 (1.01-1.54)* |
| Female | Ref. |  | Ref. |  | Ref. |
| **Age (months)** |  |  |  |  |  |
| **< 6** | 0.27 (0.16-0.44) |  | 0.24 (0.15-0.41)** |  | 0.25 (0.15-0.42)** |
| 6-11 | 0.67 (0.46-0.97) |  | 0.60 (0.41-0.89)* |  | 0.61 (0.41-0.89)* |
| 12-23 | 0.92 (0.69-1.21) |  | 0.84 (0.63-1.12) |  | 0.84 (0.63-1.12) |
| 24-35 | 1.03 (0.78-1.38) |  | 0.98 (0.73-1.32) |  | 0.99 (0.74-1.33) |
| 36-59 | Ref. |  | Ref. |  |  |
| **Size of the child at birth** |  |  |  |  |  |
| Larger | Ref. |  | Ref. |  | Ref. |
| Average | 1.13 (0.87-1.48) |  | 1.17 (0.89-1.52) |  | 1.16 (0.89-1.52) |
| Small | 1.51 (1.15-1.99) |  | 1.65 (1.25-2.19)** |  | 1.64 (1.24-2.18)** |
| **Birth order** |  |  |  |  |  |
| First born | Ref. |  | Ref. |  | Ref. |
| 2-4 | 1.44 (1.05-1.97) |  | 1.36 (0.98-1.88) |  | 1.36 (0.98-1.87) |
| 5+ | 1.28 (0.93-1.77) |  | 1.21 (0.87-1.69) |  | 1.22 (0.88-1.71) |
| **Full vaccination** |  |  |  |  |  |
| Yes | Ref. |  |  |  |  |
| No | 0.99 (0.68-1.44) |  |  |  |  |
| **Vitamin A last 6 months** |  |  |  |  |  |
| Yes | Ref. |  |  |  |  |
| No | 0.97 (0.78-1.22) |  |  |  |  |
| **Currently breastfeeding** |  |  |  |  |  |
| Yes | Ref. |  |  |  |  |
| No | 1.10 (0.86-1.39) |  |  |  |  |
| **Early initiation of breastfeeding** |  |  |  |  |  |
| Yes | Ref. |  |  |  |  |
| No | 0.93 (0.69-1.26) |  |  |  |  |
| **Birth interval** |  |  |  |  |  |
| 7- 33 months / short/ | 0.94 (0.75-1.18) |  |  |  |  |
| ≥ 33 months /non-short/ | Ref. |  |  |  |  |
| **Diarrhoea** |  |  |  |  |  |
| Yes | 1.30 (0.99-1.70) |  | 1.28 (0.97-1.71) |  | 1.27 (0.96-1.69) |
| No | Ref. |  | Ref. |  | Ref. |
| **Fever** |  |  |  |  |  |
| Yes | 1.05 (0.80-1.38) |  |  |  |  |
| No | Ref. |  |  |  |  |
| **Cough** |  |  |  |  |  |
| Yes | 1.03 (0.77-1.37) |  |  |  |  |
| No | Ref. |  |  |  |  |
| ***Parental factors*** |  |  |  |  |  |
| **Mother's age** |  |  |  |  |  |
| 15-17 | 0.88 (0.30-2.56) |  |  |  |  |
| 18-24 | 0.91 (0.67-1.23) |  |  |  |  |
| 25-34 | 1.04 (0.81-1.34) |  |  |  |  |
| 35-49 | Ref. |  |  |  |  |
| **Mother's education** |  |  |  |  |  |
| No education | 1.67 (1.13-2.46) |  | 1.55 (1.04-2.33)* |  | 1.57 (1.05-2.35)* |
| Primary and above | Ref. |  | Ref. |  | Ref. |
| **Mother's currently working** |  |  |  |  |  |
| Yes | 0.89 (0.68-1.18) |  |  |  |  |
| No | Ref. |  |  |  |  |
| **Maternal BMI (kg/m^2^)** |  |  |  |  |  |
| <18.5 | Ref. |  | Ref. |  | Ref. |
| 18.5 to 24.9 | 0.79 (0.63-1.01) |  | 0.75 (0.59-0.96)* |  | 0.76 (0.60-0.97)* |
| 25 + | 0.50 (0.24-1.04) |  | 0.47 (0.22-1.01) |  | 0.48 (0.22-1.03) |
| **Maternal stature** |  |  |  |  |  |
| Very short | 2.13 (1.11-4.07) |  | 2.39 (1.21-4.71)* |  | 2.36 (1.19-4.65)* |
| Short | 1.45 (1.16-1.82) |  | 1.51 (1.20-1.91)** |  | 1.52 (1.20-1.91)** |
| Normal | Ref. |  | Ref. |  | Ref. |
| **Maternal anemia** |  |  |  |  |  |
| Yes | 0.91 (0.72-1.15) |  |  |  |  |
| No | Ref. |  |  |  |  |
| **Place of delivery** |  |  |  |  |  |
| Home | 0.91 (0.50-1.66) |  |  |  |  |
| Health facility | Ref. |  |  |  |  |
| **Listening to radio** |  |  |  |  |  |
| Yes | Ref. |  | Ref. |  | Ref. |
| Not at all | 1.39 (1.05-1.85) |  | 1.32 (0.98-1.77) |  | 1.29 (0.96-1.74) |
| **Watching television** |  |  |  |  |  |
| Yes | Ref. |  |  |  |  |
| Not at all | 0.66 (0.21-2.08) |  |  |  |  |
| ***Household factors*** |  |  |  |  |  |
| **Sex of the household head** |  |  |  |  |  |
| Male | Ref. |  |  |  |  |
| Female | 1.18 (0.88-1.58) |  |  |  |  |
| **Household size** |  |  |  |  |  |
| 1-4 | 0.87 (0.68-1.12) |  |  |  |  |
| 5+ | Ref. |  |  |  |  |
| ***Environmental factors*** |  |  |  |  |  |
| **Sanitation facility** |  |  |  |  |  |
| Improved | Ref. |  |  |  |  |
| Unimproved | 1.03 (0.34-3.08) |  |  |  |  |
| Open defecation | 1.11 (0.39-3.10) |  |  |  |  |
| **Source of drinking water** |  |  |  |  |  |
| Improved | Ref. |  |  |  |  |
| Unimproved | 0.85 (0.64-1.13) |  |  |  |  |
| **Time to get a water source** |  |  |  |  |  |
| On-premise | Ref. |  |  |  |  |
| ≤ 30 min | 1.84 (0.46-7.31) |  |  |  |  |
| 31-60 min | 1.88 (0.47-7.55) |  |  |  |  |
| >60 min | 2.19 (0.54-8.73) |  |  |  |  |
| **Child stool disposal** |  |  |  |  |  |
| Safe | Ref. |  |  |  |  |
| Unsafe | 0.77 (0.51-1.17) |  |  |  |  |
| ***Community-level characteristics*** |  |  |  |  |  |
| **Residence** |  |  |  |  |  |
| Urban | 0.57 (0.13-2.42) |  |  | 0.57 (0.13-2.42) | 0.73 (0.17-3.15) |
| Rural | Ref. |  |  | Ref. | Ref. |
| **Region** |  |  |  |  |  |
| Agrarian | 1.14 (0.66-1.96) |  |  | 1.15 (0.67-1.97) | 1.03 (0.60-1.78) |
| Pastoralist | 0.90 (0.51-1.58) |  |  | 0.91 (0.52-1.59) | 0.86 (0.48-1.51) |
| City administration | Ref. |  |  | Ref. | Ref. |
| **Random effects** |  |  |  |  |  |
| **Variance (SD)** |  | 0.2941 (0.0096) | 0.2605 (0.0106) | 0.2818 (0.0097) | 0.2515 (0.0107) |
| **ICC (%)** |  | 8.20 | 7.33 | 7.89 | 7.10 |
| **AIC** |  | 2240.11 | 2165.01 | 2242.34 | 2168.95 |
| **BIC** |  | 2250.98 | 2262.75 | 2269.52 | 2282.98 |
| **LL** |  | -1118.05 | -1064.50 | -1116.17 | -1063.47 |
| **Deviance** |  | 2,236.10 | 2,129.01 | 2,232.34 | 2,126.95 |
