# Supplementary material for: Factors associated with childhood undernutrition in poor Ethiopian households: Implications for public health interventions
Source: PLoS One. 2025 May 9;20(5):e0323332. doi: 10.1371/journal.pone.0323332 (PMC12063910; doi:10.1371/journal.pone.0323332)
Supplement: S5 File — Multilevel bivariable binary logistic regression analysis of factors associated with stunting, wasting and underweight in children aged 0–59 months in Ethiopia, EDHS-2011. (DOCX) [file pone.0323332.s005.docx]

**Supplementary File 5 (a): Multilevel bivariable binary logistic regression analysis of factors associated with stunting, wasting and underweight in children aged 0-59 months in Ethiopia, EDHS-2011.**

|  | **Stunting** |  | **Wasting** |  | **Underweight** |  |
| --- | --- | --- | --- | --- | --- | --- |
|  | **Crude OR, 95%CI** | **p-values** | **Crude OR, 95%CI** | **p-values** | **Crude OR, 95%CI** | **p-values** |
| **Variables** |  |  |  |  |  |  |
| ***Child factors*** |  |  |  |  |  |  |
| **Sex** |  |  |  |  |  |  |
| Male | 1.13 (1.01-1.27) | 0.047 | 1.45 (1.22-1.72) | p<0.001 | 1.27 (1.12-1.44) | p<0.001 |
| Female | Ref. |  | Ref. |  | Ref. |  |
| **Age (months)** |  |  |  |  |  |  |
| **< 6** | 0.08 (0.06-0.11) | p<0.001 | 1.86 (1.40-2.47) | p<0.001 | 0.17 (0.13-0.23) | p<0.001 |
| 6-11 | 0.21 (0.16-0.27) | p<0.001 | 2.73 (2.07-3.59) | p<0.001 | 0.54 (0.43-0.68) | p<0.001 |
| 12-23 | 0.82 (0.69-0.97) | 0.027 | 2.52 (2.01-3.17) | p<0.001 | 1.01 (0.85-1.20) | 0.897 |
| 24-35 | 1.34 (1.13-1.59) | 0.001 | 1.19 (0.92-1.54) | 0.173 | 1.25 (1.05-1.48) | 0.009 |
| 36-59 | Ref. |  | Ref. |  | Ref. |  |
| **Size of the child at birth** |  |  |  |  |  |  |
| Larger | Ref. |  | Ref. |  | Ref. |  |
| Average | 1.16 (0.99-1.36) | 0.057 | 1.08 (0.85-1.37) | 0.504 | 1.27 (1.07-1.49) | 0.005 |
| Small | 1.15 (0.98-1.35) | 0.075 | 1.82 (1.45-2.27) | p<0.001 | 1.68 (1.42-1.98) | p<0.001 |
| **Birth order** |  |  |  |  |  |  |
| First born | Ref. |  | Ref. |  | Ref. |  |
| 2-4 | 1.02 (0.86-1.22) | 0.784 | 1.06 (0.82-1.37) | 0.669 | 1.16 (0.97-1.40) | 0.107 |
| 5+ | 1.10 (0.92-1.32) | 0.295 | 1.32 (1.02-1.70) | 0.034 | 1.27 (1.05-1.54) | 0.011 |
| **Full vaccination** |  |  |  |  |  |  |
| Yes | Ref. |  | Ref. |  | Ref. |  |
| No | 0.61 (0.51-0.73) | p<0.001 | 1.47 (1.13-1.92) | 0.005 | 0.83 (0.69-0.99) | 0.041 |
| **Vitamin A last 6 months** |  |  |  |  |  |  |
| Yes | Ref. |  | Ref. |  | Ref. |  |
| No | 0.63 (0.56-0.72) | p<0.001 | 1.28 (1.07-1.53) | 0.006 | 0.89 (0.79-1.02) | 0.118 |
| **Currently breastfeeding** |  |  |  |  |  |  |
| Yes | Ref. |  | Ref. |  | Ref. |  |
| No | 1.67 (1.45-1.92) | p<0.001 | 0.74 (0.61-0.91) | 0.004 | 1.27 (1.10-1.46) | 0.001 |
| **Early initiation of breastfeeding** |  |  |  |  |  |  |
| Yes | Ref. |  | Ref. |  | Ref. |  |
| No | 1.05 (0.89-1.22) | 0.538 | 0.91 (0.74-1.11) | 0.359 | 1.02 (0.87-1.19) | 0.788 |
| **Birth interval** |  |  |  |  |  |  |
| 7- 33 months / short/ | 0.91 (0.80-1.04) | 0.172 | 1.07 (0.89-1.29) | 0.439 | 0.92 (0.80-1.05) | 0.230 |
| ≥ 33 months /non-short/ | Ref. |  | Ref. |  | Ref. |  |
| **Diarrhoea** |  |  |  |  |  |  |
| Yes | 1.11 (0.94-1.32) | 0.196 | 2.04 (1.65-2.50) | p<0.001 | 1.58 (1.33-1.88) | p<0.001 |
| No | Ref. |  | Ref. |  | Ref. |  |
| **Fever** |  |  |  |  |  |  |
| Yes | 1.01 (0.87-1.18) | 0.839 | 1.84 (1.51-2.23) | p<0.001 | 1.46 (1.25-1.71) | p<0.001 |
| No | Ref. |  | Ref. |  | Ref. |  |
| **Cough** |  |  |  |  |  |  |
| Yes | 1.05 (0.90-1.22) | 0.538 | 1.22 (0.99-1.50) | 0.051 | 1.19 (1.02-1.39) | 0.025 |
| No | Ref. |  | Ref. |  | Ref. |  |
| ***Parental factors*** |  |  |  |  |  |  |
| **Mother's age** |  |  |  |  |  |  |
| 15-17 | 0.26 (0.12-0.57) | 0.001 | 1.25 (0.53-2.97) | 0.610 | 0.28 (0.12-0.67) | 0.004 |
| 18-24 | 0.85 (0.71-1.02) | 0.083 | 1.06 (0.83-1.35) | 0.638 | 0.82 (0.68-0.98) | 0.037 |
| 25-34 | 0.97 (0.84-1.13) | 0.753 | 1.04 (0.84-1.27) | 0.727 | 1.06 (0.91-1.24) | 0.407 |
| 35-49 | Ref. |  | Ref. |  | Ref. |  |
| **Mother's education** |  |  |  |  |  |  |
| No education | 1.23 (1.04-1.45) | 0.013 | 1.27 (0.99-1.61) | 0.052 | 1.52 (1.27-1.82) | p<0.001 |
| Primary and above | Ref. |  | Ref. |  | Ref. |  |
| **Mother's currently working** |  |  |  |  |  |  |
| Yes | 1.04 (0.90-1.21) | 0.557 | 0.72 (0.58-0.90) | 0.004 | 0.97 (0.83-1.13) | 0.699 |
| No | Ref. |  | Ref. |  | Ref. |  |
| **Maternal BMI (kg/m^2^)** |  |  |  |  |  |  |
| <18.5 | Ref. |  | Ref. |  | Ref. |  |
| 18.5 to 24.9 | 0.92 (0.80-1.05) | 0.219 | 0.48 (0.41-0.58) | p<0.001 | 0.63 (0.55-0.73) | p<0.001 |
| 25 + | 0.67 (0.42-1.07) | 0.092 | 0.32 (0.14-0.72) | 0.006 | 0.33 (0.19-0.58) | p<0.001 |
| **Maternal stature** |  |  |  |  |  |  |
| Very short | 2.22 (1.47-3.35) | p<0.001 | 0.93 (0.52-1.66) | 0.812 | 1.63 (1.08-2.45) | 0.018 |
| Short | 1.82 (1.59-2.08) | p<0.001 | 0.99 (0.82-1.19) | 0.939 | 1.44 (1.25-1.65) | p<0.001 |
| Normal | Ref. |  | Ref. |  | Ref. |  |
| **Maternal anemia** |  |  |  |  |  |  |
| Yes | 0.91 (0.79-1.05) | 0.227 | 1.16 (0.95-1.40) | 0.133 | 1.15 (0.99-1.33) | 0.051 |
| No | Ref. |  | Ref. |  | Ref. |  |
| **Place of delivery** |  |  |  |  |  |  |
| Home | 1.16 (0.86-1.55) | 0.329 | 0.90 (0.60-1.34) | 0.612 | 1.34 (0.97-1.84) | 0.074 |
| Health facility | Ref. |  | Ref. |  | Ref. |  |
| **Listening to radio** |  |  |  |  |  |  |
| Yes | Ref. |  | Ref. |  | Ref. |  |
| Not at all | 0.96 (0.84-1.09) | 0.551 | 1.34 (1.10-1.63) | 0.003 | 1.05 (0.91-1.21) | 0.461 |
| **Watching television** |  |  |  |  |  |  |
| Yes | Ref. |  | Ref. |  | Ref. |  |
| Not at all | 1.01 (0.85-1.19) | 0.921 | 1.67 (1.28-2.20) | p<0.001 | 1.13 (0.94-1.36) | 0.172 |
| ***Household factors*** |  |  |  |  |  |  |
| **Sex of the household head** |  |  |  |  |  |  |
| Male | Ref. |  | Ref. |  | Ref. |  |
| Female | 1.06 (0.90-1.24) | 0.488 | 1.15 (0.93-1.43) | 0.184 | 0.98 (0.83-1.16) | 0.829 |
| **Household size** |  |  |  |  |  |  |
| 1-4 | 0.97 (0.84-1.13) | 0.738 | 0.80 (0.64-1.01) | 0.052 | 0.91 (0.77-1.06) | 0.225 |
| 5+ | Ref. |  | Ref. |  | Ref. |  |
| ***Environmental factors*** |  |  |  |  |  |  |
| **Sanitation facility** |  |  |  |  |  |  |
| Improved | Ref. |  | Ref. |  | Ref. |  |
| Unimproved | 1.23 (0.85-1.79) | 0.262 | 0.91 (0.52-1.57) | 0.732 | 1.25 (0.84-1.88) | 0.264 |
| Open defecation | 1.28 (0.89-1.83) | 0.174 | 1.27 (0.76-2.13) | 0.364 | 1.46 (0.99-2.13) | 0.053 |
| **Source of drinking water** |  |  |  |  |  |  |
| Improved | Ref. |  | Ref. |  | Ref. |  |
| Unimproved | 1.05 (0.87-1.26) | 0.595 | 0.87 (0.68-1.11) | 0.281 | 0.88 (0.73-1.07) | 0.214 |
| **Time to get a water source** |  |  |  |  |  |  |
| On-premise | Ref. |  | Ref. |  | Ref. |  |
| ≤ 30 min | 1.17 (0.70-1.95) | 0.549 | 0.51 (0.28-0.95) | 0.034 | 0.71 (0.41-1.18) | 0.181 |
| 31-60 min | 1.12 (0.66-1.89) | 0.659 | 0.61 (0.32-1.15) | 0.128 | 0.73 (0.43-1.23) | 0.244 |
| >60 min | 1.22 (0.73-2.06) | 0.441 | 0.83 (0.45-1.55) | 0.565 | 0.84 (0.49-1.42) | 0.521 |
| **Child stool disposal** |  |  |  |  |  |  |
| Safe | Ref. |  | Ref. |  | Ref. |  |
| Unsafe | 1.13 (0.93-1.36) | 0.194 | 1.13 (0.86-1.48) | 0.361 | 1.11 (0.91-1.35) | 0.270 |
| ***Community-level characteristics*** |  |  |  |  |  |  |
| **Residence** |  |  |  |  |  |  |
| Urban | 0.39 (0.22-0.69) | 0.001 | 1.37 (0.72-2.58) | 0.335 | 0.58 (0.32-1.04) | 0.068 |
| Rural | Ref. |  | Ref. |  | Ref. |  |
| **Region** |  |  |  |  |  |  |
| Agrarian | 1.04 (0.73-1.49) | 0.810 | 1.20 (0.74-1.95) | 0.456 | 0.93 (0.64-1.35) | 0.703 |
| Pastoralist | 1.09 (0.75-1.60) | 0.639 | 1.66 (1.01-2.74) | 0.049 | 1.03 (0.69-1.53) | 0.874 |
| City administration | Ref. |  | Ref. |  | Ref. |  |

# Supplementary File 5 (b): Multilevel multivariable binary logistic regression analysis of factors associated with stunting in children aged 0-59 months in Ethiopia, EDHS-2011

|  | **Stunting** |  | **Model 1** | **Model 2** | **Model 3** | **Model 4** |
| --- | --- | --- | --- | --- | --- | --- |
|  | **Crude OR, 95%CI** | **p-values** |  | **AOR, 95%CI** | **AOR, 95%CI** | **AOR, 95%CI** |
| **Variables** |  |  |  |  |  |  |
| ***Child factors*** |  |  |  |  |  |  |
| **Sex** |  |  |  |  |  |  |
| Male | 1.13 (1.01-1.27) | 0.047 |  | 1.17 (1.02-1.34)* |  | 1.18 (1.03-1.35)* |
| Female | Ref. |  |  | Ref. |  | Ref. |
| **Age (months)** |  |  |  |  |  |  |
| **< 6** | 0.08 (0.06-0.11) | p<0.001 |  | 0.08 (0.06-0.11)** |  | 0.08 (0.06-0.11)** |
| 6-11 | 0.21 (0.16-0.27) | p<0.001 |  | 0.18 (0.14-0.24)** |  | 0.18 (0.14-0.24)** |
| 12-23 | 0.82 (0.69-0.97) | 0.027 |  | 0.74 (0.62-0.90)* |  | 0.75 (0.62-0.90)* |
| 24-35 | 1.34 (1.13-1.59) | 0.001 |  | 1.28 (1.07-1.54)* |  | 1.28 (1.07-1.54)* |
| 36-59 | Ref. |  |  | Ref. |  | Ref. |
| **Size of the child at birth** |  |  |  |  |  |  |
| Larger | Ref. |  |  | Ref. |  | Ref. |
| Average | 1.16 (0.99-1.36) | 0.057 |  | 1.20 (1.01-1.42)* |  | 1.99 (1.01-1.42)* |
| Small | 1.15 (0.98-1.35) | 0.075 |  | 1.36 (1.14-1.62)* |  | 1.36 (1.14-1.62)* |
| **Birth order** |  |  |  |  |  |  |
| First born | Ref. |  |  |  |  |  |
| 2-4 | 1.02 (0.86-1.22) | 0.784 |  |  |  |  |
| 5+ | 1.10 (0.92-1.32) | 0.295 |  |  |  |  |
| **Full vaccination** |  |  |  |  |  |  |
| Yes | Ref. |  |  | Ref. |  | Ref. |
| No | 0.61 (0.51-0.73) | p<0.001 |  | 0.91 (0.75-1.11) |  | 0.92 (0.75-1.11) |
| **Vitamin A last 6 months** |  |  |  |  |  |  |
| Yes | Ref. |  |  | Ref. |  | Ref. |
| No | 0.63 (0.56-0.72) | p<0.001 |  | 0.86 (0.74-0.99)* |  | 0.86 (0.74-0.99)* |
| **Currently breastfeeding** |  |  |  |  |  |  |
| Yes | Ref. |  |  | Ref. |  | Ref. |
| No | 1.67 (1.45-1.92) | p<0.001 |  | 0.86 (0.74-0.99)* |  | 1.01 (0.85-1.17) |
| **Early initiation of breastfeeding** |  |  |  |  |  |  |
| Yes | Ref. |  |  |  |  |  |
| No | 1.05 (0.89-1.22) | 0.538 |  |  |  |  |
| **Birth interval** |  |  |  |  |  |  |
| 7- 33 months / short/ | 0.91 (0.80-1.04) | 0.172 |  | 0.94 (0.69-1.29) |  | 0.94 (0.69-1.29) |
| ≥ 33 months /non-short/ | Ref. |  |  | Ref. |  | Ref. |
| **Diarrhoea** |  |  |  |  |  |  |
| Yes | 1.11 (0.94-1.32) | 0.196 |  | 1.28 (1.06-1.56)* |  | 1.29 (1.06-1.57)* |
| No | Ref. |  |  | Ref. |  | Ref. |
| **Fever** |  |  |  |  |  |  |
| Yes | 1.01 (0.87-1.18) | 0.839 |  |  |  |  |
| No | Ref. |  |  |  |  |  |
| **Cough** |  |  |  |  |  |  |
| Yes | 1.05 (0.90-1.22) | 0.538 |  |  |  |  |
| No | Ref. |  |  |  |  |  |
| ***Parental factors*** |  |  |  |  |  |  |
| **Mother's age** |  |  |  |  |  |  |
| 15-17 | 0.26 (0.12-0.57) | 0.001 |  | 0.48 (0.18-1.27) |  | 0.47 (0.18-1.27) |
| 18-24 | 0.85 (0.71-1.02) | 0.083 |  | 1.07 (0.74-1.56) |  | 1.08 (0.74-1.56) |
| 25-34 | 0.97 (0.84-1.13) | 0.753 |  | 1.12 (0.80-1.55) |  | 1.11 (0.80-1.55) |
| 35-49 | Ref. |  |  | Ref. |  | Ref. |
| **Mother's education** |  |  |  |  |  |  |
| No education | 1.23 (1.04-1.45) | 0.013 |  | 1.18 (0.97-1.42) |  | 1.16 (0.95-1.40) |
| Primary and above | Ref. |  |  | Ref. |  | Ref. |
| **Mother's currently working** |  |  |  |  |  |  |
| Yes | 1.04 (0.90-1.21) | 0.557 |  |  |  |  |
| No | Ref. |  |  |  |  |  |
| **Maternal BMI (kg/m^2^)** |  |  |  |  |  |  |
| <18.5 | Ref. |  |  | Ref. |  | Ref. |
| 18.5 to 24.9 | 0.92 (0.80-1.05) | 0.219 |  | 0.87 (0.74-1.01) |  | 0.86 (0.74-1.01) |
| 25 + | 0.67 (0.42-1.07) | 0.092 |  | 0.66 (0.39-1.12) |  | 0.70 (0.41-1.18) |
| **Maternal stature** |  |  |  |  |  |  |
| Very short | 2.22 (1.47-3.35) | p<0.001 |  | 3.37 (2.05-5.52)** |  | 3.41 (2.07-5.59)** |
| Short | 1.82 (1.59-2.08) | p<0.001 |  | 2.06 (1.77-2.39)** |  | 2.05 (1.76-2.38)** |
| Normal | Ref. |  |  | Ref. |  | Ref. |
| **Maternal anemia** |  |  |  |  |  |  |
| Yes | 0.91 (0.79-1.05) | 0.227 |  | 0.94 (0.80-1.09) |  | 0.94 (0.80-1.10) |
| No | Ref. |  |  | Ref. |  | Ref. |
| **Place of delivery** |  |  |  |  |  |  |
| Home | 1.16 (0.86-1.55) | 0.329 |  |  |  |  |
| Health facility | Ref. |  |  |  |  |  |
| **Listening to radio** |  |  |  |  |  |  |
| Yes | Ref. |  |  |  |  |  |
| Not at all | 0.96 (0.84-1.09) | 0.551 |  |  |  |  |
| **Watching television** |  |  |  |  |  |  |
| Yes | Ref. |  |  |  |  |  |
| Not at all | 1.01 (0.85-1.19) | 0.921 |  |  |  |  |
| ***Household factors*** |  |  |  |  |  |  |
| **Sex of the household head** |  |  |  |  |  |  |
| Male | Ref. |  |  |  |  |  |
| Female | 1.06 (0.90-1.24) | 0.488 |  |  |  |  |
| **Household size** |  |  |  |  |  |  |
| 1-4 | 0.97 (0.84-1.13) | 0.738 |  |  |  |  |
| 5+ | Ref. |  |  |  |  |  |
| ***Environmental factors*** |  |  |  |  |  |  |
| **Sanitation facility** |  |  |  |  |  |  |
| Improved | Ref. |  |  | Ref. |  | Ref. |
| Unimproved | 1.23 (0.85-1.79) | 0.262 |  | 1.15 (0.76-1.74) |  | 1.10 (0.73-1.66) |
| Open defecation | 1.28 (0.89-1.83) | 0.174 |  | 1.30 (0.87-1.93) |  | 1.23 (0.83-1.83) |
| **Source of drinking water** |  |  |  |  |  |  |
| Improved | Ref. |  |  |  |  |  |
| Unimproved | 1.05 (0.87-1.26) | 0.595 |  |  |  |  |
| **Time to get a water source** |  |  |  |  |  |  |
| On-premise | Ref. |  |  |  |  |  |
| ≤ 30 min | 1.17 (0.70-1.95) | 0.549 |  |  |  |  |
| 31-60 min | 1.12 (0.66-1.89) | 0.659 |  |  |  |  |
| >60 min | 1.22 (0.73-2.06) | 0.441 |  |  |  |  |
| **Child stool disposal** |  |  |  |  |  |  |
| Safe | Ref. |  |  |  |  |  |
| Unsafe | 1.13 (0.93-1.36) | 0.194 |  |  |  |  |
| ***Community-level characteristics*** |  |  |  |  |  |  |
| **Residence** |  |  |  |  |  |  |
| Urban | 0.39 (0.22-0.69) | 0.001 |  |  | 0.39 (0.23-0.69)* | 0.43 (0.23-0.78)* |
| Rural | Ref. |  |  |  | Ref. | Ref. |
| **Region** |  |  |  |  |  |  |
| Agrarian | 1.04 (0.73-1.49) | 0.810 |  |  | 1.06 (0.74-1.51) | 0.93 (0.63-1.36) |
| Pastoralist | 1.09 (0.75-1.60) | 0.639 |  |  | 1.09 (0.75-1.59) | 0.99 (0.67-1.49) |
| City administration | Ref. |  |  |  | Ref. | Ref. |
| **Random effects** |  |  |  |  |  |  |
| **Variance (SD)** |  |  | 0.2774 (0.0023) | 0.2468 (0.0032) | 0.2608 (0.0023) | 0.2306 (0.0033) |
| **ICC (%)** |  |  | 7.77 | 6.97 | 7.34 | 6.55 |
| **AIC** |  |  | 6442.587 | 5396.148 | 6437.271 | 5393.285 |
| **BIC** |  |  | 6455.503 | 5555.99 | 6469.563 | 5572.308 |
| **LL** |  |  | -3219.2935 | -2673.0742 | -3213.6356 | -2668.6426 |
| **Deviance** |  |  | 6,438.587 | 5,346.1484 | 6,427.2712 | 5,337.2852 |

# Supplementary File 5 (c): Multilevel multivariable binary logistic regression analysis of factors associated with wasting in children aged 0-59 months in Ethiopia, EDHS-2011

|  | **Wasting** |  | **Model 1** | **Model 2** | **Model 3** | **Model 4** |
| --- | --- | --- | --- | --- | --- | --- |
|  | **Crude OR, 95%CI** | **p-values** |  | **AOR, 95%CI** | **AOR, 95%CI** | **AOR, 95%CI** |
| **Variables** |  |  |  |  |  |  |
| ***Child factors*** |  |  |  |  |  |  |
| **Sex** |  |  |  |  |  |  |
| Male | 1.45 (1.22-1.72) | p<0.001 |  | 1.47 (1.23-1.77)** |  | 1.47 (1.22-1.76)** |
| Female | Ref. |  |  | Ref. |  | Ref. |
| **Age (months)** |  |  |  |  |  |  |
| **< 6** | 1.86 (1.40-2.47) | p<0.001 |  | 1.68 (1.22-2.31)* |  | 1.69 (1.22-2.33)* |
| 6-11 | 2.73 (2.07-3.59) | p<0.001 |  | 2.42 (1.78-3.28)** |  | 2.43 (1.79-3.30)** |
| 12-23 | 2.52 (2.01-3.17) | p<0.001 |  | 2.36 (1.83-3.04)** |  | 2.34 (1.82-3.03)** |
| 24-35 | 1.19 (0.92-1.54) | 0.173 |  | 1.02 (0.77-1.34) |  | 1.02 (0.77-1.34) |
| 36-59 | Ref. |  |  | Ref. |  | Ref. |
| **Size of the child at birth** |  |  |  |  |  |  |
| Larger | Ref. |  |  | Ref. |  | Ref. |
| Average | 1.08 (0.85-1.37) | 0.504 |  | 0.98 (0.77-1.25) |  | 0.98 (0.77-1.25) |
| Small | 1.82 (1.45-2.27) | p<0.001 |  | 1.55 (1.22-1.97)** |  | 1.55 (1.22-1.97)** |
| **Birth order** |  |  |  |  |  |  |
| First born | Ref. |  |  | Ref. |  | Ref. |
| 2-4 | 1.06 (0.82-1.37) | 0.669 |  | 0.97 (0.73-1.29) |  | 0.97 (0.73-1.29) |
| 5+ | 1.32 (1.02-1.70) | 0.034 |  | 1.12 (0.82-1.52) |  | 1.14 (0.83-1.55) |
| **Full vaccination** |  |  |  |  |  |  |
| Yes | Ref. |  |  | Ref. |  | Ref. |
| No | 1.47 (1.13-1.92) | 0.005 |  | 1.13 (0.85-1.51) |  | 1.06 (0.79-1.42) |
| **Vitamin A last 6 months** |  |  |  |  |  |  |
| Yes | Ref. |  |  | Ref. |  | Ref. |
| No | 1.28 (1.07-1.53) | 0.006 |  | 1.13 (0.92-1.37) |  | 1.10 (0.90-1.34) |
| **Currently breastfeeding** |  |  |  |  |  |  |
| Yes | Ref. |  |  | Ref. |  | Ref. |
| No | 0.74 (0.61-0.91) | 0.004 |  | 1.04 (0.83-1.32) |  | 1.03 (0.82-1.30) |
| **Early initiation of breastfeeding** |  |  |  |  |  |  |
| Yes | Ref. |  |  |  |  |  |
| No | 0.91 (0.74-1.11) | 0.359 |  |  |  |  |
| **Birth interval** |  |  |  |  |  |  |
| 7- 33 months / short/ | 1.07 (0.89-1.29) | 0.439 |  |  |  |  |
| ≥ 33 months /non-short/ | Ref. |  |  |  |  |  |
| **Diarrhoea** |  |  |  |  |  |  |
| Yes | 2.04 (1.65-2.50) | p<0.001 |  | 1.40 (1.10-1.78)* |  | 1.42 (1.12-1.81)* |
| No | Ref. |  |  | Ref. |  | Ref. |
| **Fever** |  |  |  |  |  |  |
| Yes | 1.84 (1.51-2.23) | p<0.001 |  | 1.66 (1.28-2.15)** |  | 1.65 (1.27-2.13)** |
| No | Ref. |  |  | Ref. |  |  |
| **Cough** |  |  |  |  |  |  |
| Yes | 1.22 (0.99-1.50) | 0.051 |  | 0.72 (0.55-0.94)* |  | 0.72 (0.55-0.93)* |
| No | Ref. |  |  | Ref. |  | Ref. |
| ***Parental factors*** |  |  |  |  |  |  |
| **Mother's age** |  |  |  |  |  |  |
| 15-17 | 1.25 (0.53-2.97) | 0.610 |  |  |  |  |
| 18-24 | 1.06 (0.83-1.35) | 0.638 |  |  |  |  |
| 25-34 | 1.04 (0.84-1.27) | 0.727 |  |  |  |  |
| 35-49 | Ref. |  |  |  |  |  |
| **Mother's education** |  |  |  |  |  |  |
| No education | 1.27 (0.99-1.61) | 0.052 |  | 1.17 (0.91-1.52) |  | 1.15 (0.88-1.49) |
| Primary and above | Ref. |  |  | Ref. |  | Ref. |
| **Mother's currently working** |  |  |  |  |  |  |
| Yes | 0.72 (0.58-0.90) | 0.004 |  | 0.82 (0.65-1.03) |  | 0.83 (0.65-1.04) |
| No | Ref. |  |  | Ref. |  | Ref. |
| **Maternal BMI (kg/m^2^)** |  |  |  |  |  |  |
| <18.5 | Ref. |  |  | Ref. |  | Ref. |
| 18.5 to 24.9 | 0.48 (0.41-0.58) | p<0.001 |  | 0.53 (0.44-0.64)** |  | 0.54 (0.45-0.65)** |
| 25 + | 0.32 (0.14-0.72) | 0.006 |  | 0.35 (0.15-0.81)* |  | 0.35 (0.15-0.82)* |
| **Maternal stature** |  |  |  |  |  |  |
| Very short | 0.93 (0.52-1.66) | 0.812 |  |  |  |  |
| Short | 0.99 (0.82-1.19) | 0.939 |  |  |  |  |
| Normal | Ref. |  |  |  |  |  |
| **Maternal anemia** |  |  |  |  |  |  |
| Yes | 1.16 (0.95-1.40) | 0.133 |  | 1.09 (0.89-1.34) |  | 1.09 (0.89-1.34) |
| No | Ref. |  |  | Ref. |  | Ref. |
| **Place of delivery** |  |  |  |  |  |  |
| Home | 0.90 (0.60-1.34) | 0.612 |  |  |  |  |
| Health facility | Ref. |  |  |  |  |  |
| **Listening to radio** |  |  |  |  |  |  |
| Yes | Ref. |  |  | Ref. |  | Ref. |
| Not at all | 1.34 (1.10-1.63) | 0.003 |  | 1.20 (0.96-1.49) |  | 1.25 (1.01-1.55)* |
| **Watching television** |  |  |  |  |  |  |
| Yes | Ref. |  |  | Ref. |  | Ref. |
| Not at all | 1.67 (1.28-2.20) | p<0.001 |  | 1.38 (1.02-1.86)* |  | 1.39 (1.03-1.87)** |
| ***Household factors*** |  |  |  |  |  |  |
| **Sex of the household head** |  |  |  |  |  |  |
| Male | Ref. |  |  | Ref. |  | Ref. |
| Female | 1.15 (0.93-1.43) | 0.184 |  | 1.16 (0.93-1.46) |  | 1.13 (0.90-1.42) |
| **Household size** |  |  |  |  |  |  |
| 1-4 | 0.80 (0.64-1.01) | 0.052 |  | 0.82 (0.63-1.06) |  | 0.81 (0.63-1.05) |
| 5+ | Ref. |  |  | Ref. |  | Ref. |
| ***Environmental factors*** |  |  |  |  |  |  |
| **Sanitation facility** |  |  |  |  |  |  |
| Improved | Ref. |  |  |  |  |  |
| Unimproved | 0.91 (0.52-1.57) | 0.732 |  |  |  |  |
| Open defecation | 1.27 (0.76-2.13) | 0.364 |  |  |  |  |
| **Source of drinking water** |  |  |  |  |  |  |
| Improved | Ref. |  |  |  |  |  |
| Unimproved | 0.87 (0.68-1.11) | 0.281 |  |  |  |  |
| **Time to get a water source** |  |  |  |  |  |  |
| On-premise | Ref. |  |  | Ref. |  | Ref. |
| ≤ 30 min | 0.51 (0.28-0.95) | 0.034 |  | 0.52 (0.27-1.02) |  | 0.53 (0.28-1.03) |
| 31-60 min | 0.61 (0.32-1.15) | 0.128 |  | 0.61 (0.31-1.19) |  | 0.61 (0.31-1.19) |
| >60 min | 0.83 (0.45-1.55) | 0.565 |  | 0.78 (0.40-1.50) |  | 0.77 (0.39-1.49) |
| **Child stool disposal** |  |  |  |  |  |  |
| Safe | Ref. |  |  |  |  |  |
| Unsafe | 1.13 (0.86-1.48) | 0.361 |  |  |  |  |
| ***Community-level characteristics*** |  |  |  |  |  |  |
| **Residence** |  |  |  |  |  |  |
| Urban | 1.37 (0.72-2.58) | 0.335 |  |  | 1.45 (0.77-2.75) | 1.16 (0.58-2.28) |
| Rural | Ref. |  |  |  | Ref. | Ref. |
| **Region** |  |  |  |  |  |  |
| Agrarian | 1.20 (0.74-1.95) | 0.456 |  |  | 1.19 (0.73-1.93) | 1.34 (0.81-2.20) |
| Pastoralist | 1.66 (1.01-2.74) | 0.049 |  |  | 1.66 (1.01-2.74)* | 1.75 (1.04-2.92)* |
| City administration | Ref. |  |  |  | Ref. | Ref. |
| **Random effects** |  |  |  |  |  |  |
| **Variance (SD)** |  |  | 0.2989 (0.0046) | 0.2149 (0.0063) | 0.2776 (0.0046) | 0.2079 (0.0063) |
| **ICC (%)** |  |  | 8.33 | 6.13 | 7.78 | 5.94 |
| **AIC** |  |  | 3833.877 | 3445.375 | 3829.7 | 3444.268 |
| **BIC** |  |  | 3846.793 | 3631.176 | 3861.992 | 3649.29 |
| **LL** |  |  | -1914.9384 | -1693.6874 | -1909.8501 | -1690.1341 |
| **Deviance** |  |  | 3,829.8768 | 3,387.3748 | 3,819.7002 | 3,380.2682 |

# Supplementary File 5 (d): Multilevel multivariable binary logistic regression analysis of factors associated with underweight in children aged 0-59 months in Ethiopia, EDHS-2011

|  | **Underweight** |  | **Model 1** | **Model 2** | **Model 3** | **Model 4** |
| --- | --- | --- | --- | --- | --- | --- |
|  | **Crude OR, 95%CI** | **p-values** |  | **AOR, 95%CI** | **AOR, 95%CI** | **AOR, 95%CI** |
| **Variables** |  |  |  |  |  |  |
| ***Child factors*** |  |  |  |  |  |  |
| **Sex** |  |  |  |  |  |  |
| Male | 1.27 (1.12-1.44) | p<0.001 |  | 1.34 (1.17-1.54)** |  | 1.34 (1.17-1.54)** |
| Female | Ref. |  |  | Ref. |  | Ref. |
| **Age (months)** |  |  |  |  |  |  |
| **< 6** | 0.17 (0.13-0.23) | p<0.001 |  | 0.13 (0.09-0.19)** |  | 0.13 (0.09-0.19)** |
| 6-11 | 0.54 (0.43-0.68) | p<0.001 |  | 0.40 (0.31-0.52)** |  | 0.40 (0.31-0.52)** |
| 12-23 | 1.01 (0.85-1.20) | 0.897 |  | 0.82 (0.67-1.01) |  | 0.82 (0.67-1.03) |
| 24-35 | 1.25 (1.05-1.48) | 0.009 |  | 1.14 (0.95-1.37) |  | 1.14 (0.95-1.37) |
| 36-59 | Ref. |  |  | Ref. |  | Ref. |
| **Size of the child at birth** |  |  |  |  |  |  |
| Larger | Ref. |  |  | Ref. |  | Ref. |
| Average | 1.27 (1.07-1.49) | 0.005 |  | 1.25 (1.05-1.50)* |  | 1.25 (1.05-1.50)* |
| Small | 1.68 (1.42-1.98) | p<0.001 |  | 1.86 (1.54-2.23)** |  | 1.86 (1.54-2.23)** |
| **Birth order** |  |  |  |  |  |  |
| First born | Ref. |  |  | Ref. |  | Ref. |
| 2-4 | 1.16 (0.97-1.40) | 0.107 |  | 1.10 (0.87-1.40) |  | 1.11 (0.87-1.40) |
| 5+ | 1.27 (1.05-1.54) | 0.011 |  | 1.29 (0.96-1.73) |  | 1.29 (0.96-1.74) |
| **Full vaccination** |  |  |  |  |  |  |
| Yes | Ref. |  |  | Ref. |  | Ref. |
| No | 0.83 (0.69-0.99) | 0.041 |  | 1.06 (0.82-1.23) |  | 1.01 (0.83-1.24) |
| **Vitamin A last 6 months** |  |  |  |  |  |  |
| Yes | Ref. |  |  | Ref. |  | Ref. |
| No | 0.89 (0.79-1.02) | 0.118 |  | 1.12 (0.96-1.29) |  | 1.12 (0.96-1.30) |
| **Currently breastfeeding** |  |  |  |  |  |  |
| Yes | Ref. |  |  | Ref. |  | Ref. |
| No | 1.27 (1.10-1.46) | 0.001 |  | 0.93 (0.79-1.09) |  | 0.93 (0.79-1.09) |
| **Early initiation of breastfeeding** |  |  |  |  |  |  |
| Yes | Ref. |  |  |  |  |  |
| No | 1.02 (0.87-1.19) | 0.788 |  |  |  |  |
| **Birth interval** |  |  |  |  |  |  |
| 7- 33 months / short/ | 0.92 (0.80-1.05) | 0.230 |  | 0.86 (0.63-1.19) |  | 0.87 (0.63-1.19) |
| ≥ 33 months /non-short/ | Ref. |  |  | Ref. |  | Ref. |
| **Diarrhoea** |  |  |  |  |  |  |
| Yes | 1.58 (1.33-1.88) | p<0.001 |  | 1.43 (1.16-1.75)* |  | 1.43 (1.17-1.75)* |
| No | Ref. |  |  | Ref. |  | Ref. |
| **Fever** |  |  |  |  |  |  |
| Yes | 1.46 (1.25-1.71) | p<0.001 |  | 1.42 (1.16-1.75)* |  | 1.43 (1.16-1.76)* |
| No | Ref. |  |  | Ref. |  | Ref. |
| **Cough** |  |  |  |  |  |  |
| Yes | 1.19 (1.02-1.39) | 0.025 |  | 0.95 (0.78-1.17) |  | 0.96 (0.78-1.18) |
| No | Ref. |  |  | Ref. |  | Ref. |
| ***Parental factors*** |  |  |  |  |  |  |
| **Mother's age** |  |  |  |  |  |  |
| 15-17 | 0.28 (0.12-0.67) | 0.004 |  | 0.67 (0.24-1.88) |  | 0.67 (0.24-1.86) |
| 18-24 | 0.82 (0.68-0.98) | 0.037 |  | 1.26 (0.85-1.89) |  | 1.26 (0.84-1.88) |
| 25-34 | 1.06 (0.91-1.24) | 0.407 |  | 1.41 (1.01-1.97)* |  | 1.41 (1.01-1.96)* |
| 35-49 | Ref. |  |  | Ref. |  | Ref. |
| **Mother's education** |  |  |  |  |  |  |
| No education | 1.52 (1.27-1.82) | p<0.001 |  | 1.38 (1.13-1.69)* |  | 1.37 (1.12-1.68)* |
| Primary and above | Ref. |  |  | Ref. |  | Ref. |
| **Mother's currently working** |  |  |  |  |  |  |
| Yes | 0.97 (0.83-1.13) | 0.699 |  |  |  |  |
| No | Ref. |  |  |  |  |  |
| **Maternal BMI (kg/m^2^)** |  |  |  |  |  |  |
| <18.5 | Ref. |  |  | Ref. |  | Ref. |
| 18.5 to 24.9 | 0.63 (0.55-0.73) | p<0.001 |  | 0.63 (0.54-0.73)** |  | 0.63 (0.54-0.73)** |
| 25 + | 0.33 (0.19-0.58) | p<0.001 |  | 0.34 (0.19-0.61)** |  | 0.35 (0.19-0.63)** |
| **Maternal stature** |  |  |  |  |  |  |
| Very short | 1.63 (1.08-2.45) | 0.018 |  | 2.08 (1.32-3.29)* |  | 2.09 (1.33-3.32)* |
| Short | 1.44 (1.25-1.65) | p<0.001 |  | 1.59 (1.37-1.86)** |  | 1.59 (1.36-1.85)** |
| Normal | Ref. |  |  | Ref. |  | Ref. |
| **Maternal anemia** |  |  |  |  |  |  |
| Yes | 1.15 (0.99-1.33) | 0.051 |  | 1.13 (0.96-1.33) |  | 1.13 (0.96-1.33) |
| No | Ref. |  |  | Ref. |  | Ref. |
| **Place of delivery** |  |  |  |  |  |  |
| Home | 1.34 (0.97-1.84) | 0.074 |  | 1.13 (0.79-1.62) |  | 1.11 (0.77-1.59) |
| Health facility | Ref. |  |  | Ref. |  | Ref. |
| **Listening to radio** |  |  |  |  |  |  |
| Yes | Ref. |  |  |  |  |  |
| Not at all | 1.05 (0.91-1.21) | 0.461 |  |  |  |  |
| **Watching television** |  |  |  |  |  |  |
| Yes | Ref. |  |  | Ref. |  | Ref. |
| Not at all | 1.13 (0.94-1.36) | 0.172 |  | 1.15 (0.94-1.41) |  | 1.16 (0.94-1.42) |
| ***Household factors*** |  |  |  |  |  |  |
| **Sex of the household head** |  |  |  |  |  |  |
| Male | Ref. |  |  |  |  |  |
| Female | 0.98 (0.83-1.16) | 0.829 |  |  |  |  |
| **Household size** |  |  |  |  |  |  |
| 1-4 | 0.91 (0.77-1.06) | 0.225 |  | 1.03 (0.85-1.25) |  | 1.03 (0.84-1.25) |
| 5+ | Ref. |  |  | Ref. |  | Ref. |
| ***Environmental factors*** |  |  |  |  |  |  |
| **Sanitation facility** |  |  |  |  |  |  |
| Improved | Ref. |  |  | Ref. |  | Ref. |
| Unimproved | 1.25 (0.84-1.88) | 0.264 |  | 1.30 (0.84-2.01) |  | 1.27 (0.82-1.96) |
| Open defecation | 1.46 (0.99-2.13) | 0.053 |  | 1.45 (0.96-2.19) |  | 1.41 (0.93-2.13) |
| **Source of drinking water** |  |  |  |  |  |  |
| Improved | Ref. |  |  | Ref. |  | Ref. |
| Unimproved | 0.88 (0.73-1.07) | 0.214 |  | 0.83 (0.67-1.02) |  | 0.83 (0.67-1.02) |
| **Time to get a water source** |  |  |  |  |  |  |
| On-premise | Ref. |  |  | Ref. |  | Ref. |
| ≤ 30 min | 0.71 (0.41-1.18) | 0.181 |  | 0.78 (0.44-1.40) |  | 0.75 (0.42-1.36) |
| 31-60 min | 0.73 (0.43-1.23) | 0.244 |  | 0.78 (0.43-1.41) |  | 0.76 (0.42-1.37) |
| >60 min | 0.84 (0.49-1.42) | 0.521 |  | 0.83 (0.46-1.50) |  | 0.79 (0.44-1.44) |
| **Child stool disposal** |  |  |  |  |  |  |
| Safe | Ref. |  |  |  |  |  |
| Unsafe | 1.11 (0.91-1.35) | 0.270 |  |  |  |  |
| ***Community-level characteristics*** |  |  |  |  |  |  |
| **Residence** |  |  |  |  |  |  |
| Urban | 0.58 (0.32-1.04) | 0.068 |  |  | 0.59 (0.33-1.06) | 0.61 (0.32-1.16) |
| Rural | Ref. |  |  |  | Ref. | Ref. |
| **Region** |  |  |  |  |  |  |
| Agrarian | 0.93 (0.64-1.35) | 0.703 |  |  | 0.94 (0.64-1.36) | 0.92 (0.61-1.39) |
| Pastoralist | 1.03 (0.69-1.53) | 0.874 |  |  | 1.03 (0.69-1.53) | 0.94 (0.61-1.45) |
| City administration | Ref. |  |  |  | Ref. | Ref. |
| **Random effects** |  |  |  |  |  |  |
| **Variance (SD)** |  |  | 0.3038 (0.0023) | 0.3151 (0.0029) | 0.2955 (0.0024) | 0.3101 (0.0029) |
| **ICC (%)** |  |  | 8.45 | 8.74 | 8.24 | 8.61 |
| **AIC** |  |  | 6088.787 | 5315.023 | 6090.459 | 5318.475 |
| **BIC** |  |  | 6101.703 | 5545.097 | 6122.75 | 5567.722 |
| **LL** |  |  | -3042.3934 | -2621.5114 | -3040.2294 | -2620.2374 |
| **Deviance** |  |  | 6,084.7868 | 5,243.0228 | 6,080.4588 | 5,240.4748 |
