# Supplementary material for: Factors associated with childhood undernutrition in poor Ethiopian households: Implications for public health interventions
Source: PLoS One. 2025 May 9;20(5):e0323332. doi: 10.1371/journal.pone.0323332 (PMC12063910; doi:10.1371/journal.pone.0323332)
Supplement: S6 File — (DOCX) [file pone.0323332.s006.docx]

**Supplementary File 6 (a): Multilevel bivariable binary logistic regression analysis of factors associated with stunting, wasting and underweight in children aged 0-59 months in Ethiopia, EDHS-2016.**

|  | **Stunting** |  | **Wasting** |  | **Underweight** |  |
| --- | --- | --- | --- | --- | --- | --- |
|  | **Crude OR, 95%CI** | **p-values** | **Crude OR, 95%CI** | **p-values** | **Crude OR, 95%CI** | **p-values** |
| **Variables** |  |  |  |  |  |  |
| ***Child factors*** |  |  |  |  |  |  |
| **Sex** |  |  |  |  |  |  |
| Male | 1.14 (1.01-1.29) | 0.037 | 1.24 (1.05-1.47) | 0.011 | 1.13 (0.99-1.28) | 0.060 |
| Female | Ref. |  | Ref. |  | Ref. |  |
| **Age (months)** |  |  |  |  |  |  |
| **< 6** | 0.17 (0.12-0.22) | p<0.001 | 1.59 (1.18-2.13) | 0.002 | 0.21 (0.16-0.29) | p<0.001 |
| 6-11 | 0.21 (0.16-0.28) | p<0.001 | 2.11 (1.61-2.76) | p<0.001 | 0.44 (0.35-0.57) | p<0.001 |
| 12-23 | 0.84 (0.71-1.01) | 0.052 | 2.07 (1.66-2.59) | p<0.001 | 0.92 (0.77-1.09) | 0.332 |
| 24-35 | 1.27 (1.08-1.51) | 0.004 | 1.01 (0.78-1.28) | 0.997 | 1.04 (0.88-1.23) | 0.624 |
| 36-59 | Ref. |  | Ref. |  | Ref. |  |
| **Size of the child at birth** |  |  |  |  |  |  |
| Larger | Ref. |  | Ref. |  | Ref. |  |
| Average | 1.22 (1.04-1.43) | 0.013 | 1.11 (0.89-1.39) | 0.331 | 1.31 (1.11-1.54) | 0.001 |
| Small | 1.30 (1.10-1.54) | 0.002 | 1.76 (1.41-2.21) | p<0.001 | 1.74 (1.46-2.07) | p<0.001 |
| **Birth order** |  |  |  |  |  |  |
| First born | Ref. |  | Ref. |  | Ref. |  |
| 2-4 | 0.98 (0.81-1.17) | 0.804 | 1.01 (0.78-1.28) | 0.987 | 1.07 (0.88-1.29) | 0.474 |
| 5+ | 1.05 (0.87-1.27) | 0.570 | 1.09 (0.84-1.39) | 0.500 | 1.13 (0.93-1.36) | 0.209 |
| **Full vaccination** |  |  |  |  |  |  |
| Yes | Ref. |  | Ref. |  | Ref. |  |
| No | 0.61 (0.49-0.74) | p<0.001 | 1.31 (0.99-1.71) | 0.052 | 0.92 (0.75-1.14) | 0.468 |
| **Vitamin A last 6 months** |  |  |  |  |  |  |
| Yes | Ref. |  | Ref. |  | Ref. |  |
| No | 0.88 (0.77-1.01) | 0.074 | 1.09 (0.91-1.30) | 0.344 | 1.02 (0.89-1.17) | 0.706 |
| **Currently breastfeeding** |  |  |  |  |  |  |
| Yes | Ref. |  | Ref. |  | Ref. |  |
| No | 1.23 (1.08-1.42) | 0.002 | 0.79 (0.65-0.95) | 0.013 | 1.14 (0.99-1.31) | 0.063 |
| **Early initiation of breastfeeding** |  |  |  |  |  |  |
| Yes | Ref. |  | Ref. |  | Ref. |  |
| No | 1.07 (0.90-1.26) | 0.441 | 1.14 (0.91-1.41) | 0.241 | 1.15 (0.97-1.37) | 0.107 |
| **Birth interval** |  |  |  |  |  |  |
| 7- 33 months / short/ | 0.90 (0.79-1.04) | 0.161 | 1.12 (0.93-1.36) | 0.220 | 0.95 (0.82-1.09) | 0.472 |
| ≥ 33 months /non-short/ | Ref. |  | Ref. |  | Ref. |  |
| **Diarrhoea** |  |  |  |  |  |  |
| Yes | 1.25 (1.03-1.53) | 0.024 | 1.22 (0.94-1.57) | 0.131 | 1.29 (1.06-1.58) | 0.010 |
| No | Ref. |  | Ref. |  | Ref. |  |
| **Fever** |  |  |  |  |  |  |
| Yes | 1.19 (1.01-1.43) | 0.050 | 1.39 (1.10-1.74) | 0.005 | 1.21 (1.01-1.45) | 0.040 |
| No | Ref. |  | Ref. |  | Ref. |  |
| **Cough** |  |  |  |  |  |  |
| Yes | 1.10 (0.93-1.31) | 0.257 | 1.21 (0.97-1.52) | 0.082 | 1.11 (0.93-1.32) | 0.247 |
| No | Ref. |  | Ref. |  | Ref. |  |
| ***Parental factors*** |  |  |  |  |  |  |
| **Mother's age** |  |  |  |  |  |  |
| 15-17 | 0.42 (0.19-0.94) | 0.035 | 1.63 (0.69-3.80) | 0.259 | 1.12 (0.54-2.28) | 0.759 |
| 18-24 | 0.82 (0.68-0.98) | 0.038 | 1.16 (0.91-1.48) | 0.227 | 0.88 (0.73-1.07) | 0.207 |
| 25-34 | 0.92 (0.78-1.07) | 0.285 | 1.07 (0.86-1.33) | 0.504 | 1.04 (0.89-1.22) | 0.621 |
| 35-49 | Ref. |  | Ref. |  | Ref. |  |
| **Mother's education** |  |  |  |  |  |  |
| No education | 1.19 (1.01-1.39) | 0.031 | 1.12 (0.89-1.39) | 0.312 | 1.44 (1.22-1.71) | p<0.001 |
| Primary and above | Ref. |  | Ref. |  | Ref. |  |
| **Mother's currently working** |  |  |  |  |  |  |
| Yes | 1.20 (1.03-1.40) | 0.022 | 0.98b(0.79-1.20) | 0.833 | 1.09 (0.93-1.27) | 0.297 |
| No | Ref. |  | Ref. |  | Ref. |  |
| **Maternal BMI (kg/m^2^)** |  |  |  |  |  |  |
| <18.5 | Ref. |  | Ref. |  | Ref. |  |
| 18.5 to 24.9 | 0.85 (0.74-0.98) | 0.028 | 0.65 (0.54-0.78) | p<0.001 | 0.61 (0.53-0.70) | p<0.001 |
| 25 + | 0.72 (0.51-1.01) | 0.056 | 0.57 (0.36-0.91) | 0.018 | 0.39 (0.27-0.57) | p<0.001 |
| **Maternal stature** |  |  |  |  |  |  |
| Very short | 1.69 (1.07-2.68) | 0.025 | 1.07 (0.57-2.01) | 0.829 | 1.95 (1.23-3.06) | 0.004 |
| Short | 1.62 (1.41-1.86) | p<0.001 | 0.97 (0.80-1.17) | 0.740 | 1.49 (1.29-1.71) | p<0.001 |
| Normal | Ref. |  | Ref. |  | Ref. |  |
| **Maternal anemia** |  |  |  |  |  |  |
| Yes | 1.03 (0.90-1.18) | 0.604 | 1.07 (0.89-1.28) | 0.440 | 1.13 (0.98-1.29) | 0.081 |
| No | Ref. |  | Ref. |  | Ref. |  |
| **Place of delivery** |  |  |  |  |  |  |
| Home | 1.27 (1.07-1.51) | 0.007 | 1.21 (0.95-1.54) | 0.119 | 1.36 (1.14-1.64) | 0.001 |
| Health facility | Ref. |  | Ref. |  | Ref. |  |
| **Listening to radio** |  |  |  |  |  |  |
| Yes | Ref. |  | Ref. |  | Ref. |  |
| Not at all | 1.01 (0.83-1.24) | 0.871 | 1.19 (0.90-1.58) | 0.208 | 1.01 (0.82-1.23) | 0.931 |
| **Watching television** |  |  |  |  |  |  |
| Yes | Ref. |  | Ref. |  | Ref. |  |
| Not at all | 0.93 (0.71-1.24) | 0.648 | 1.46 (0.95-2.23) | 0.081 | 0.89 (0.67-1.18) | 0.430 |
| ***Household factors*** |  |  |  |  |  |  |
| **Sex of the household head** |  |  |  |  |  |  |
| Male | Ref. |  | Ref. |  | Ref. |  |
| Female | 0.96 (0.82-1.12) | 0.624 | 1.29 (1.05-1.58) | 0.012 | 1.09 (0.93-1.28) | 0.273 |
| **Household size** |  |  |  |  |  |  |
| 1-4 | 0.86 (0.74-0.99) | 0.043 | 0.92 (0.76-1.13) | 0.462 | 0.89 (0.77-1.04) | 0.157 |
| 5+ | Ref. |  | Ref. |  | Ref. |  |
| ***Environmental factors*** |  |  |  |  |  |  |
| **Sanitation facility** |  |  |  |  |  |  |
| Improved | Ref. |  | Ref. |  | Ref. |  |
| Unimproved | 1.49 (1.06-2.09) | 0.022 | 0.87 (0.56-1.34) | 0.523 | 1.22 (0.86-1.72) | 0.257 |
| Open defecation | 1.40 (1.02-1.93) | 0.039 | 1.09 (0.73-1.63) | 0.662 | 1.27 (0.92-1.77) | 0.142 |
| **Source of drinking water** |  |  |  |  |  |  |
| Improved | Ref. |  | Ref. |  | Ref. |  |
| Unimproved | 0.93 (0.78-1.09) | 0.373 | 0.84 (0.68-1.05) | 0.137 | 0.87 (0.73-1.03) | 0.105 |
| **Time to get a water source** |  |  |  |  |  |  |
| On-premise | Ref. |  | Ref. |  | Ref. |  |
| ≤ 30 min | 1.01 (0.65-1.58) | 0.942 | 1.14 (0.61-2.11) | 0.674 | 0.87 (0.56-1.35) | 0.546 |
| 31-60 min | 1.17 (0.74-1.85) | 0.487 | 1.34 (0.71-2.52) | 0.358 | 0.97 (0.62-1.53) | 0.914 |
| >60 min | 1.03 (0.65-1.62) | 0.900 | 1.35 (0.72-2.55) | 0.349 | 1.01 (0.64-1.58) | 0.985 |
| **Child stool disposal** |  |  |  |  |  |  |
| Safe | Ref. |  | Ref. |  | Ref. |  |
| Unsafe | 1.27 (1.05-1.53) | 0.012 | 1.11 (0.85-1.43) | 0.426 | 1.25 (1.03-1.52) | 0.021 |
| ***Community-level characteristics*** |  |  |  |  |  |  |
| **Residence** |  |  |  |  |  |  |
| Urban | 1.02 (0.64-1.61) | 0.936 | 1.33 (0.77-2.31) | 0.304 | 0.90 (0.57-1.43) | 0.673 |
| Rural | Ref. |  | Ref. |  | Ref. |  |
| **Region** |  |  |  |  |  |  |
| Agrarian | 0.72 (0.51-1.02) | 0.069 | 0.98 (0.62-1.52) | 0.920 | 0.73 (0.52-1.01) | 0.063 |
| Pastoralist | 0.85 (0.59-1.24) | 0.410 | 1.21 (0.75-1.93) | 0.430 | 0.98 (0.67-1.39) | 0.904 |
| City administration | Ref. |  | Ref. |  | Ref. |  |

# Supplementary File 6 (b): Multilevel multivariable binary logistic regression analysis of factors associated with stunting in children aged 0-59 months in Ethiopia, EDHS-2016

|  | **Stunting** |  | **Model 1** | **Model 2** | **Model 3** | **Model 4** |
| --- | --- | --- | --- | --- | --- | --- |
|  | **Crude OR, 95%CI** | **p-values** |  | **AOR, 95%CI** | **AOR, 95%CI** | **AOR, 95%CI** |
| **Variables** |  |  |  |  |  |  |
| ***Child factors*** |  |  |  |  |  |  |
| **Sex** |  |  |  |  |  |  |
| Male | 1.14 (1.01-1.29) | 0.037 |  | 1.16 (0.97-1.39) |  | 1.16 (0.97-1.39) |
| Female | Ref. |  |  | Ref. |  | Ref. |
| **Age (months)** |  |  |  |  |  |  |
| **< 6** | 0.17 (0.12-0.22) | p<0.001 |  | 0.29 (0.12-0.68)* |  | 0.29 (0.12-0.67)* |
| 6-11 | 0.21 (0.16-0.28) | p<0.001 |  | 0.35 (0.15-0.81)* |  | 0.34 (0.15-0.79)* |
| 12-23 | 0.84 (0.71-1.01) | 0.052 |  | 1.45 (0.65-3.22) |  | 1.41 (0.63-3.14) |
| 24-35 | 1.27 (1.08-1.51) | 0.004 |  | 2.39 (1.07-5.33)* |  | 2.34 (1.05-5.20)* |
| 36-59 | Ref. |  |  | Ref. |  | Ref. |
| **Size of the child at birth** |  |  |  |  |  |  |
| Larger | Ref. |  |  | Ref. |  | Ref. |
| Average | 1.22 (1.04-1.43) | 0.013 |  | 1.42 (1.12-1.79)* |  | 1.44 (1.14-1.82)* |
| Small | 1.30 (1.10-1.54) | 0.002 |  | 1.57 (1.23-2.01)** |  | 1.57 (1.23-2.02)** |
| **Birth order** |  |  |  |  |  |  |
| First born | Ref. |  |  |  |  |  |
| 2-4 | 0.98 (0.81-1.17) | 0.804 |  |  |  |  |
| 5+ | 1.05 (0.87-1.27) | 0.570 |  |  |  |  |
| **Full vaccination** |  |  |  |  |  |  |
| Yes | Ref. |  |  | Ref. |  | Ref. |
| No | 0.61 (0.49-0.74) | p<0.001 |  | 0.93 (0.73-1.18) |  | 0.94 (0.74-1.19) |
| **Vitamin A last 6 months** |  |  |  |  |  |  |
| Yes | Ref. |  |  | Ref. |  | Ref. |
| No | 0.88 (0.77-1.01) | 0.074 |  | 1.05 (0.86-1.28) |  | 1.06 (0.86-1.28) |
| **Currently breastfeeding** |  |  |  |  |  |  |
| Yes | Ref. |  |  | Ref. |  | Ref. |
| No | 1.23 (1.08-1.42) | 0.002 |  | 0.89 (0.70-1.10) |  | 0.87 (0.69-1.09) |
| **Early initiation of breastfeeding** |  |  |  |  |  |  |
| Yes | Ref. |  |  |  |  |  |
| No | 1.07 (0.90-1.26) | 0.441 |  |  |  |  |
| **Birth interval** |  |  |  |  |  |  |
| 7- 33 months / short/ | 0.90 (0.79-1.04) | 0.161 |  | 1.31 (0.82-2.09) |  | 1.30 (0.81-2.09) |
| ≥ 33 months /non-short/ | Ref. |  |  | Ref. |  | Ref. |
| **Diarrhoea** |  |  |  |  |  |  |
| Yes | 1.25 (1.03-1.53) | 0.024 |  | 1.29 (0.98-1.71) |  | 1.29 (0.97-1.70) |
| No | Ref. |  |  | Ref. |  | Ref. |
| **Fever** |  |  |  |  |  |  |
| Yes | 1.19 (1.01-1.43) | 0.050 |  | 1.09 (0.84-1.41) |  | 1.09 (0.84-1.42) |
| No | Ref. |  |  | Ref. |  | Ref. |
| **Cough** |  |  |  |  |  |  |
| Yes | 1.10 (0.93-1.31) | 0.257 |  |  |  |  |
| No | Ref. |  |  |  |  |  |
| ***Parental factors*** |  |  |  |  |  |  |
| **Mother's age** |  |  |  |  |  |  |
| 15-17 | 0.42 (0.19-0.94) | 0.035 |  | 0.61 (0.22-1.74) |  | 0.63 (0.22-1.78) |
| 18-24 | 0.82 (0.68-0.98) | 0.038 |  | 0.72 (0.42-1.24) |  | 0.73 (0.42-1.25) |
| 25-34 | 0.92 (0.78-1.07) | 0.285 |  | 0.75 (0.46-1.22) |  | 0.75 (0.46-1.22) |
| 35-49 | Ref. |  |  | Ref. |  | Ref. |
| **Mother's education** |  |  |  |  |  |  |
| No education | 1.19 (1.01-1.39) | 0.031 |  | 1.06 (0.84-1.34) |  | 1.06 (0.84-1.34) |
| Primary and above | Ref. |  |  | Ref. |  | Ref. |
| **Mother's currently working** |  |  |  |  |  |  |
| Yes | 1.20 (1.03-1.40) | 0.022 |  | 1.04 (0.83-1.31) |  | 1.04 (0.83-1.32) |
| No | Ref. |  |  | Ref. |  | Ref. |
| **Maternal BMI (kg/m^2^)** |  |  |  |  |  |  |
| <18.5 | Ref. |  |  | Ref. |  | Ref. |
| 18.5 to 24.9 | 0.85 (0.74-0.98) | 0.028 |  | 0.79 (0.64-0.97)* |  | 0.79 (0.65-0.98)* |
| 25 + | 0.72 (0.51-1.01) | 0.056 |  | 0.63 (0.38-1.03) |  | 0.63 (0.38-1.04) |
| **Maternal stature** |  |  |  |  |  |  |
| Very short | 1.69 (1.07-2.68) | 0.025 |  | 2.18 (1.11-4.27)* |  | 2.17 (1.11-4.25)* |
| Short | 1.62 (1.41-1.86) | p<0.001 |  | 2.00 (1.64-2.44)** |  | 2.02 (1.65-2.47)** |
| Normal | Ref. |  |  | Ref. |  | Ref. |
| **Maternal anemia** |  |  |  |  |  |  |
| Yes | 1.03 (0.90-1.18) | 0.604 |  |  |  |  |
| No | Ref. |  |  |  |  |  |
| **Place of delivery** |  |  |  |  |  |  |
| Home | 1.27 (1.07-1.51) | 0.007 |  | 0.97 (0.76-1.23) |  | 0.98 (0.77-1.25) |
| Health facility | Ref. |  |  | Ref. |  | Ref. |
| **Listening to radio** |  |  |  |  |  |  |
| Yes | Ref. |  |  |  |  |  |
| Not at all | 1.01 (0.83-1.24) | 0.871 |  |  |  |  |
| **Watching television** |  |  |  |  |  |  |
| Yes | Ref. |  |  |  |  |  |
| Not at all | 0.93 (0.71-1.24) | 0.648 |  |  |  |  |
| ***Household factors*** |  |  |  |  |  |  |
| **Sex of the household head** |  |  |  |  |  |  |
| Male | Ref. |  |  |  |  |  |
| Female | 0.96 (0.82-1.12) | 0.624 |  |  |  |  |
| **Household size** |  |  |  |  |  |  |
| 1-4 | 0.86 (0.74-0.99) | 0.043 |  | 0.82 (0.65-1.04) |  | 0.83 (0.65-1.05) |
| 5+ | Ref. |  |  | Ref. |  | Ref. |
| ***Environmental factors*** |  |  |  |  |  |  |
| **Sanitation facility** |  |  |  |  |  |  |
| Improved | Ref. |  |  | Ref. |  | Ref. |
| Unimproved | 1.49 (1.06-2.09) | 0.022 |  | 2.18 (1.31-3.63)* |  | 2.16 (1.29-3.61)* |
| Open defecation | 1.40 (1.02-1.93) | 0.039 |  | 2.06 (1.26-3.37)* |  | 2.03 (1.24-3.33)* |
| **Source of drinking water** |  |  |  |  |  |  |
| Improved | Ref. |  |  |  |  |  |
| Unimproved | 0.93 (0.78-1.09) | 0.373 |  |  |  |  |
| **Time to get a water source** |  |  |  |  |  |  |
| On-premise | Ref. |  |  |  |  |  |
| ≤ 30 min | 1.01 (0.65-1.58) | 0.942 |  |  |  |  |
| 31-60 min | 1.17 (0.74-1.85) | 0.487 |  |  |  |  |
| >60 min | 1.03 (0.65-1.62) | 0.900 |  |  |  |  |
| **Child stool disposal** |  |  |  |  |  |  |
| Safe | Ref. |  |  | Ref. |  | Ref. |
| Unsafe | 1.27 (1.05-1.53) | 0.012 |  | 1.10 (0.84-1.45) |  | 1.11 (0.84-1.46) |
| ***Community-level characteristics*** |  |  |  |  |  |  |
| **Residence** |  |  |  |  |  |  |
| Urban | 1.02 (0.64-1.61) | 0.936 |  |  | 1.05 (0.67-1.67) | 1.31 (0.70-2.43) |
| Rural | Ref. |  |  |  | Ref. | Ref. |
| **Region** |  |  |  |  |  |  |
| Agrarian | 0.72 (0.51-1.02) | 0.069 |  |  | 0.72 (0.51-1.02) | 0.71 (0.46-1.09) |
| Pastoralist | 0.85 (0.59-1.24) | 0.410 |  |  | 0.85 (0.58-1.24) | 0.72 (0.45-1.14) |
| City administration | Ref. |  |  |  | Ref. | Ref. |
| **Random effects** |  |  |  |  |  |  |
| **Variance (SD)** |  |  | 0.3603 (0.0027) | 0.3339 (0.0066) | 0.3511 (0.0027) | 0.3215 (0.0067) |
| **ICC (%)** |  |  | 9.87 | 9.21 | 9.64 | 8.90 |
| **AIC** |  |  | 6250.397 | 3163.169 | 6251.175 | 3166.015 |
| **BIC** |  |  | 6263.296 | 3334.029 | 6283.422 | 3354.549 |
| **LL** |  |  | -3123.1987 | -1552.5847 | -3120.5875 | -1551.0073 |
| **Deviance** |  |  | 6,246.3974 | 3,105.1694 | 6,241.175 | 3,102.0146 |

# Supplementary File 6(c): Multilevel multivariable binary logistic regression analysis of factors associated with wasting in children aged 0-59 months in Ethiopia, EDHS-2016

|  | **Wasting** |  | **Model 1** | **Model 2** | **Model 3** | **Model 4** |
| --- | --- | --- | --- | --- | --- | --- |
|  | **Crude OR, 95%CI** | **p-values** |  | **AOR, 95%CI** | **AOR, 95%CI** | **AOR, 95%CI** |
| **Variables** |  |  |  |  |  |  |
| ***Child factors*** |  |  |  |  |  |  |
| **Sex** |  |  |  |  |  |  |
| Male | 1.24 (1.05-1.47) | 0.011 |  | 1.28 (1.02-1.61)* |  | 1.28 (1.02-1.61)* |
| Female | Ref. |  |  | Ref. |  | Ref. |
| **Age (months)** |  |  |  |  |  |  |
| **< 6** | 1.59 (1.18-2.13) | 0.002 |  | 4.03 (0.50-32.26) |  | 3.77 (0.47-30.22) |
| 6-11 | 2.11 (1.61-2.76) | p<0.001 |  | 5.14 (0.64-40.98) |  | 4.91 (0.61-39.14) |
| 12-23 | 2.07 (1.66-2.59) | p<0.001 |  | 5.40 (0.68-42.52) |  | 5.07 (0.64-29.93) |
| 24-35 | 1.01 (0.78-1.28) | 0.997 |  | 2.37 (0.30-18.74) |  | 2.23 (0.28-17.62) |
| 36-59 | Ref. |  |  | Ref. |  | Ref. |
| **Size of the child at birth** |  |  |  |  |  |  |
| Larger | Ref. |  |  | Ref. |  | Ref. |
| Average | 1.11 (0.89-1.39) | 0.331 |  | 1.04 (0.77-1.42) |  | 1.07 (0.79-1.45) |
| Small | 1.76 (1.41-2.21) | p<0.001 |  | 1.55 (1.13-2.10)* |  | 1.56 (1.14-2.12)* |
| **Birth order** |  |  |  |  |  |  |
| First born | Ref. |  |  |  |  |  |
| 2-4 | 1.01 (0.78-1.28) | 0.987 |  |  |  |  |
| 5+ | 1.09 (0.84-1.39) | 0.500 |  |  |  |  |
| **Full vaccination** |  |  |  |  |  |  |
| Yes | Ref. |  |  | Ref. |  | Ref. |
| No | 1.31 (0.99-1.71) | 0.052 |  | 1.01 (0.73-1.37) |  | 0.96 (0.70-1.32) |
| **Vitamin A last 6 months** |  |  |  |  |  |  |
| Yes | Ref. |  |  |  |  |  |
| No | 1.09 (0.91-1.30) | 0.344 |  |  |  |  |
| **Currently breastfeeding** |  |  |  |  |  |  |
| Yes | Ref. |  |  | Ref. |  | Ref. |
| No | 0.79 (0.65-0.95) | 0.013 |  | 1.03 (0.74-1.44) |  | 1.01 (0.73-1.42) |
| **Early initiation of breastfeeding** |  |  |  |  |  |  |
| Yes | Ref. |  |  | Ref. |  | Ref. |
| No | 1.14 (0.91-1.41) | 0.241 |  | 1.06 (0.82-1.35) |  | 1.04 (0.81-1.34) |
| **Birth interval** |  |  |  |  |  |  |
| 7- 33 months / short/ | 1.12 (0.93-1.36) | 0.220 |  | 1.61 (0.82-3.12) |  | 1.57 (0.81-3.04) |
| ≥ 33 months /non-short/ | Ref. |  |  | Ref. |  | Ref. |
| **Diarrhoea** |  |  |  |  |  |  |
| Yes | 1.22 (0.94-1.57) | 0.131 |  | 1.01 (0.72-1.42) |  | 1.01 (0.72-1.42) |
| No | Ref. |  |  | Ref. |  | Ref. |
| **Fever** |  |  |  |  |  |  |
| Yes | 1.39 (1.10-1.74) | 0.005 |  | 1.25 (0.87-1.80) |  | 1.26 (0.88-1.81) |
| No | Ref. |  |  | Ref. |  | Ref. |
| **Cough** |  |  |  |  |  |  |
| Yes | 1.21 (0.97-1.52) | 0.082 |  | 1.03 (0.72-1.45) |  | 1.03 (0.72-1.45) |
| No | Ref. |  |  | Ref. |  | Ref. |
| ***Parental factors*** |  |  |  |  |  |  |
| **Mother's age** |  |  |  |  |  |  |
| 15-17 | 1.63 (0.69-3.80) | 0.259 |  | 0.67 (0.21-2.12) |  | 0.69 (0.22-2.18) |
| 18-24 | 1.16 (0.91-1.48) | 0.227 |  | 0.55 (0.26-1.14) |  | 0.56 (0.27-1.18) |
| 25-34 | 1.07 (0.86-1.33) | 0.504 |  | 0.61 (0.31-1.22) |  | 0.63 (0.31-1.24) |
| 35-49 | Ref. |  |  | Ref. |  | Ref. |
| **Mother's education** |  |  |  |  |  |  |
| No education | 1.12 (0.89-1.39) | 0.312 |  |  |  |  |
| Primary and above | Ref. |  |  |  |  |  |
| **Mother's currently working** |  |  |  |  |  |  |
| Yes | 0.98(0.79-1.20) | 0.833 |  |  |  |  |
| No | Ref. |  |  |  |  |  |
| **Maternal BMI (kg/m^2^)** |  |  |  |  |  |  |
| <18.5 | Ref. |  |  | Ref. |  | Ref. |
| 18.5 to 24.9 | 0.65 (0.54-0.78) | p<0.001 |  | 0.76 (0.59-0.97)* |  | 0.77 (0.59-0.98)* |
| 25 + | 0.57 (0.36-0.91) | 0.018 |  | 0.72 (0.38-1.35) |  | 0.72 (0.38-1.35) |
| **Maternal stature** |  |  |  |  |  |  |
| Very short | 1.07 (0.57-2.01) | 0.829 |  |  |  |  |
| Short | 0.97 (0.80-1.17) | 0.740 |  |  |  |  |
| Normal | Ref. |  |  |  |  |  |
| **Maternal anemia** |  |  |  |  |  |  |
| Yes | 1.07 (0.89-1.28) | 0.440 |  |  |  |  |
| No | Ref. |  |  |  |  |  |
| **Place of delivery** |  |  |  |  |  |  |
| Home | 1.21 (0.95-1.54) | 0.119 |  | 1.37 (1.01-1.84)* |  | 1.38 (1.01-1.87)* |
| Health facility | Ref. |  |  | Ref. |  | Ref. |
| **Listening to radio** |  |  |  |  |  |  |
| Yes | Ref. |  |  | Ref. |  | Ref. |
| Not at all | 1.19 (0.90-1.58) | 0.208 |  | 1.16 (0.78-1.73) |  | 1.16 (0.78-1.74) |
| **Watching television** |  |  |  |  |  |  |
| Yes | Ref. |  |  | Ref. |  | Ref. |
| Not at all | 1.46 (0.95-2.23) | 0.081 |  | 1.26 (0.68-2.33) |  | 1.29 (0.69-2.38) |
| ***Household factors*** |  |  |  |  |  |  |
| **Sex of the household head** |  |  |  |  |  |  |
| Male | Ref. |  |  | Ref. |  | Ref. |
| Female | 1.29 (1.05-1.58) | 0.012 |  | 1.21 (0.92-1.58) |  | 1.18 (0.89-1.55) |
| **Household size** |  |  |  |  |  |  |
| 1-4 | 0.92 (0.76-1.13) | 0.462 |  |  |  |  |
| 5+ | Ref. |  |  |  |  |  |
| ***Environmental factors*** |  |  |  |  |  |  |
| **Sanitation facility** |  |  |  |  |  |  |
| Improved | Ref. |  |  |  |  |  |
| Unimproved | 0.87 (0.56-1.34) | 0.523 |  |  |  |  |
| Open defecation | 1.09 (0.73-1.63) | 0.662 |  |  |  |  |
| **Source of drinking water** |  |  |  |  |  |  |
| Improved | Ref. |  |  | Ref. |  | Ref. |
| Unimproved | 0.84 (0.68-1.05) | 0.137 |  | 0.84 (0.63-1.12) |  | 0.90 (0.67-1.21) |
| **Time to get a water source** |  |  |  |  |  |  |
| On-premise | Ref. |  |  |  |  |  |
| ≤ 30 min | 1.14 (0.61-2.11) | 0.674 |  |  |  |  |
| 31-60 min | 1.34 (0.71-2.52) | 0.358 |  |  |  |  |
| >60 min | 1.35 (0.72-2.55) | 0.349 |  |  |  |  |
| **Child stool disposal** |  |  |  |  |  |  |
| Safe | Ref. |  |  |  |  |  |
| Unsafe | 1.11 (0.85-1.43) | 0.426 |  |  |  |  |
| ***Community-level characteristics*** |  |  |  |  |  |  |
| **Residence** |  |  |  |  |  |  |
| Urban | 1.33 (0.77-2.31) | 0.304 |  |  | 1.42 (0.81-2.47) | 2.51 (1.25-5.07)* |
| Rural | Ref. |  |  |  | Ref. | Ref. |
| **Region** |  |  |  |  |  |  |
| Agrarian | 0.98 (0.62-1.52) | 0.920 |  |  | 0.97 (0.62-1.52) | 1.01 (0.58-1.73) |
| Pastoralist | 1.21 (0.75-1.93) | 0.430 |  |  | 1.23 (0.76-1.96) | 1.25 (0.70-2.23) |
| City administration | Ref. |  |  |  | Ref. | Ref. |
| **Random effects** |  |  |  |  |  |  |
| **Variance (SD)** |  |  | 0.4161 (0.0045) | 0.3273 (0.0114) | 0.4098 (0.0045) | 0.3017 (0.0116) |
| **ICC (%)** |  |  | 11.22 | 9.04 | 11.07 | 8.40 |
| **AIC** |  |  | 3954.682 | 2142.711 | 3956.308 | 2141.028 |
| **BIC** |  |  | 3967.611 | 2292.384 | 3988.632 | 2307.97 |
| **LL** |  |  | -1975.3408 | -1045.3557 | -1973.1541 | -1041.5141 |
| **Deviance** |  |  | 3,950.6816 | 2,090.7114 | 3,946.3082 | 2,083.0282 |

# Supplementary File 6 (d): Multilevel multivariable binary logistic regression analysis of factors associated with underweight in children aged 0-59 months in Ethiopia, EDHS-2016

|  | **Underweight** |  | **Model 1** | **Model 2** | **Model 3** | **Model 4** |
| --- | --- | --- | --- | --- | --- | --- |
|  | **Crude OR, 95%CI** | **p-values** |  | **AOR, 95%CI** | **AOR, 95%CI** | **AOR, 95%CI** |
| **Variables** |  |  |  |  |  |  |
| ***Child factors*** |  |  |  |  |  |  |
| **Sex** |  |  |  |  |  |  |
| Male | 1.13 (0.99-1.28) | 0.060 |  | 1.23 (1.04-1.47)* |  | 1.23 (1.04-1.47)* |
| Female | Ref. |  |  | Ref. |  | Ref. |
| **Age (months)** |  |  |  |  |  |  |
| **< 6** | 0.21 (0.16-0.29) | p<0.001 |  | 0.17 (0.11-0.25)** |  | 0.16 (0.11-0.25)** |
| 6-11 | 0.44 (0.35-0.57) | p<0.001 |  | 0.32 (0.22-0.46)** |  | 0.32 (0.22-0.46)** |
| 12-23 | 0.92 (0.77-1.09) | 0.332 |  | 0.76 (0.56-1.02) |  | 0.74 (0.55-1.01) |
| 24-35 | 1.04 (0.88-1.23) | 0.624 |  | 1.08 (0.83-1.41) |  | 1.07 (0.82-1.40) |
| 36-59 | Ref. |  |  | Ref. |  | Ref. |
| **Size of the child at birth** |  |  |  |  |  |  |
| Larger | Ref. |  |  | Ref. |  | Ref. |
| Average | 1.31 (1.11-1.54) | 0.001 |  | 1.49 (1.19-1.86)** |  | 1.50 (1.20-1.88)** |
| Small | 1.74 (1.46-2.07) | p<0.001 |  | 2.08 (1.64-2.65)** |  | 2.10 (1.65-2.67)** |
| **Birth order** |  |  |  |  |  |  |
| First born | Ref. |  |  | Ref. |  | Ref. |
| 2-4 | 1.07 (0.88-1.29) | 0.474 |  | 1.11 (0.82-1.52) |  | 1.11 (0.82-1.52) |
| 5+ | 1.13 (0.93-1.36) | 0.209 |  | 1.12 (0.76-1.64) |  | 1.12 (0.76-1.63) |
| **Full vaccination** |  |  |  |  |  |  |
| Yes | Ref. |  |  |  |  |  |
| No | 0.92 (0.75-1.14) | 0.468 |  |  |  |  |
| **Vitamin A last 6 months** |  |  |  |  |  |  |
| Yes | Ref. |  |  |  |  |  |
| No | 1.02 (0.89-1.17) | 0.706 |  |  |  |  |
| **Currently breastfeeding** |  |  |  |  |  |  |
| Yes | Ref. |  |  | Ref. |  | Ref. |
| No | 1.14 (0.99-1.31) | 0.063 |  | 0.79 (0.62-0.99)* |  | 0.78 (0.62-0.98)* |
| **Early initiation of breastfeeding** |  |  |  |  |  |  |
| Yes | Ref. |  |  | Ref. |  | Ref. |
| No | 1.15 (0.97-1.37) | 0.107 |  | 1.09 (0.90-1.32) |  | 1.09 (0.90-1.32) |
| **Birth interval** |  |  |  |  |  |  |
| 7- 33 months / short/ | 0.95 (0.82-1.09) | 0.472 |  |  |  |  |
| ≥ 33 months /non-short/ | Ref. |  |  |  |  |  |
| **Diarrhoea** |  |  |  |  |  |  |
| Yes | 1.29 (1.06-1.58) | 0.010 |  | 1.25 (0.96-1.62) |  | 1.25 (0.96-1.62) |
| No | Ref. |  |  | Ref. |  | Ref. |
| **Fever** |  |  |  |  |  |  |
| Yes | 1.21 (1.01-1.45) | 0.040 |  | 1.01 (0.75-1.34) |  | 1.01 (0.75-1.34) |
| No | Ref. |  |  | Ref. |  | Ref. |
| **Cough** |  |  |  |  |  |  |
| Yes | 1.11 (0.93-1.32) | 0.247 |  | 0.91 (0.69-1.19) |  | 0.92 (0.69-1.20) |
| No | Ref. |  |  | Ref. |  | Ref. |
| ***Parental factors*** |  |  |  |  |  |  |
| **Mother's age** |  |  |  |  |  |  |
| 15-17 | 1.12 (0.54-2.28) | 0.759 |  | 1.82 (0.72-4.60) |  | 1.84 (0.73-4.67) |
| 18-24 | 0.88 (0.73-1.07) | 0.207 |  | 1.21 (0.85-1.71) |  | 1.21 (0.86-1.72) |
| 25-34 | 1.04 (0.89-1.22) | 0.621 |  | 1.09 (0.86-1.38) |  | 1.09 (0.86-1.38) |
| 35-49 | Ref. |  |  | Ref. |  | Ref. |
| **Mother's education** |  |  |  |  |  |  |
| No education | 1.44 (1.22-1.71) | p<0.001 |  | 1.42 (1.12-1.80)* |  | 1.43 (1.13-1.82)* |
| Primary and above | Ref. |  |  | Ref. |  | Ref. |
| **Mother's currently working** |  |  |  |  |  |  |
| Yes | 1.09 (0.93-1.27) | 0.297 |  |  |  |  |
| No | Ref. |  |  |  |  |  |
| **Maternal BMI (kg/m^2^)** |  |  |  |  |  |  |
| <18.5 | Ref. |  |  | Ref. |  | Ref. |
| 18.5 to 24.9 | 0.61 (0.53-0.70) | p<0.001 |  | 0.52 (0.43-0.63)** |  | 0.52 (0.43-0.63)** |
| 25 + | 0.39 (0.27-0.57) | p<0.001 |  | 0.38 (0.23-0.63)** |  | 0.38 (0.23-0.63)** |
| **Maternal stature** |  |  |  |  |  |  |
| Very short | 1.95 (1.23-3.06) | 0.004 |  | 2.80 (1.59-4.93)** |  | 2.81 (1.60-4.94)** |
| Short | 1.49 (1.29-1.71) | p<0.001 |  | 1.77 (1.46-2.14)** |  | 1.78 (1.48-2.16)** |
| Normal | Ref. |  |  | Ref. |  | Ref. |
| **Maternal anemia** |  |  |  |  |  |  |
| Yes | 1.13 (0.98-1.29) | 0.081 |  | 1.28 (1.07-1.53)* |  | 1.27 (1.06-1.53)* |
| No | Ref. |  |  | Ref. |  | Ref. |
| **Place of delivery** |  |  |  |  |  |  |
| Home | 1.36 (1.14-1.64) | 0.001 |  | 1.14 (0.91-1.43) |  | 1.17 (0.92-1.47) |
| Health facility | Ref. |  |  | Ref. |  | Ref. |
| **Listening to radio** |  |  |  |  |  |  |
| Yes | Ref. |  |  |  |  |  |
| Not at all | 1.01 (0.82-1.23) | 0.931 |  |  |  |  |
| **Watching television** |  |  |  |  |  |  |
| Yes | Ref. |  |  |  |  |  |
| Not at all | 0.89 (0.67-1.18) | 0.430 |  |  |  |  |
| ***Household factors*** |  |  |  |  |  |  |
| **Sex of the household head** |  |  |  |  |  |  |
| Male | Ref. |  |  |  |  |  |
| Female | 1.09 (0.93-1.28) | 0.273 |  |  |  |  |
| **Household size** |  |  |  |  |  |  |
| 1-4 | 0.89 (0.77-1.04) | 0.157 |  | 0.79 (0.62-1.01) |  | 0.80 (0.63-1.01) |
| 5+ | Ref. |  |  | Ref. |  | Ref. |
| ***Environmental factors*** |  |  |  |  |  |  |
| **Sanitation facility** |  |  |  |  |  |  |
| Improved | Ref. |  |  | Ref. |  | Ref. |
| Unimproved | 1.22 (0.86-1.72) | 0.257 |  | 1.31 (0.82-2.09) |  | 1.31 (0.82-2.09) |
| Open defecation | 1.27 (0.92-1.77) | 0.142 |  | 1.15 (0.74-1.81) |  | 1.15 (0.73-1.80) |
| **Source of drinking water** |  |  |  |  |  |  |
| Improved | Ref. |  |  | Ref. |  | Ref. |
| Unimproved | 0.87 (0.73-1.03) | 0.105 |  | 0.81 (0.66-1.01) |  | 0.85 (0.68-1.06) |
| **Time to get a water source** |  |  |  |  |  |  |
| On-premise | Ref. |  |  |  |  |  |
| ≤ 30 min | 0.87 (0.56-1.35) | 0.546 |  |  |  |  |
| 31-60 min | 0.97 (0.62-1.53) | 0.914 |  |  |  |  |
| >60 min | 1.01 (0.64-1.58) | 0.985 |  |  |  |  |
| **Child stool disposal** |  |  |  |  |  |  |
| Safe | Ref. |  |  | Ref. |  | Ref. |
| Unsafe | 1.25 (1.03-1.52) | 0.021 |  | 1.15 (0.85-1.56) |  | 1.16 (0.86-1.57) |
| ***Community-level characteristics*** |  |  |  |  |  |  |
| **Residence** |  |  |  |  |  |  |
| Urban | 0.90 (0.57-1.43) | 0.673 |  |  | 0.98 (0.62-1.54) | 2.01 (1.14-3.55)* |
| Rural | Ref. |  |  |  | Ref. | Ref. |
| **Region** |  |  |  |  |  |  |
| Agrarian | 0.73 (0.52-1.01) | 0.063 |  |  | 0.73 (0.52-1.02) | 0.96 (0.63-1.45) |
| Pastoralist | 0.98 (0.67-1.39) | 0.904 |  |  | 0.98 (0.68-1.39) | 1.01 (0.64-1.55) |
| City administration | Ref. |  |  |  | Ref. | Ref. |
| **Random effects** |  |  |  |  |  |  |
| **Variance (SD)** |  |  | 0.2671 (0.0027) | 0.1967 (0.0066) | 0.2566 (0.0027) | 0.1922 (0.006) |
| **ICC (%)** |  |  | 7.51 | 5.64 | 7.23 | 5.52 |
| **AIC** |  |  | 5931.158 | 3320.882 | 5926.147 | 3321.182 |
| **BIC** |  |  | 5944.101 | 3506.454 | 5958.505 | 3524.712 |
| **LL** |  |  | -2963.5791 | -1629.4412 | -2958.0737 | -1626.591 |
| **Deviance** |  |  | 5,927.1582 | 3,258.8824 | 5,916.1474 | 3,253.182 |
